# α-(Aminomethyl)acrylates as acceptors in radical–polar crossover 1,4-additions of dialkylzincs: insights into enolate formation and trapping

**DOI:** 10.3762/bjoc.19.103

**Published:** 2023-09-21

**Authors:** Angel Palillero-Cisneros, Paola G Gordillo-Guerra, Fernando García-Alvarez, Olivier Jackowski, Franck Ferreira, Fabrice Chemla, Joel L Terán, Alejandro Perez-Luna

**Affiliations:** 1 Sorbonne Université, CNRS, Institut Parisien de Chimie Moléculaire, IPCM. 4 place Jussieu, 75005 Paris, France; 2 Benemérita Universidad Autonóma de Puebla, Instituto de Ciencias, ICUAP, Edificio IC-9, Complejo de Ciencias, C.U., 72570, Puebla, México; 3 (current adress) Universidad Autónoma Metropolitana, Unidad Xochimilco, Departamento de Sistemas Biológicos, Ciudad de México, C.P., 04690, México

**Keywords:** β-amino acids, tandem reactions, radical–polar crossover, *tert-*butanesulfinamide, zinc radical transfer

## Abstract

We demonstrate that α-(aminomethyl)acrylates are suitable acceptors for 1,4-additions of dialkylzincs in aerobic conditions. The air-promoted radical–polar crossover process involves the 1,4-addition of an alkyl radical followed by homolytic substitution at the zinc atom of dialkylzinc. Coordination of the nitrogen atom to zinc enables this S_H_2 process which represents a rare example of alkylzinc-group transfer to a tertiary α-carbonyl radical. The zinc enolate thus formed readily undergoes β-fragmentation unless it is trapped by electrophiles in situ. Enolates of substrates having free N–H bonds undergo protodemetalation to provide ultimately the 1,4-addition adduct. In the presence of carbonyl acceptors, aldol condensation occurs providing overall a tandem 1,4-addition–aldol process. When a *tert*-butanesulfinyl moiety is present on the nitrogen atom, these electrophilic substitution reactions occur with good levels of chiral induction, paving the way to enantioenriched β^2^-amino acids and β^2,2^-amino acids.

## Introduction

**Scheme 1:**
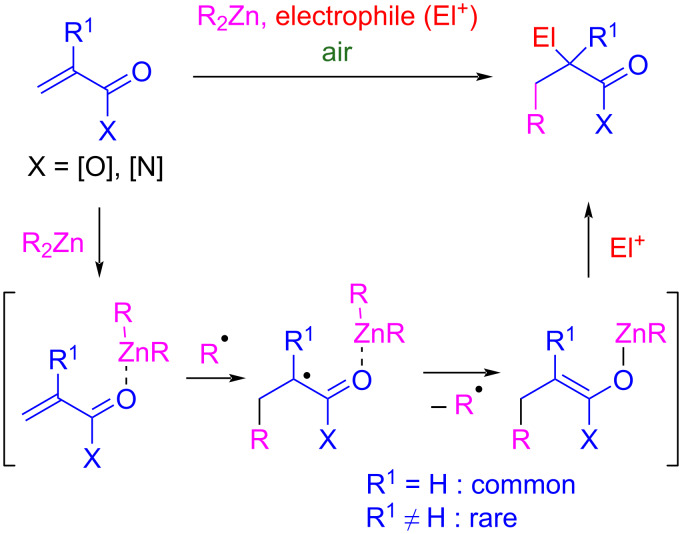
Air-promoted radical chain reaction of dialkylzinc reagents with α,β-unsaturated carbonyl compounds.

Dialkylzinc reagents react in aerobic medium with a range of α,β-unsaturated carbonyl compounds to provide the corresponding zinc enolates (Scheme 1) [1–2]. While simple, this reaction offers attractive features: 1) it proceeds under mild conditions in the absence of any transition-metal catalyst; 2) the 1,4-addition step can be combined with condensation reactions of the zinc enolate with electrophiles in protocols wherein all the reactive partners can be introduced from the start, given that dialkylzinc reagents offer a large functional group tolerance; and 3) the radical character of the process allows for the use of alkyl iodides as alkyl source in multicomponent reactions. Trialkylboranes can react in a similar way with enones [3] whereas, distinctively, suitable acceptors for the reaction with dialkylzinc reagents also include α,β-unsaturated carboxylic acid derivatives such as α,β-unsaturated (di)esters [4–5], *N*-enoyloxazolidinones [6–7], *N*-enoyloxazolidines [8], or alkylidenemalonates [9–11]. These reactions follow a free-radical chain process wherein alkyl radicals (R^•^) add across the C–C double bond of the 1,4-acceptor, activated by complexation with the dialkylzinc, to deliver an enoxyl radical that undergoes homolytic substitution at zinc (S_H_2) to produce a zinc enolate and a new R^•^ that propagates the radical chain (Scheme 1). Initiation occurs upon oxidation of the dialkylzinc reagent by oxygen.

**Scheme 2:**
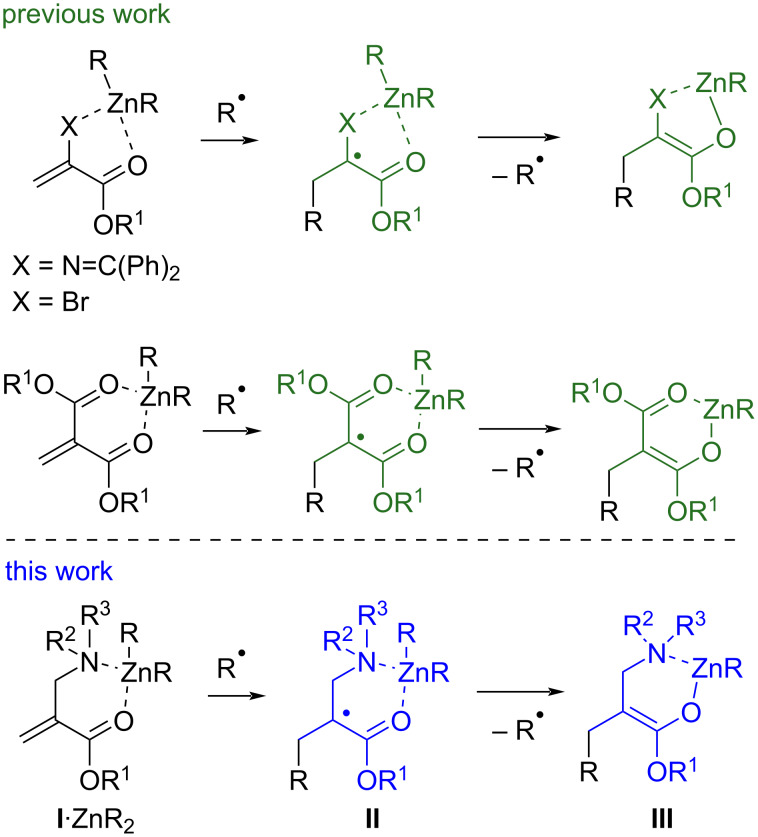
Enolate formation by zinc radical transfer (S_H_2 on dialkylzinc reagents).

The feasibility of such 1,4-addition reactions is fully reliant on the ease of the intermediate enoxyl radical to undergo alkylzinc-group transfer. Secondary α-carbonyl radicals (Scheme 1, R^1^ = H) undergo readily homolytic substitution. By contrast, tertiary α-carbonyl radicals (Scheme 1, R^1^ ≠ H) are less prone, making additions to α-substituted 1,4-acceptors more challenging. Typically, ethyl methacrylate does not react with dialkylzinc reagents [12]. Notwithstanding, 1,4-additions of dialkylzinc reagents have been reported with dehydroamino ester derivatives [13–14] and α-bromoacrylates [15], which both involve an S_H_2 at zinc of tertiary α-alkoxycarbonyl radicals (Scheme 2, top). Here, the key to unlock the reactivity is the presence of a Lewis-basic substituent coordinated to the zinc atom: this offers a gain in enthalpy associated to the formation of zinc enolates stabilized by chelation and increases the spin density delocalized at the oxygen atom involved in the chelate. Note that the reported 1,4-additions of dialkylzinc reagents to alkylidenemalonates could benefit from a similar effect, even though in this case, the direct formation of an intermediate enolate remains uncertain [11].

With this context in mind, we surmised that β-aminoenoates **I** could be suitable 1,4-acceptors (Scheme 2, bottom). We previously reported tandem reactions of such substrates wherein the intermediate enoxyl radical **II** arising from the addition step evolves via intramolecular addition to tethered alkenes [16–17] or alkynes [18]. We wondered if, in the absence of the pending radical acceptor, the presence of the β-nitrogen atom could nevertheless promote zinc enolate formation. Trapping of this enolate would lead to β-amino acid units, a class of compounds that has attracted a great deal of attention [19–24]. An obvious possible shortcoming that had to be considered was still that the generated zinc enolate **III** having a β-amino group could undergo β-elimination, thereby precluding its synthetic exploitation.

## Results and Discussion

### Preparation of α-(aminomethyl)acrylates

We commenced our study by preparing a selection of α-(aminomethyl)acrylates with variations of the nitrogen protecting group and the ester substituent. Towards this end, the direct allylation of primary amines **1**–**3** with methyl α-(bromomethyl)acrylate was contemplated first under several typical conditions that all afforded non-synthetically useful mixtures of mono- and diallylation, even if excess of the nitrogen nucleophiles was used. An alternative strategy was thus developed relying on the allylation of lithium (trimethylsilyl)amides prepared in situ from the parent amines by a lithiation/silylation/lithiation sequence (Table 1). Using this protocol, α-(aminomethyl)acrylates **5** and **6** derived from benzhydrylamine and aniline were prepared in high yields (Table 1, entries 1 and 2). The procedure was poorly efficient with tosylamine, leading to product **7** in low 20% yield [25].

**Table 1:** Preparation of α-(aminomethyl)acrylates with free N–H bonds.

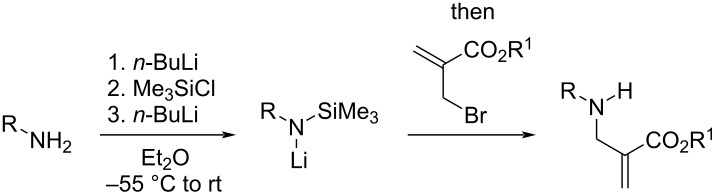				

Entry	Substrate (R)	R^1^	Product	Yield^a^

1	**1** (CH(Ph)_2_)	Me	**5**	81
2	**2** (Ph)	Me	**6**	80
3	**3** (Ts)	Me	**7**	20
4	**4** (S(O)*t-*Bu)	Me	**8a**	69
5	**4** (S(O)*t-*Bu)	*t-*Bu	**8b**	50
6	**4** (S(O)*t-*Bu)	Bn	**8c**	36

^a^Isolated yield.

**Scheme 3:**
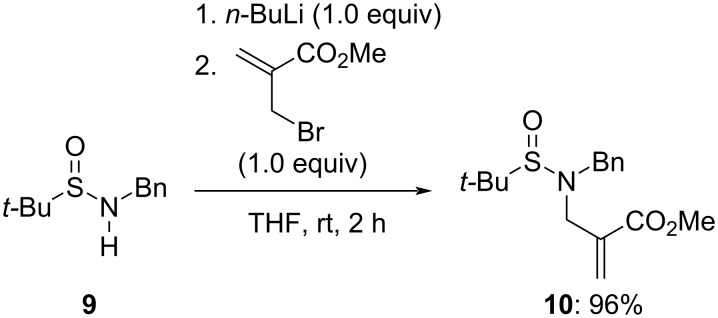
Preparation of α-(aminomethyl)acrylate **10**.

With the aim to develop asymmetric variants, we also considered the synthesis of *N*-(*tert*-butanesulfinyl) α-(aminomethyl)acrylates **8a**–**c**. For this purpose, the application of the same protocol with (±)-*tert*-butanesulfinamide (**4**) and the requisite α-(bromomethyl)acrylates gave satisfactory yields as well. Finally, *N*-benzyl-*N-*(*tert*-butanesulfinyl) α-(aminomethyl)acrylate **10** was prepared by allylation of lithiated *N*-benzyl *tert-*butanesulfinamide **9** (Scheme 3).

### 1,4-Addition reactions

**Scheme 4:**
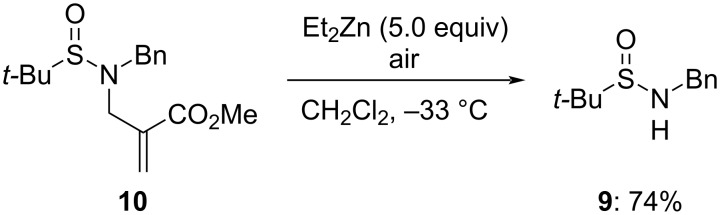
Reaction of α-(aminomethyl)acrylate **10** with Et_2_Zn in the presence of air.

Having the requisite α-(aminomethyl)acrylates in hands, we carried out an initial survey of their reaction with Et_2_Zn in CH_2_Cl_2_ at −33 °C on addition of air. In these conditions, acrylate **10** led to the recovery (following aqueous work-up) of sulfinamide **9** without traces of formation of the 1,4-adduct (Scheme 4).

By contrast, 1,4-addition without subsequent fragmentation was observed starting from α-(aminomethyl)acrylates having free N–H bonds (Table 2). The reaction of Et_2_Zn with acrylates **5**–**7** afforded the desired 1,4-addition products **11**–**13** in 42–55% yield. Better results were obtained starting from **8a**, which delivered adduct **14a** in 79% yield and 70:30 dr. We also noted that in the absence of deliberately added air, these reactions proceeded only with low conversion. For instance, starting from **8a**, product **14a** was obtained in only 25% yield along with ≈70% of starting material recovery.

**Table 2:** Air-promoted 1,4-addition of Et_2_Zn onto α-(aminomethyl)acrylates having free N–H bonds.^a^

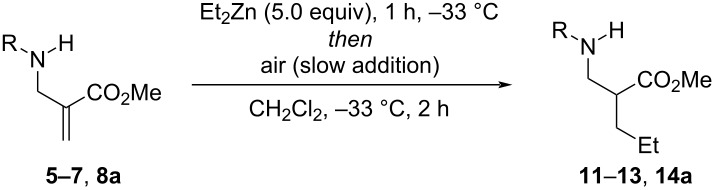				

Entry	Substrate (R)	Product	Yield^b^	dr^c^

1	**5** (CH(Ph)_2_)	**11**	42	
2	**6** (Ph)	**12**	55	
3	**7** (Ts)	**13**	55	
4	**8a** (S(O)*t-*Bu)	**14a**	76	70:30
5	**8a** (S(O)*t-*Bu)	**14a**	25^d^	50:50

^a^General conditions: α-(aminomethyl)acrylate (0.2 mmol), Et_2_Zn (1.0 mmol), CH_2_Cl_2_ (6 mL), air (20 mL introduced via syringe at 0.5 mL·min^−1^ rate). ^b^Isolated yield. ^c^Ratio of diastereomers measured by ^1^H NMR spectroscopy prior to purification. ^d^No air was added.

These results are relevant in the sense that not only they demonstrate that the oxygen-promoted 1,4-addition of α-(aminomethyl)acrylates with free N–H bonds is a productive process, but also that the *tert*-butanesulfinyl moiety is well tolerated and that 1,4-stereoinduction can be achieved. Hence, in order to improve the levels of diastereoselectivity, we investigated further the reaction conditions starting with enoate **8a** as model substrate (Table 3).

**Table 3:** Optimization of the air-promoted 1,4-addition of dialkylzinc reagents onto *N*-(*tert-*butanesulfinyl) α-(aminomethyl)acrylates.

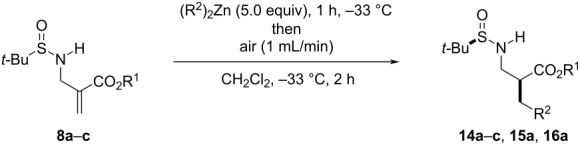						

Entry	Substrate (R^1^)	R^2^	Product	Variation of conditions^a^	Yield^b^	dr^c,d^

1	**8a** (Me)	Et	**14a**	none	76	70:30
2	**8a** (Me)	Et	**14a**	−78 °C instead of −33 °C	60	57:43
3	**8a** (Me)	Et	**14a**	oxygen (5 mL) was added at once immediately after Et_2_Zn	83	59:41
4	**8a** (Me)	Et	**14a**	toluene instead of CH_2_Cl_2_	82	75:25
5	**8a** (Me)	Et	**14a**	hexane instead of CH_2_Cl_2_	82	85:15
6	**8b** (*t-*Bu)	Et	**14b**	hexane instead of CH_2_Cl_2_	88	85:15
7	**8c** (Bn)	Et	**14c**	none	76	70:30
8	**8a** (Me)	Bu	**15a**	none	71	67:33
9	**8a** (Me)	Me	**16a**	none	n.r.^e^	
10	**8a** (Me)	Me	**16a**	hexane instead of CH_2_Cl_2_	n.r.^e^	

^a^All reactions were conducted at a 0.2 mmol scale using 20 mL of air. ^b^Isolated yield (mixture of diastereoisomers). ^c^Measured by ^1^H or ^13^C NMR spectroscopy prior to purification. ^d^The relative configuration of the major diastereomer is shown in the scheme; it was determined by chemical correlation for **14b** (see below) and inferred by analogy for **14a** and **14c**. ^e^No reaction.

Carrying out the reaction at −78 °C instead of −33 °C was deleterious both for the yield and the selectivity (Table 3, entry 2). By contrast, we rapidly learned that leaving diethylzinc in contact with the starting acrylate for 1 h prior to the addition of air had a significant impact on the stereoselectivity. When air was introduced directly after Et_2_Zn (Table 3, entry 3) a much lower 59:41 dr was observed. This behavior was suggestive of the need for coordination of diethylzinc both to the carbonyl and sulfinyl unit to achieve good levels of selectivity. Hence, to reinforce Lewis pair formation, the reaction was also carried out in apolar solvents such as toluene and hexane (entries 4 and 5 in Table 3). In hexane, an 88% yield with 85:15 dr was obtained, which constituted the best conditions. Importantly, the protocol was found to be similarly applicable with enoates **8b** (Table 3, entry 6) and **8c** (entry 7) having *tert*-butyl and benzyl ester groups, which, as the methyl ester unit, are typical in the context of amino acid synthesis. ZnBu_2_ was also amenable to 1,4-addition (Table 3, entry 8), but not ZnMe_2_ (entries 9 and 10). This difference can be ascribed to a less favorable homolytic substitution reaction of ZnMe_2_ in relation to its higher analogues and is in line with previous literature observations [11].

**Scheme 5:**
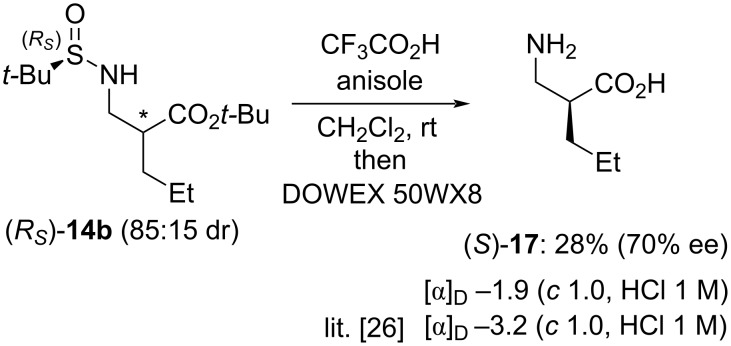
Chemical correlation to determine the configuration of the major diastereomer of (*R_S_*)-**14b**.

The configuration of the major diastereomer was determined by chemical correlation (Scheme 5). Product (*R_S_*)-**14b** (85:15 dr), i.e., a mixture of two enantiomerically pure diastereomers, was obtained from (*R_S_*)-*tert-*butylsulfinamide upon allylation with *tert*-butyl α-(bromomethyl)acrylate followed by 1,4-addition with Et_2_Zn. It was then converted into the known β^2^-amino acid **17** by TFA-promoted concomitant deprotection of the nitrogen and the ester groups. The sample was found to have a negative optical rotation, thereby indicating that the major enantiomer present had *S* configuration [26]. This allowed to establish that the configuration of the major diastereomer present in (*R_S_*)-**14b** was (*R_S_*,*S*), and thus the sense of chiral induction for the 1,4-addition reactions reported in Table 2.

### Tandem 1,4-addition–aldol condensation reactions

**Scheme 6:**
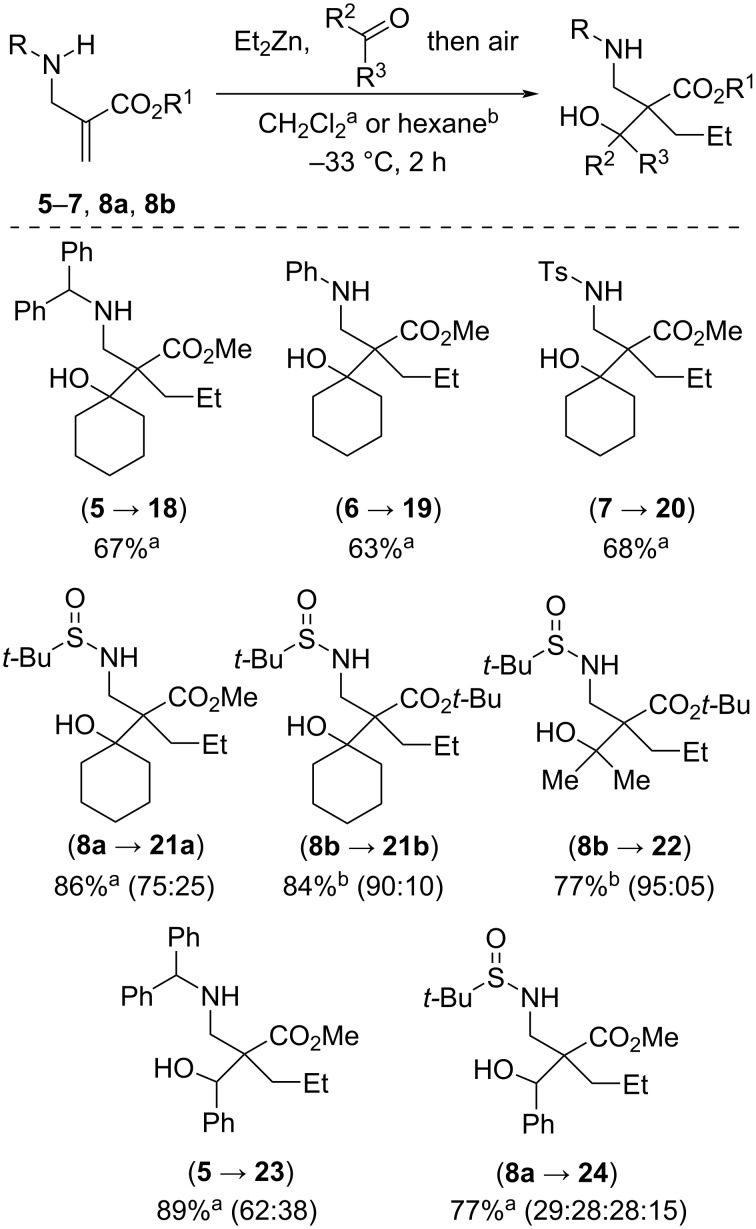
Air-promoted tandem 1,4-addition–aldol condensation reactions of Et_2_Zn with α-(aminomethyl)acrylates.

We then went on to consider tandem 1,4-addition–aldol condensation reactions (Scheme 6), which offer the interesting prospect of generating an all-carbon quaternary stereocenter. α-(Aminomethyl)acrylates **5**–**7** reacted smoothly at −33 °C within 2 h with Et_2_Zn in the presence of cyclohexanone to afford amino alcohols **18**–**20** in quite good yields (63–68%). Even better yields were obtained with enoates **8a** and **8b** both with cyclohexanone and acetone as carbonyl partners. Starting from **8a** and carrying out the reaction in CH_2_Cl_2_, product **21a** was obtained in 86% yield with 75:25 dr. Alike for the 1,4-addition protocol, better stereoinduction was obtained by performing the reaction in hexane: **8b** was converted into **21b** and **22** in 77–84% yield with higher than 90:10 dr. It is also interesting to note that the levels of induction for the 1,4-addition–aldol condensations are somewhat higher than those obtained for the 1,4-additions. Aldehydes also proved competent terminal electrophiles for the tandem sequence. Illustratively, adducts **23** and **24** were obtained from α-(aminomethyl)acrylates **5** and **8a** in 77–88% yields, albeit as poorly selective mixtures of diastereoisomers. This lack of stereocontrol is not surprising, given the well-known difficulty to control the relative configuration between the two adjacent stereocenters created during aldol condensations with zinc enolates.

### Mechanistic insights

**Scheme 7:**
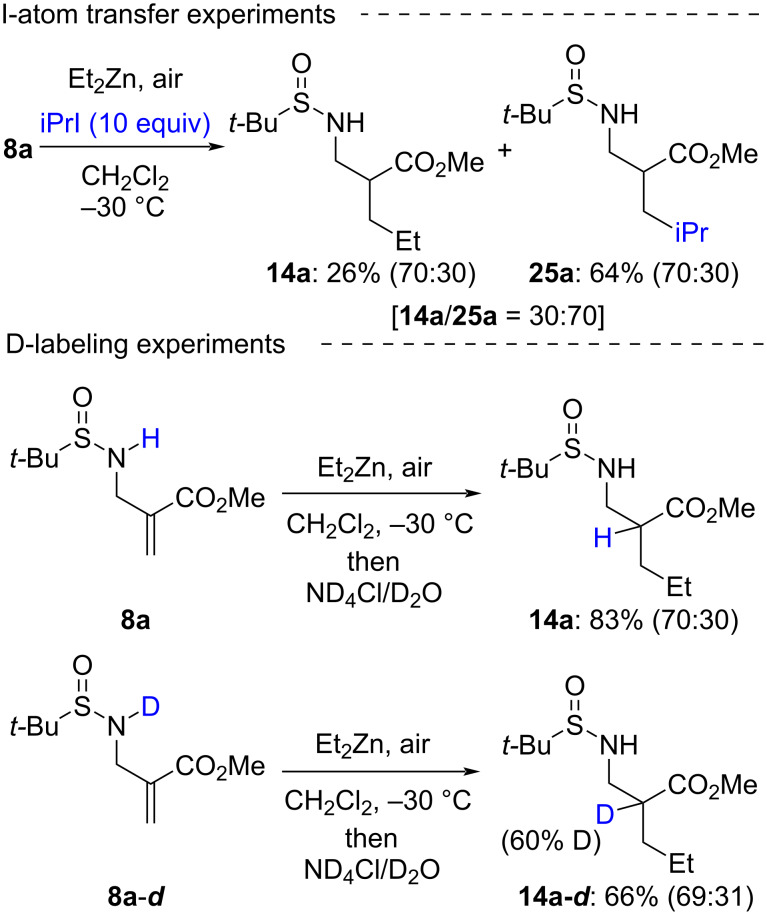
Diagnostic experiments for a radical mechanism and for enolate formation.

The last part of our work was devoted to gain mechanistic insight for the developed reaction protocols through several diagnostic experiments. Regarding the 1,4-addition process, the lower reactivity noted in the absence of air (Table 2, entry 5) represents already a strong indication for a radical addition mechanism. This is further supported by the result of an I-atom transfer experiment (Scheme 7, top). In the presence of two equivalents of iPrI, the reaction of **8a** with Et_2_Zn leads to a mixture of product **14a** and product **25a**, incorporating an iPr moiety, in a **14a**/**25a** 30:70 mixture. Product **25a** is formed on addition of an iPr radical generated by I-atom transfer from iPrI to the Et radical, and is diagnostic for the formation of the latter in the reaction medium.

Deuterium labeling experiments were then performed to substantiate the formation of a zinc enolate following radical addition (Scheme 7, bottom). Much to our surprise however, no deuterium incorporation is observed on quenching with ND_4_Cl/D_2_O the reaction between **8a** and Et_2_Zn. By contrast, a significant deuterium incorporation is obtained when deuterated starting material (**8a-*d***) is engaged. The combination of these two results is in agreement with the formation of a zinc enolate that undergoes proto- (or deuterio)demetalation with the N–H (or N–D) as proton (or deuterium) source.

**Scheme 8:**
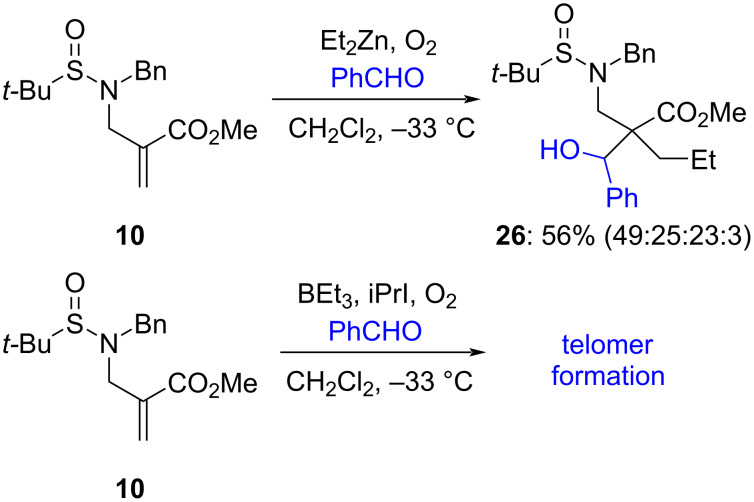
Diagnostic experiments with *N*-benzyl enoate **10**.

To further analyze the influence of the presence of an N–H function, we performed other reactions with *N*-benzyl enoate **10** which proved highly informative. As discussed previously (Scheme 4), application of the developed protocol for 1,4-addition to **10** only yields *N*-benzyl-*N*-*tert*-butylsulfinamide following β-elimination. By contrast, in the presence of benzaldehyde, 1,4-addition–aldol condensation is predominant, yielding **26** in 56% yield as a 49:25:23:3 diastereomeric mixture (Scheme 8). When **10** is exposed to Et_3_B in the presence of iPrI, benzaldehyde, and O_2_, which are conditions known to promote radical 1,4-addition, only formation of telomers [7] is noted. This lends clear evidence that the intermediate enoxyl radical does not intervene neither in the β-fragmentation (Scheme 4) nor in the addition across the carbonyl bonds.

**Scheme 9:**
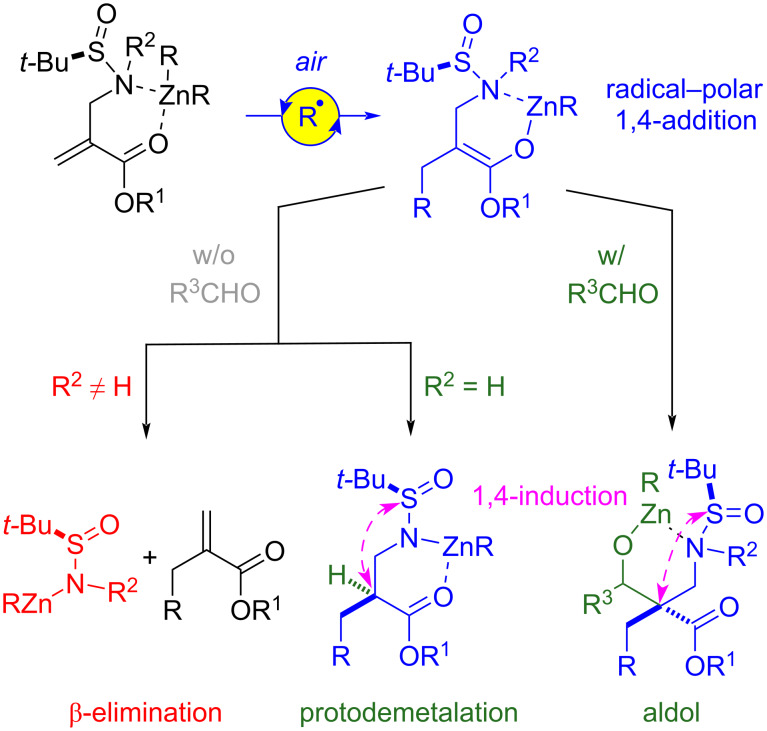
Reactivity manifolds for the air-promoted tandem 1,4-addition–electrophilic substitution reaction between dialkylzinc reagents and α-(aminomethyl)acrylates (*N*-(*tert-*butanesulfinyl) derivatives shown).

Overall, the mechanistic investigations support the scenario depicted in Scheme 9. Oxygen (in air) triggers a free-radical chain reaction between α-(aminomethyl)acrylates and dialkylzinc reagents that entails 1,4-addition and S_H_2 of the formed enoxyl radical facilitated by coordination of nitrogen to zinc. The zinc enolate thus formed evolves following different pathways according to the type of substrate and reaction conditions. In the absence of a carbonyl electrophile, enolates of substrates with trisubstituted nitrogen groups undergo β-fragmentation. By contrast, those derived from substrates having N–H bonds undergo protodemetalation to provide ultimately the 1,4-addition adduct. In the presence of carbonyl acceptors, these two competitive reactions are superseded and the enolate engages in aldol condensation regardless of its nitrogen substitution; the outcome of the reaction is a tandem 1,4-addition–aldol process. When the *tert*-butanesulfinyl moiety is present on the nitrogen atom, electrophilic substitution of the intermediate enolates (protodemetalation or aldol condensation) occurs with decent levels of chiral induction. It should be mentioned here that our attempts to trap the intermediate enolate with a carbon electrophile other than carbonyl acceptors (i.e., iodomethane) were not successful and protodemetalation of the enolate outcompeted methylation.

## Conclusion

In conclusion, we have demonstrated that α-(aminomethyl)acrylates are suitable acceptors for 1,4-additions with dialkylzincs in aerobic conditions. Coordination of the nitrogen atom to zinc is crucial to enable the S_H_2 step of the tertiary α-carbonyl radical that follows radical 1,4-addition in order to deliver a zinc enolate. The latter is poised to undergo β-fragmentation, but this process can be outcompeted by in situ electrophilic substitution reactions which offer synthetically useful procedures: 1,4-addition (for substrates having N–H bonds) or tandem 1,4-addition–aldol reactions (in the presence of carbonyl electrophiles). Asymmetric variants of these transformations are possible using the *tert-*butanesulfinyl chiral auxiliary on the nitrogen atom. The levels of 1,4-stereoinduction are significant but a convincing model to account for it cannot be put forward at this point. Nonetheless, from a synthetic methodology point of view, the reported protocols are relevant as they offer a new, direct and modular route to enantioenriched α-mono- and α,α-disubstituted β-amino acids (β^2^-amino acids and β^2,2^-amino acids), with, for the latter, the noteworthy stereocontrolled construction of an all-carbon quaternary stereocenter. Furthermore, our protocol provides a complement to existing literature, as none of the previously reported methods to convert α-(aminomethyl)acrylates into enantioenriched β-amino acids is applicable for the preparation of β^2,2^-amino acids [27–31].

## Experimental

**1. Procedure for the monoallylation of primary amines and *tert*-butylsulfinamide (preparation of compounds 5–7 and 8a–c).** In a round-bottomed flask under argon, *n*-BuLi (1.0 equiv, soln. in heptane) was added dropwise to a THF (0.2 mol·L^−1^) solution of the appropriate primary amine or *tert*-butylsulfinamide (1.0 equiv) at −55 °C. The mixture was then stirred at rt for 30 min, cooled to −55 °C, and trimethylsilyl chloride (1.0 equiv) was added. The mixture was then stirred at rt for 30 min, cooled to −55 °C, and *n*-BuLi (1.0 equiv, soln. in heptane) was added dropwise. The mixture was stirred at rt for 30 min, cooled to −78 °C, and the corresponding α-(bromomethyl)acrylate (1.0 equiv) was added. The reaction mixture was then stirred for 2 h letting the temperature rise to rt and quenched with aq 1 M NH_4_Cl. The aqueous layer was extracted with EtOAc (3×) and the combined organic layer was washed (brine), dried (MgSO_4_), and concentrated under reduced pressure to provide the crude product which was then purified by column chromatography on silica gel.

**2. Procedure for the air-promoted 1,4-addition of dialkylzinc reagents to α-(aminomethyl)acrylates (preparation of compounds 11–13, 14a–c, and 15a).** In a Schlenk-tube under argon, the appropriate α-(aminomethyl)acrylate (0.2 mmol) was dissolved in the indicated reaction solvent (3 mL) and the solution was cooled to −33 °C. Then, Et_2_Zn (1 M in hexanes, 1.0 mL, 1.0 mmol) was added dropwise and the solution was stirred for 1 h. Air (20 mL) was introduced directly into the solution via a syringe fitted with a CaCl_2_ pad at a 0.5 mL/min rate (syringe pump). After the end of the air addition, the mixture was stirred for an additional 80 min at −33 °C and then quenched with aq NH_4_Cl (5 mL) at 0 °C. The aqueous layer was extracted with CH_2_Cl_2_ (2×). The combined organic layer was washed (brine), dried (MgSO_4_), and concentrated under reduced pressure to provide the crude product which was then purified by column chromatography on silica gel.

**3. Procedure for the air-promoted tandem 1,4-addition–aldol reaction between dialkylzinc reagents, α-(aminomethyl)acrylates and carbonyl derivatives (preparation of compounds 18–20, 21a–b, 22–24).** In a Schlenk-tube under argon, the appropriate α-(aminomethyl)acrylate (0.2 mmol) was dissolved in the indicated reaction solvent (3 mL) and the solution was cooled to −33 °C. The carbonyl electrophile (1.0 mmol) and then Et_2_Zn (1 M in hexanes, 1.0 mL, 1.0 mmol) were added dropwise and the solution was stirred for 1 h. Air (20 mL) was introduced directly into the solution via a syringe fitted with a CaCl_2_ pad at a 0.5 mL/min rate (syringe pump). After the end of the air addition, the mixture was stirred for an additional 80 min at −33 °C and then quenched with aq NH_4_Cl (5 mL) at 0 °C. The aqueous layer was extracted with CH_2_Cl_2_ (2×). The combined organic layer was washed (brine), dried (MgSO_4_), and concentrated under reduced pressure to provide the crude product which was then purified by column chromatography on silica gel.

## Supporting Information

File 1 General information, characterization data, chemical correlation, and copies of NMR spectra.
