# Supplementary material for: α-(Aminomethyl)acrylates as acceptors in radical–polar crossover 1,4-additions of dialkylzincs: insights into enolate formation and trapping
Source: Beilstein J Org Chem. 2023 Sep 21;19:1443–51. doi: 10.3762/bjoc.19.103 (PMC10520473; doi:10.3762/bjoc.19.103)

BEILSTEIN JOURNAL OF ORGANIC CHEMISTRY

Supporting Information

for

α-(Aminomethyl)acrylates as acceptors in radical–polar crossover 1,4-additions of dialkylzincs: insights into enolate formation and trapping

Angel Palillero-Cisneros, Paola G. Gordillo-Guerra, Fernando García-Alvarez, Olivier Jackowski, Franck Ferreira, Fabrice Chemla, Joel L. Terán and Alejandro Perez-Luna

Beilstein J. Org. Chem. **2023,** *19,* 1443–1451. doi:10.3762/bjoc.19.103

General information, characterization data, chemical correlation, and copies of NMR spectra

License and Terms: This is a supporting information file under the terms of the Creative Commons Attribution License (https://creativecommons.org/licenses/by/4.0). Please note that the reuse, redistribution and reproduction in particular requires that the author(s) and source are credited and that individual graphics may be subject to special legal provisions.
The license is subject to the *Beilstein Journal of Organic Chemistry* terms and conditions: (https://www.beilstein-journals.org/bjoc/terms)

Table of contents

I. General information S2

II. Preparation of α-(aminomethyl)acrylates 5, 6, 7, 8a–c, and 10 S2

III. Air-promoted 1,4-addition of dialkylzinc reagents to α-(aminomethyl)acrylates S4

a. Preparation and characterization of compounds 11, 12, 13, 14a–c, and 15a S4

b. Chemical correlation to determine the sense of 1,4-stereoinduction in the 1,4-addition reaction S7

IV. Air-promoted tandem 1,4-addition–aldol reaction between dialkylzinc reagents, α-(aminomethyl)acrylates, and carbonyl derivatives (preparation and characterization of compounds 18, 19, 20, 21a, 21b, 22, 23, and 24) S7

V. Diagnostic experiments S10

a. I-atom transfer experiment (preparation and characterization of 25a) S10

b. D-labeling experiments S11

c. Air-promoted tandem 1,4-addition–aldol condensation between Et_2Zn, *N*-benzyl α-(aminomethyl)acrylate 10, and benzaldehyde S11

VI. ^{1}H NMR and ^{13}C NMR spectra of new compounds S12

I. General information

All reactions were carried out under an argon atmosphere by standard syringe and septa techniques. Glassware was flame-dried under vacuum or taken directly from the oven (100 °C) and let cool under vacuum prior to every use. Reagents and solvents were purchased from commercial sources and generally used as received. CH_2Cl_2 and Et_2O were dried on an MBraun purification system MB SPS-800. THF from the MB SPS-800 was distilled over sodium and benzophenone under nitrogen flow prior to utilization.

NMR spectra (^1H, ^{13}C) were recorded on a Bruker AVANCE 400 MHz spectrometer at Plateforme RMN Moléculaire of Sorbonne Université. NMR experiments were carried out at room temperature in $CDCl_3$. Chemical shifts are given in parts per million (ppm) using the $CDCl_3$ residual non-deuterated signals as reference (δ ^1H = 7.26 ppm; δ ^{13}C = 77.16 ppm). The terms m, s, d, t, q and br s represent multiplet, singlet, doublet, triplet, quartet and broad singlet, respectively. Coupling constants (*J*) are given in hertz (Hz).

High-resolution mass spectra (ESI–MS) were acquired using an LTQ-Orbitrap XL from Thermo Scientific (Thermo Fisher Scientific, Courtaboeuf, France) operated in positive ionization mode. TLC analyses were performed on Merck 60 F254 silica gel and revealed with either an ultra-violet lamp (λ = 254 nm) or a specific color reagent (potassium permanganate, *p*-anisaldehyde, etc.). Purifications by flash column chromatography were performed using silica gel Merck Geduran® SI 60 (40–63 µm).

II. Preparation of α-(aminomethyl)acrylates 5, 6, 7, 8a–c, and 10

General procedure for the monoallylation of primary amines or amides (GP1): In a round-bottomed flask under argon, *n*-BuLi (1.0 equiv, soln. in heptane) was added dropwise to a THF (0.2 mol·L^{-1}) solution of the appropriate amine or amide derivative (1.0 equiv) at −55 °C. The mixture was then stirred at rt for 30 min, cooled back to −55 °C, and trimethylsilyl chloride (1.0 equiv) was added. The mixture was then stirred at rt for 30 min, cooled back to −55 °C, and *n*-BuLi (1.0 equiv, soln. in heptane) was added dropwise. The mixture was stirred at rt for 30 min, cooled to −78 °C, and the corresponding 2-(bromomethyl)acrylate (1.0 equiv) was added. The reaction mixture was then stirred for 2 h letting the temperature rise to rt and quenched with aq. 1 M NH_4Cl. The aqueous layer was extracted with EtOAc (× 3) and the combined organics were washed (brine), dried ($MgSO_4$) and concentrated under reduced pressure to provide the crude product which was then purified by column chromatography on silica gel.

5

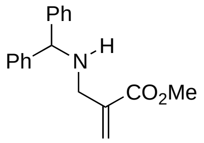

Chemical Formula: $C_{18}H_{19}NO_2$
Molecular Weight: 281,35

Methyl [*N*-diphenylmethyl(aminomethyl)acrylate]

Prepared according to **GP1** from diphenylmethylamine (**1**, 220 mg, 1.2 mmol) and methyl 2-(bromomethyl)acrylate (214 mg, 1.2 mmol). Purification by silica-gel column chromatography [cyclohexane/AcOEt, 99:1 to 90:10] yielded the title compound (275 mg, 81%) as a pale-yellow oil. **^{1}H NMR** (400 MHz, $CDCl_3$) δ 7.48–7.38 (m, 4H), 7.36–7.27 (m, 4H), 7.25–7.19 (m, 2H), 6.30 (s, 1H), 5.72 (s, 1H), 4.87 (s, 1H), 3.76 (s, 3H), 3.47 (s, 2H), 1.98 (br s, 1H (N*H*)). **^{13}C NMR** (101 MHz, $CDCl_3$) δ 167.3, 143.9, 138.4, 128.6, 127.4, 127.2, 126.4, 66.1, 51.9, 48.5. **HRMS** (ESI): *m/z* calculated for $C_{18}H_{19}NO_2Na$ $[M+Na]^+$ 304.1308, found 304.1298. **IR**: ν (cm^{-1}) 3025, 2359, 2340, 1714, 1492, 1452, 1435, 1152, 698.

6

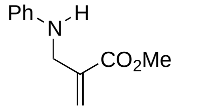

Chemical Formula: $C_{11}H_{13}NO_2$
Molecular Weight: 191,23

Methyl [*N*-phenyl(aminomethyl)acrylate]

Prepared according to **GP1** from phenylamine (**2**, 112 mg, 1.2 mmol) and methyl 2-(bromomethyl)acrylate (214 mg, 1.2 mmol). Purification by silica-gel column chromatography [cyclohexane/AcOEt, 99:1 to 90:10] yielded the title compound (184 mg, 80%) as a yellow oil. NMR characterization data was in good agreement with that previously reported [1].

7

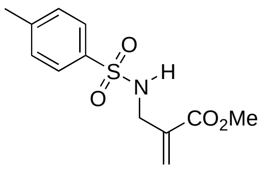

Chemical Formula: $C_{12}H_{15}NO_4S$
Molecular Weight: 269,32

Methyl [*N*-tosyl(aminomethyl)acrylate]

Prepared according to **GP1** from tosylamine (**3**, 171 mg, 1.0 mmol) and methyl 2-(bromomethyl)acrylate (214 mg, 1.2 mmol). Purification by silica-gel column chromatography [cyclohexane/AcOEt, 99:1 to 75:25] yielded the title compound (54 mg, 20%) as a yellow oil. **^{1}H NMR** (400 MHz, $CDCl_3$) δ 7.76–7.67 (m, 2H), 7.28 (d, *J* = 8.4 Hz, 2H), 6.19–6.15 (m, 1H), 5.79–5.73 (m, 1H), 5.32–5.12 (m, 1H (N*H*)), 3.80 (d, *J* = 6.6 Hz, 2H), 3.69 (s, 3H), 2.41 (s, 3H). **^{13}C NMR** (101 MHz, $CDCl_3$) δ 166.3, 143.6, 137.3, 135.5, 129.8, 128.0, 127.2, 52.2, 44.6, 21.6. **HRMS** (ESI): *m/z* calculated for $C_{12}H_{15}NO_4SNa$ $[M+Na]^+$ 292.0614, found 292.0621. **IR** ν (cm^{-1}) 1713, 1438, 1329, 1151, 812, 658.

8a

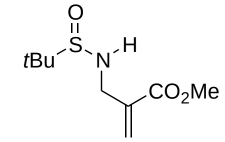

Chemical Formula: $C_9H_{17}NO_3S$
Molecular Weight: 219,30

(±)-Methyl [*N*-*tert*-butanesulfinyl(aminomethyl)acrylate]

Prepared according to **GP1** from (±)-*tert*-butanesulfinamide (**4**, 242 mg, 2.0 mmol) and methyl 2-(bromomethyl)acrylate (356 mg, 2.0 mmol). Purification by silica-gel column chromatography [cyclohexane/AcOEt, 99:1 to 80:20] yielded the title compound (303 mg, 69%) as a yellowish oil. **^{1}H NMR** (400 MHz, $CDCl_3$) δ 6.20 (s, 1H), 5.78 (s, 1H), 3.95 (dd, *J* = 15.3, 6.6 Hz, 1H (AB system)), 3.87 (dd, *J* = 15.3, 6.6 Hz, 1H (AB system)), 3.73–3.65 (m, 1H (N*H*)), 3.71 (s, 3H), 1.14 (s, 9H). **^{13}C NMR** (101 MHz, $CDCl_3$) δ 166.5, 138.1, 126.9, 55.9, 52.0, 47.0, 22.6. **HRMS** (ESI): *m/z* calculated for $C_9H_{17}NO_3SNa$ $[M+Na]^+$: 242.0816, found 242.0821**.** **IR**: ν (cm^{-1}) 1714, 1635, 1436, 1153, 1050, 814, 598.

8b

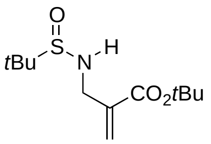

Chemical Formula: $C_{12}H_{23}NO_3S$
Molecular Weight: 261,38

(±)-*tert*-Butyl [*N*-*tert*-butanesulfinyl(aminomethyl)acrylate]

Prepared according to **GP1** from (±)-*tert*-butanesulfinamide (**4**, 242 mg, 2.0 mmol) and *tert*-butyl 2-(bromomethyl)acrylate (440 mg, 2.0 mmol). Purification by silica-gel column chromatography [cyclohexane/AcOEt, 99:1 to 55:45] yielded the title compound (261 mg, 50%) as a pale-yellow solid. **^{1}H NMR** (400 MHz, $CDCl_3$) δ 6.17 (s, 1H), 5.75–5.70 (m, 2H), 3.97 (dd, *J* = 15.1, 6.4 Hz, 1H), 3.89 (dd, *J* = 15.1, 6.4 Hz, 1H), 3.60 (t, *J* = 6.4 Hz, 1H (N*H*)), 1.50 (s, 6H), 1.21 (s, 6H). **^{13}C NMR** (101 MHz, $CDCl_3$) δ 165.3, 139.8, 125.9, 81.5, 56.0, 47.2, 28.2, 22.7. **HRMS** (ESI): *m/z* calculated for $C_{12}H_{23}NO_3SNa$ $[M+Na]^+$: 284.1291, found 284.1290. **IR**: ν (cm^{-1}) 3205, 1700, 1634, 1149, 1054, 848, 596.

8c

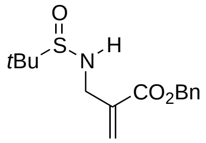

Chemical Formula: $C_{15}H_{21}NO_3S$
Molecular Weight: 295,40

(±)-Benzyl [*N*-*tert*-butanesulfinyl(aminomethyl)acrylate]

Prepared according to **GP1** from (±)-*tert*-butanesulfinamide (**4**, 242 mg, 2.0 mmol) and benzyl 2-(bromomethyl)acrylate (508 mg, 2.0 mmol). Purification by silica-gel column chromatography [cyclohexane/AcOEt, 99:1 to 50:50] yielded the title compound (212 mg, 36%) as a pale-yellow solid. **^{1}H NMR** (400 MHz, $CDCl_3$) δ 7.37–7.30 (m, 5H), 6.33–6.28 (m, 1H), 5.84 (q, *J* = 1.3 Hz, 1H), 5.20 (s, 2H), 4.02 (dd, *J* = 15.2, 6.6 Hz, 1H (AB system)), 3.94 (dd, *J* = 15.3, 6.6 Hz, 1H (AB system)), 3.65 (t, *J* = 6.6 Hz, 1H (N*H*)), 1.16 (s, 9H). **^{13}C NMR** (101 MHz, $CDCl_3$) δ 165.8, 138.2, 135.7, 128.7, 128.4, 128.3, 127.3, 66.8, 56.0, 47.1, 22.6. **HRMS** (ESI): *m/z* calculated for

[1] Murru, S.; Gallo, A. A.; Srivastava, R. S. *J. Org. Chem.*, **2012**, *77*, 7119–7123.

$C_{15}H_{21}NO_3SNa$ [M+Na]$^+$: 318.1134, found 318.1133. **IR**: ν (cm^{-1}) 1720, 1454, 1261, 1153, 1055, 752, 696, 595.

10

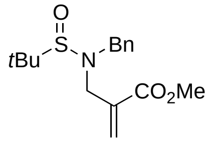

Chemical Formula: $C_{16}H_{23}NO_3S$
Molecular Weight: 309,42

(±)-Methyl [*N*-benzyl-*N*-*tert*-butanesulfinyl(aminomethyl)acrylate]

In a round-bottomed flask under argon, *n*-BuLi (0.48 mL, 2.1 M soln. in heptane, 1.0 mmol) was added dropwise to a THF (5 mL) solution of (±)-*N*-benzyl-*tert*-butanesulfinamide (**9**) [2] (211 mg, 1.0 mmol) at −55 °C. The mixture was then stirred at −10 °C for 30 min and cooled back to −78 °C. Methyl 2-(bromomethyl)acrylate (196 mg, 1.1 mmol) was added and the reaction mixture was stirred for 0.5 h at the same temperature and quenched with aq. 1 M NH_4Cl. The aqueous layer was extracted with Et_2O (× 3) and the combined organics were washed (brine), dried ($MgSO_4$), and concentrated under reduced pressure to provide the crude product. Purification by silica-gel column chromatography [cyclohexane/AcOEt, 90:10] yielded the title compound (304 mg, 96%) as a white solid. **^{1}H NMR** (400 MHz, $CDCl_3$) δ 7.34–7.30 (m, 4H), 7.30–7.24 (m, 1H), 6.33–6.31 (m, 1H), 5.79 (q, *J* = 1.6 Hz, 1H), 4.33 (d, *J* = 15.3 Hz, 1H (AB system)), 4.15 (d, *J* = 15.3 Hz, 1H (AB system)), 4.05 (d, *J* = 17.1 Hz, 1H (AB system)), 3.72 (s, 3H), 3.68 (d, *J* = 17.1 Hz, 1H (AB system)), 1.18 (s, 9H). **^{13}C NMR** (101 MHz, $CDCl_3$) δ 166.7, 136.9, 136.4, 128.8, 128.7, 127.7, 127.6, 77.36, 58.6, 52.1, 23.2, 22.6. **HRMS** (ESI) *m/z* calculated for $C_{16}H_{23}NO_3SNa$ [M+Na]$^+$ 332.1291; found 332.1298.

III. Air-promoted 1,4-addition of dialkylzinc reagents to α-(aminomethyl)acrylates

a. Preparation and characterization of compounds 11, 12, 13, 14a–c, and 15a

General procedure for the air-promoted 1,4-addition of dialkylzinc reagents to α-(aminomethyl)acrylates (GP2): In a Schlenk tube under argon, the appropriate α-(aminomethyl)acrylate (0.2 mmol) was dissolved in the indicated reaction solvent (3 mL) and the solution was cooled to −33 °C. Et_2Zn (1 M in hexanes, 1.0 mL, 1.0 mmol) was added dropwise and the solution was stirred for 1 h. Air (20 mL) was introduced directly into the solution via a syringe fitted with a $CaCl_2$ pad at a 0.5 mL/min^{-1} rate (syringe pump). After the end of the air addition, the mixture was stirred for an additional 80 min at −33 °C and then quenched with aq. NH_4Cl (5 mL) at 0 °C. The aqueous layer was extracted with CH_2Cl_2 (× 2). The combined organics were washed (brine), dried ($MgSO_4$), and concentrated under reduced pressure to provide the crude product which was then purified by column chromatography on silica gel.

11

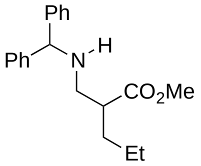

Chemical Formula: $C_{20}H_{25}NO_2$
Molecular Weight: 311,42

Methyl 2-((diphenylmethyl)aminomethyl)pentanoate

Prepared according to **GP2** from α-(aminomethyl)acrylate **5** (56 mg, 0.2 mmol) in CH_2Cl_2. Purification by silica-gel column chromatography [cyclohexane/AcOEt, 99:1 to 90:10] yielded the title compound (26 mg, 42%) as a colorless oil. **^{1}H NMR** (400 MHz, $CDCl_3$) δ 7.40–7.33 (m, 4H), 7.33–7.25 (m, 4H), 7.22–7.16 (m, 2H), 4.80 (s, 1H), 3.70 (s, 3H), 2.82 (dd, *J* = 11.6, 8.6 Hz, 1H), 2.67 (dd, *J* = 11.6, 4.9 Hz, 1H), 2.61 (ddd, *J* = 8.5, 5.3, 3.1 Hz, 1H), 1.66 (br s, 1H (N*H*)), 1.65–1.53 (m, 1H), 1.53–1.39 (m, 1H), 1.35–1.20 (m, 2H), 0.88 (t, *J* = 7.3 Hz, 3H). **^{13}C NMR** (101 MHz, $CDCl_3$) δ 176.1, 144.3, 144.0, 128.572, 128.566, 127.42, 127.39, 127.13, 127.12, 67.4, 51.6, 49.7, 46.2, 32.5, 20.7, 14.1. **HRMS** (ESI): *m/z* calculated for $C_{20}H_{25}NO_2Na$ [M+Na]$^+$: 334.1778, found 334.1769. **IR**: ν (cm^{-1}) 3025, 2956, 1732, 1451, 1192, 698.

---

[2] Brun, S.; Parera, M.; Pla-Quintana, A.; Roglans, A.; Leon, T.; Achard, T.; Sola, J.; Verdaguer, X.; Riera, A. *Tetrahedron*, **2010**, *66*, 9032–9040.

12

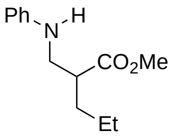

Chemical Formula: $C_{13}H_{19}NO_2$
Molecular Weight: 221,30

Methyl 2-((phenyl)aminomethyl)pentanoate

Prepared according to **GP2** from α-(aminomethyl)acrylate **6** (39 mg, 0.2 mmol) in CH_2Cl_2. Purification by silica-gel column chromatography [cyclohexane/AcOEt, 99:1 to 90:10] yielded the title compound (25 mg, 55%) as a colorless oil. **^{1}H NMR** (400 MHz, $CDCl_3$) δ 7.19 (t, *J* = 7.9 Hz, 2H), 6.75 (t, *J* = 7.3 Hz, 1H), 6.66 (d, *J* = 8.4 Hz, 2H), 3.70 (t, *J* = 7.9 Hz, 1H (N*H*)), 3.69 (s, 3H), 3.40 (dd, *J* = 12.9, 8.6 Hz, 1H), 3.27 (dd, *J* = 12.9, 4.8 Hz, 1H), 2.81–2.72 (m, 1H), 1.75–1.63 (m, 1H), 1.61–1.48 (m, 1H), 1.42–1.30 (m, 2H), 0.93 (t, *J* = 7.3 Hz, 3H). **^{13}C NMR** (101 MHz, $CDCl_3$) δ 175.6, 147.1, 129.5, 118.5, 113.7, 51.9, 46.3, 45.0, 32.4, 20.6, 14.1. **HRMS** (ESI): *m/z* calculated for $C_{13}H_{19}NO_2Na$ $[M+Na]^+$: 244.1308, found 244.1305. **IR**: ν (cm^{-1})2956, 2872, 1729, 1602, 1505, 1195, 1169, 747, 692.

13

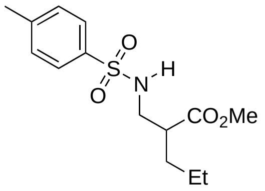

Chemical Formula: $C_{14}H_{21}NO_4S$
Molecular Weight: 299,39

Methyl 2-((tosyl)aminomethyl)pentanoate

Prepared according to **GP2** from α-(aminomethyl)acrylate **7** (54 mg, 0.2 mmol) in CH_2Cl_2. Purification by silica-gel column chromatography [cyclohexane/AcOEt, 99:1 to 80:20] yielded the title compound (33 mg, 55%) as a colorless oil. **^{1}H NMR** (400 MHz, $CDCl_3$) δ 7.73 (d, *J* = 8.3 Hz, 2H), 7.30 (d, *J* = 8.0 Hz, 2H), 5.04 (t, *J* = 6.5 Hz, 1H (N*H*)), 3.64 (s, 3H), 3.07 (t, *J* = 6.5 Hz, 2H), 2.57 (p, *J* = 6.7 Hz, 1H), 2.42 (s, 3H), 1.63–1.51 (m, 1H), 1.45 (ddt, *J* = 13.4, 9.4, 6.7 Hz, 1H), 1.33–1.24 (m, 2H), 0.86 (t, *J* = 7.3 Hz, 3H).**^{13}C NMR** (101 MHz, $CDCl_3$) δ 175.2, 143.5, 137.2, 129.9, 127.2, 52.0, 45.0, 43.9, 31.7, 21.6, 20.2, 13.9. **HRMS** (ESI): *m/z* calculated for $C_{14}H_{21}NO_4SNa$ $[M+Na]^+$: 322.1083, found 322.1085. **IR**: ν (cm^{-1}) 2958, 2873, 1735, 1328, 1157, 1092, 813, 659.

14a

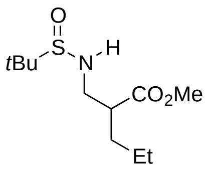

Chemical Formula: $C_{11}H_{23}NO_3S$
Molecular Weight: 249,37

Methyl 2-((*tert*-butanesulfinyl)aminomethyl)pentanoate

Prepared according to **GP2** from α-(aminomethyl)acrylate **8a** (44 mg, 0.2 mmol) in CH_2Cl_2. Purification by silica-gel column chromatography [cyclohexane/AcOEt, 99:1 to 60:40] yielded the title compound as a mixture of diastereomers (38 mg, 76%, 70:30 dr) as a colorless oil. **^{1}H NMR** (400 MHz, $CDCl_3$) δ 3.66 (s, $3H_{minor}$), 3.65 (s, $3H_{major}$), 3.62–3.56 (m, $1H_{major}$ (N*H*)), 3.57–3.52 (m, $1H_{minor}$ (N*H*)), 3.40–3.31 (m, 1H), 3.27–3.17 (m, 1H), 2.67–2.58 (m, 1H), 1.65–1.53 (m, 1H), 1.51–1.40 (m, 1H), 1.36–1.25 (m, 2H), 1.163 (s, $9H_{major}$), 1.158 (s, $9H_{minor}$), 0.89 (t, *J* = 7.4 Hz, $3H_{minor}$), 0.88 (t, *J* = 7.3 Hz, $3H_{major}$). **^{13}C NMR** (101 MHz, $CDCl_3$) (major isomer) δ 175.3, 55.9, 51.8, 47.3, 46.6, 31.9, 22.7, 20.4, 14.0; (minor isomer) δ 175.2, 55.8, 51.7, 46.8, 46.7, 32.0, 22.7, 20.4, 14.0. **HRMS** (ESI): *m/z* calculated for $C_{11}H_{23}NO_3SNa$ $[M+Na]^+$: 272.1291, found 272.1289.

The major isomer has (R_S,S*) relative configuration, as established by analogy with **14b**.*

14b

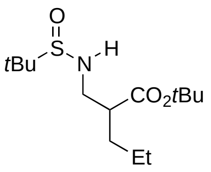

Chemical Formula: $C_{14}H_{29}NO_3S$
Molecular Weight: 291,45

***tert*-Butyl 2-((*tert*-butanesulfinyl)aminomethyl)pentanoate**

Prepared according to **GP2** from α-(aminomethyl)acrylate **8b** (52 mg, 0.2 mmol) in hexane. Purification by silica-gel column chromatography [cyclohexane/AcOEt, 99:1 to 80:20] yielded the title compound as a mixture of diastereomers (51 mg, 88%, 85:15 dr) as white solid. **^{1}H NMR** (400 MHz, $CDCl_3$) δ 3.60–3.53 (m, $1H_{major}$ (N*H*)), 3.55–3.50 (m, $1H_{minor}$ (N*H*)), 3.40–3.28 (m, 1H), 3.25–3.12 (m, 1H), 2.54–2.45 (m, 1H), 1.64–1.51 (m, 1H), 1.51–1.40 (m, 1H), 1.44 (s, 9H), 1.38–1.30 (m, 2H), 1.196 (s, $9H_{major}$), 1.192 (s, $9H_{minor}$), 0.91 (t, *J* = 7.3 Hz, 3H). **^{13}C NMR** (101 MHz, $CDCl_3$) (major isomer) δ 174.2, 81.0, 56.0, 47.3, 47.2, 32.2, 28.3, 22.8, 20.3, 14.1; (minor isomer) δ 174.0, 80.9, 55.8, 47.7, 47.0, 32.2, 29.8, 24.4, 20.4, 14.1.

HRMS (ESI): *m/z* calculated for $C_{14}H_{29}NO_3SNa$ $[M+Na]^+$: 314.1760, found 314.1762.

The major isomer has (R_S,S*) relative configuration, as established by chemical correlation (see below).*

14c

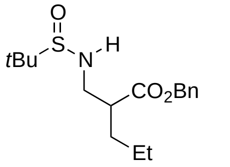

Chemical Formula: $C_{17}H_{27}NO_3S$
Molecular Weight: 325,47

Benzyl 2-((*tert*-butanesulfinyl)aminomethyl)pentanoate

Prepared according to **GP2** from α-(aminomethyl)acrylate **8c** (59 mg, 0.2 mmol) in CH_2Cl_2. Purification by silica-gel column chromatography [cyclohexane/AcOEt, 99:1 to 80:20] yielded the title compound as a mixture of diastereomers (49 mg, 76%, 70:30 dr) as colorless oil. **^{1}H NMR** (400 MHz, $CDCl_3$) δ 7.38–7.30 (m, 5H), 3.55 (dd, *J* = 7.9, 5.9 Hz, $1H_{major}$ (N*H*)), 3.51 (t, *J* = 6.1 Hz, $1H_{minor}$ (N*H*)), 5.13 (s, $2H_{minor}$), 5.11 (s, $2H_{major}$), 3.44–3.34 (m, 1H), 3.31–3.18 (m, 1H), 2.72–2.62 (m, 1H), 1.70–1.56 (m, 1H), 1.55–1.44 (m, 1H), 1.36–1.27 (m, 2H), 1.13 (s, $9H_{major}$), 1.11 (s, $9H_{minor}$), 0.89 (t, *J* = 7.3 Hz, $3H_{minor}$), 0.88 (t, *J* = 7.3 Hz, $3H_{major}$). **^{13}C NMR** (101 MHz, $CDCl_3$) (major isomer) δ 174.6, 135.9, 128.7, 128.4, 128.3, 66.5, 55.9, 47.1, 46.7, 31.8, 22.6, 20.3, 14.0; (minor isomer) δ 174.4, 135.9, 128.7, 128.4, 128.3, 66.5, 55.8, 46.9, 46.8, 32.0, 22.6, 20.4, 14.0. **HRMS** (ESI): *m/z* calculated for $C_{17}H_{27}NO_3SNa$ $[M+Na]^+$: 348.1604, found 348.1611.

The major isomer has (R_S,S*) relative configuration, as established by analogy with **14b**.*

15a

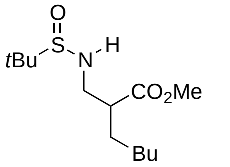

Chemical Formula: $C_{13}H_{27}NO_3S$
Molecular Weight: 277,42

Methyl 2-((*tert*-butanesulfinyl)aminomethyl)heptanoate

Prepared according to **GP2** from α-(aminomethyl)acrylate **8a** (44 mg, 0.2 mmol) in CH_2Cl_2 using *n*-Bu_2Zn instead of Et_2Zn. Purification by silica-gel column chromatography [cyclohexane/AcOEt, 99:1 to 60:40] yielded the title compound as a mixture of diastereomers (40 mg, 71%, 67:33 dr) as colorless oil. **^{1}H NMR** (400 MHz, $CDCl_3$) δ 3.67 (s, $3H_{minor}$), 3.66 (s, $3H_{major}$), 3.56 (dd, *J* = 7.8, 6.1 Hz, $1H_{major}$ (N*H*)), 3.52 (t, *J* = 6.0 Hz, $1H_{minor}$ (N*H*)), 3.41–3.31 (m, 1H), 3.28–3.18 (m, 1H), 2.65–2.56 (m, 1H), 1.67–1.54 (m, 1H), 1.54–1.42 (m, 1H), 1.33–1.22 (m, 6H), 1.17 (s, $9H_{major}$), 1.16 (s, $9H_{minor}$), 0.85 (t, *J* = 6.6 Hz, 3H). **^{13}C NMR** (101 MHz, $CDCl_3$) (major isomer) δ 175.3, 56.0, 51.8, 47.2, 46.8, 31.7, 29.7, 26.8, 22.7, 22.5, 14.1; (minor isomer) δ 175.2, 55.8, 51.8, 46.9, 46.8, 31.7, 29.9, 26.8, 22.7, 22.5, 14.1. **HRMS** (ESI): *m/z* calculated for $C_{13}H_{27}NO_3SNa$ $[M+Na]^+$: 300.1604, found 300.1615.

The major isomer has (R_S,S*) relative configuration, as established by analogy with **14b**.*

b. Chemical correlation to determine the sense of 1,4-stereoinduction in the 1,4-addition reaction

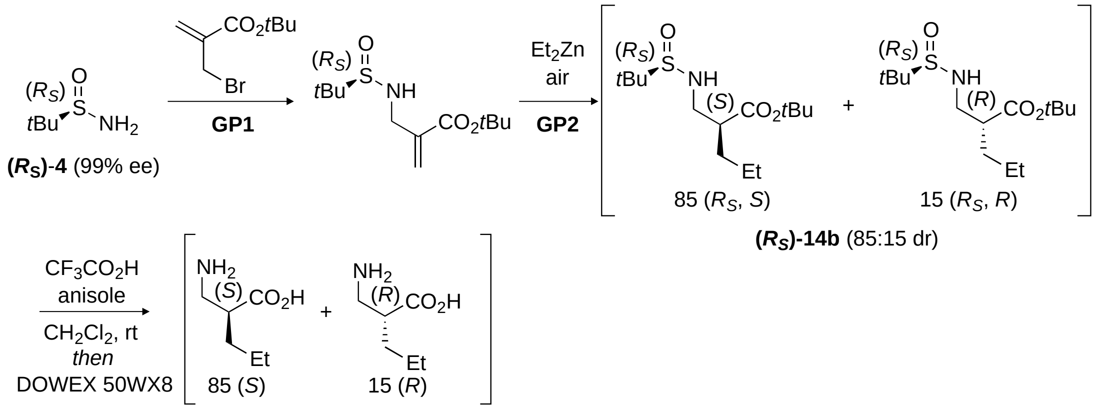

17: 28% (70% ee; *S major isomer*)

Compound (R_S)-**14b** was synthesized as a mixture of diastereomers (85:15 dr) starting from enantiopure (R_S)-*tert*-butanesulfinamide (R_S)-**4** through a two-step sequence involving first reaction with *tert*-butyl 2-(bromomethyl)acrylate according to **GP1** and then reaction with Et_2Zn in hexane according to **GP2**. Concomitant N- and O-deprotection of (R_S)-**14b** leading to β^2-amino acid **17** was performed by treatment with TFA/anisole.

*Procedure for N- and O-deprotection of (R_S)-**14b*** (synthesis of 3-amino-2-propylpropanoic acid (**17**)):

In a round-bottomed flask under argon protection, anisole (0.34 mL, 3.1 mmol) and trifluoroacetic acid (0.33 mL, 4.2 mmol) were added to a CH_2Cl_2 (0.5 mL) solution of (R_S)-**14b** (85:15 dr, 56 mg, 0.2 mmol). The reaction mixture was stirred at rt for 20 h and then concentrated under vacuum. The residue was first purified by column chromatography on silica gel (CH_2Cl_2/MeOH 90:10) and then loaded onto a DOWEX 50WX8 ion-exchange resin conditioned with HCl 1M. Elution with NH_4OH (2M) and evaporation afforded β^2-amino acid **17** as a white solid (7 mg, 28%). **^{1}H NMR** (400 MHz, D_2O) δ 3.13 (dd, *J* = 12.8, 8.5 Hz, 1H), 3.06 (dd, *J* = 12.8, 5.1 Hz, 1H), 2.62–2.50 (m, 1H), 1.66–1.49 (m, 2H), 1.41–1.28 (m, 2H), 0.93 (t, *J* = 7.3 Hz, 3H). **^{13}C NMR** (101 MHz, D_2O) δ 181.3, 45.4, 41.2, 32.1, 19.7, 13.3. The spectral data is in good agreement with that previously reported [3].

Measured optical rotation :		**$[\alpha]^{23}_D$** = –1.9 (c 1.0, HCl 1M).
Reported literature values:	**(S)-17** :	$[\alpha]^{28}_D$ = –3.2 (c 1.0, 1M HCl) [3];
	**(R)-17** :	$[\alpha]^{27}_D$ = +3.5 (c 1.0, 1M HCl) [4]

The comparison of the sign of the $[\alpha]^{23}_D$ with the previous literature reports indicates unambiguously that **17** was obtained in enantioenriched form with the *S* isomer being the major one. Thus, the major isomer of *(R_S)*-**14b** has (R_S,*S*) configuration. The ee calculated from the optical rotation measurements is in reasonably good agreement with the 70% ee expected from the 85:15 dr mixture of the engaged (R_S)-**14b**.

IV. Air-promoted tandem 1,4-addition–aldol reaction between dialkylzinc reagents, α-(aminomethyl)acrylates and carbonyl derivatives (preparation and characterization of compounds 18, 19, 20, 21a, 21b, 22, 23, and 24)

General procedure for the air-promoted tandem 1,4-addition–aldol reaction between dialkylzinc reagents, α-(aminomethyl)-acrylates, and carbonyl derivatives (GP3): In a Schlenk tube under argon, the appropriate α-(aminomethyl)acrylate (0.2 mmol) was dissolved in the indicated reaction solvent (3 mL) and the solution was cooled to −33 °C. The carbonyl electrophile (1.0 mmol) and then Et_2Zn (1 M in hexanes, 1.0 mL, 1.0 mmol) were added dropwise and the solution was stirred for 1 h. Air (20 mL) was introduced directly into the solution via a syringe fitted with a $CaCl_2$ pad at a 0.5 mL/min^{-1} rate (syringe pump). After the end of the air addition, the mixture was stirred for an additional 80 min at −33 °C and then quenched with aq. NH_4Cl (5 mL) at 0 °C. The aqueous layer was extracted with

[3] Gutiérrez-Garcia, V. M.; Reyes-Rangel, G.; Muñoz-Muñiz, O.; Juaristi, E. *Helv. Chim. Acta,* **2002**, *85*, 4189–4199

[4] Nagula, G.; Huber, V. J.; Lum, C.; Goodman, B. A. *Org. Lett.*, **2000**, *2*, 3527–3529.

CH_2Cl_2 (× 2). The combined organics were washed (brine), dried ($MgSO_4$), and concentrated under reduced pressure to provide the crude product which was then purified by column chromatography on silica gel.

18

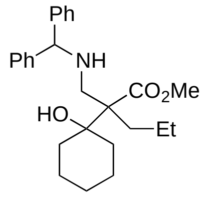

Chemical Formula: $C_{26}H_{35}NO_3$
Molecular Weight: 409,56

Methyl 2-((diphenylmethyl)aminomethyl)-2-(hydroxycyclohexyl)pentanoate

Prepared according to **GP3** from α-(aminomethyl)acrylate **5** (56 mg, 0.2 mmol) and cyclohexanone (0.1 mL, 1.0 mmol) in CH_2Cl_2. Purification by silica-gel column chromatography [cyclohexane/AcOEt, 99:1 to 90:10] yielded the title compound (55 mg, 67%) as colorless oil. **^{1}H NMR** (400 MHz, $CDCl_3$) δ 7.39–7.27 (m, 8H), 7.25–7.19 (m, 2H), 4.69 (s, 1H), 3.68 (s, 3H), 3.24 (d, *J* = 12.8 Hz, 1H), 2.63 (d, *J* = 12.8 Hz, 1H), 1.91 (ddd, *J* = 13.6, 12.4, 3.9 Hz, 1H), 1.82–1.68 (m, 3H), 1.66–1.51 (m, 3H), 1.50–1.32 (m, 3H), 1.30–1.10 (m, 3H), 1.10–0.94 (m, 2H), 0.87 (t, *J* = 7.1 Hz, 3H). **^{13}C NMR** (101 MHz, $CDCl_3$) δ 175.4, 142.9, 142.5, 128.7, 128.6, 127.6, 127.5, 127.4, 127.3, 76.3, 68.2, 57.5, 51.6, 49.5, 33.3, 32.6, 26.0, 21.83, 21.75, 18.8, 15.0. **HRMS** (ESI) *m/z* calculated for $C_{26}H_{35}NO_3Na$ $[M+Na]^+$: 410.2690, found 410.2700. **IR**: ν (cm^{-1}) 2930, 1715, 1450, 1223, 972, 742, 698.

19

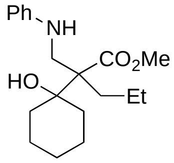

Chemical Formula: $C_{19}H_{29}NO_3$
Molecular Weight: 319,44

Methyl 2-((phenyl)aminomethyl)-2-(hydroxycyclohexyl)pentanoate

Prepared according to **GP3** from α-(aminomethyl)acrylate **6** (39 mg, 0.2 mmol) and cyclohexanone (0.1 mL, 1.0 mmol) in CH_2Cl_2. Purification by silica-gel column chromatography [cyclohexane/AcOEt, 99:1 to 85:15] yielded the title compound (40 mg, 63%) as colorless oil. **^{1}H NMR** (400 MHz, $CDCl_3$) δ 7.26–7.20 (m, 2H), 6.90–6.82 (m, 3H), 3.74 (s, 3H), 3.68 (d, *J* = 7.0 Hz, 1H (N*H*)), 3.58 (d, *J* = 12.8 Hz, 1H), 3.36 (d, *J* = 12.8 Hz, 1H), 2.0–1.9 (m, 2H), 1.80–1.27 (m, 10H), 1.19–1.04 (m, 2H), 0.91 (t, *J* = 7.2 Hz, 3H). **^{13}C NMR** (101 MHz, $CDCl_3$) δ 175.7, 147.0, 129.5, 115.7, 111.5, 76.3, 57.6, 52.0, 47.9, 33.9, 33.0, 32.7, 25.8, 21.9, 21.7, 18.9, 15.0. **HRMS** (ESI) *m/z* calculated for $C_{19}H_{29}NO_3H$ $[M+H]^+$: 320.2220, found 320.2230. **IR**: ν (cm^{-1}) 2954, 2849, 1719, 1601, 1504, 1221, 1120, 972, 747, 691.

20

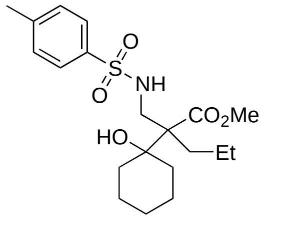

Chemical Formula: $C_{20}H_{31}NO_5S$
Molecular Weight: 397,53

Methyl 2-((tosyl)aminomethyl)-2-(hydroxycyclohexyl)pentanoate

Prepared according to **GP3** from α-(aminomethyl)-acrylate (**7**) (54 mg, 0.2 mmol) and cyclohexanone (0.1 mL, 1.0 mmol) in CH_2Cl_2. Purification by silica-gel column chromatography [cyclohexane/AcOEt, 99:1 to 85:15] yielded the title compound (54 mg, 68%) as colorless oil. **^{1}H NMR** (400 MHz, $CDCl_3$) δ 7.73 (d, *J* = 8.3 Hz, 2H), 7.34 – 7.28 (m, 2H), 5.75 (br s, 1H (N*H*)), 3.64 (s, 3H), 3.23–3.10 (m, 2H), 2.42 (s, 3H), 1.90–1.79 (m, 1H), 1.73 (m, 1H), 1.65–1.40 (m, 8H), 1.35–1.22 (m, 1H), 1.17 (td, *J* = 12.9, 4.5 Hz, 1H), 1.10–0.89 (m, 2H), 0.83 (t, *J* = 7.1 Hz, 3H). **^{13}C NMR** (101 MHz, $CDCl_3$) δ ^{13}C NMR (101 MHz, $CDCl_3$) δ 174.9, 143.4, 136.3, 129.7, 127.1, 75.9, 57.2, 51.9, 44.6, 32.8, 32.7, 32.4, 25.4, 21.6, 21.5, 21.4, 18.3, 14.7. **HRMS** (ESI) *m/z* calculated for $C_{20}H_{31}NO_5SNa$ $[M+Na]^+$: 420.1815, found 420.1799. **IR**: ν (cm^{-1}) 2930, 1727, 1325, 1225, 1162, 1093, 732, 661.

21a

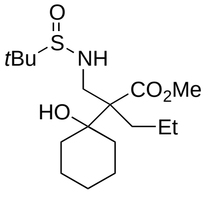

Chemical Formula: $C_{17}H_{33}NO_4S$
Molecular Weight: 347,51

Methyl 2-((*tert*-butanesulfinyl)aminomethyl)-2-(hydroxycyclohexyl)pentanoate

Prepared according to **GP3** from α-(aminomethyl)acrylate **8a** (44 mg, 0.2 mmol) and cyclohexanone (0.1 mL, 1.0 mmol) in CH_2Cl_2. Purification by silica-gel column chromatography [cyclohexane/AcOEt, 99:1 to 60:40] yielded the title compound as a mixture of diastereomers (60 mg, 86%, 75:25 dr) as a white solid. **^{1}H NMR** (400 MHz, $CDCl_3$) δ 4.44 (dd, *J* = 10.6, 4.3 Hz, $1H_{minor}$ (N*H*)), 4.29 (dd, *J* = 8.8, 5.2 Hz, $1H_{major}$ (N*H*)), 3.75 (dd, *J* = 13.4, 4.3 Hz, $1H_{minor}$), 3.682 (s, $3H_{minor}$), 3.679 (s, $3H_{major}$), 3.56 (dd, *J* = 13.2, 5.2 Hz, $1H_{major}$), 3.34 (dd, *J* = 13.2, 8.8 Hz, $1H_{major}$), 3.12 (dd, *J* = 13.4, 10.6 Hz, $1H_{minor}$), 1.97–1.88 (m, $2H_{minor}$), 1.86–1.75 (m, $2H_{major}$), 1.70–0.97 (m, 12H), 1.20 (s, $9H_{minor}$), 1.17 (s, $9H_{major}$), 0.86 (t, *J* = 7.2 Hz, $3H_{major}$), 0.85 (t, *J* = 7.1 Hz, $3H_{minor}$). **^{13}C**

NMR (101 MHz, $CDCl_3$) (major isomer) δ 175.8, 75.5, 58.2, 55.9, 51.8, 48.2, 33.6, 33.1, 32.7, 25.7, 22.8, 21.7, 21.5, 19.2, 15.0; (minor isomer) δ 175.1, 76.0, 58.1, 55.7, 51.8, 47.3, 33.3, 32.9, 32.5, 25.6, 22.8, 21.7, 21.5, 18.5, 14.8. **HRMS** (ESI) *m/z* calculated for $C_{17}H_{33}NO_4SNa$ $[M+Na]^+$: 370.2023, found 370.2037.

21b

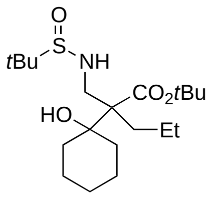

Chemical Formula: $C_{20}H_{39}NO_4S$
Molecular Weight: 389,59

***tert*-Butyl 2-((*tert*-butanesulfinyl)aminomethyl)-2-(hydroxycyclohexyl)pentanoate**

Prepared according to **GP3** from α-(aminomethyl)acrylate **8c** (51 mg, 0.2 mmol) and cyclohexanone (0.1 mL, 1.0 mmol) in hexane. Purification by silica-gel column chromatography [cyclohexane/AcOEt, 99:1 to 60:40] yielded the title compound as a mixture of diastereomers (64 mg, 84%, 90:10 dr) as a white solid. **^{1}H NMR** (400 MHz, $CDCl_3$) δ 4.39 (dd, *J* = 10.8, 3.8 Hz, $1H_{minor}$ (N*H*)), 4.19 (dd, *J* = 9.1, 4.8 Hz, $1H_{major}$ (N*H*)), 3.69 (dd, *J* = 13.3, 3.9 Hz, $1H_{minor}$), 3.52 (dd, *J* = 13.0, 4.8 Hz, $1H_{major}$), 3.23 (dd, *J* = 12.9, 9.4 Hz, $1H_{major}$), 3.04 (dd, *J* = 13.2, 11.0 Hz, $1H_{minor}$), 1.97–1.73 (m, 2H), 1.70–0.97 (m, 12H), 1.44 (s, $9H_{major}$), 1.38 (s, $9H_{minor}$), 1.19 (s, $9H_{minor}$), 1.17 (s, $9H_{major}$), 0.86 (t, *J* = 7.2 Hz, 3H). **^{13}C NMR** (101 MHz, $CDCl_3$) (major isomer) δ 174.6, 81.8, 75.4, 57.9, 55.8, 48.1, 33.6, 32.9, 32.7, 28.2, 25.8, 22.8, 21.8, 21.6, 19.2, 15.1; (minor isomer) δ 173.6, 82.2, 75.9, 57.9, 55.6, 47.2, 33.5, 33.1, 32.8, 27.0, 25.7, 22.9, 21.8, 21.6, 18.3, 14.9. **HRMS** (ESI) *m/z* calculated for $C_{20}H_{39}NO_4SH$ $[M+H]^+$: 390.2673, found 390.2676.

22

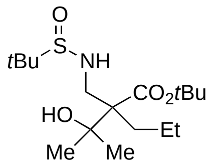

Chemical Formula: $C_{17}H_{35}NO_4S$
Molecular Weight: 349,53

***tert*-Butyl 2-((*tert*-butanesulfinyl)aminomethyl)-2-(1-hydroxy-1-methylethyl)pentanoate**

Prepared according to **GP3** from α-(aminomethyl)acrylate **8c** (51 mg, 0.2 mmol) and acetone (72 μL, 1.0 mmol) in hexane. Purification by silica-gel column chromatography [cyclohexane/AcOEt, 99:1 to 60:40] yielded the title compound as a mixture of diastereomers (53 mg, 77%, 95:5 dr) as a white solid. **^{1}H NMR** (400 MHz, $CDCl_3$) (major isomer) δ 4.25 (dd, *J* = 9.3, 4.8 Hz, 1H (N*H*)), 3.52 (dd, *J* = 13.0, 4.9 Hz, 1H), 3.21 (dd, *J* = 13.0, 9.4 Hz, 1H), 1.80 (td, *J* = 13.1, 4.2 Hz, 1H), 1.58–1.32 (m, 3H), 1.45 (s, 9H), 1.24 (s, 3H), 1.23 (s, 3H), 1.18 (s, 9H), 0.86 (t, *J* = 7.2 Hz, 3H). **^{13}C NMR** (101 MHz, $CDCl_3$) δ ^{13}C NMR (75 MHz, $CDCl_3$) δ 174.6, 82.4, 74.6, 57.3, 55.9, 48.8, 34.2, 28.2, 26.8, 26.6, 22.8, 19.2, 15.1. **HRMS** (ESI) *m/z* calculated for $C_{17}H_{35}NO_4SH$ $[M+H]^+$: 350.2360, found 350.2362.

23

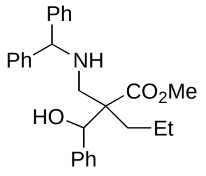

Chemical Formula: $C_{27}H_{31}NO_3$
Molecular Weight: 417,54

Methyl 2-((diphenylmethyl)aminomethyl)-2-(hydroxy(phenyl)methyl)pentanoate

Prepared according to **GP3** from α-(aminomethyl)acrylate **5** (56 mg, 0.2 mmol) and benzaldehyde (0.1 mL, 1.0 mmol) in CH_2Cl_2. Purification by silica-gel column chromatography [cyclohexane/AcOEt, 99:1 to 90:10] yielded the title compound as a mixture of diastereomers (75 mg, 89%, 62:38 dr) as a white solid. **^{1}H NMR** (400 MHz, $CDCl_3$) δ 7.35–7.23 (m, 8H), 7.22–7.06 (m, 7H), 5.08 (s, $1H_{major}$), 4.82 (s, $1H_{minor}$), 4.73 (s, $1H_{minor}$), 4.69 (s, $1H_{major}$), 3.73 (s, $3H_{major}$), 3.48 (s, $3H_{minor}$), 3.16 (d, *J* = 12.6 Hz, $1H_{minor}$), 3.00 (d, *J* = 12.3 Hz, $1H_{major}$), 2.61 (d, *J* = 12.6 Hz, $1H_{minor}$), 2.31 (d, *J* = 12.3 Hz, $1H_{major}$), 1.67–1.50 (m, $1H_{major}$), 1.44 (td, *J* = 12.6, 4.9 Hz, $1H_{major}$), 1.25–1.08 (m, $1H_{minor}$), 1.05–0.87 (m, $2H_{major}$ + $1H_{minor}$), 0.86–0.65 (m, $2H_{minor}$), 0.76 (t, *J* = 7.3 Hz, $3H_{minor}$), 0.68 (t, *J* = 7.2 Hz, $3H_{major}$). **^{13}C NMR** (101 MHz, $CDCl_3$) δ ^{13}C NMR (101 MHz, $CDCl_3$) δ 176.0 (major), 174.4 (minor), 142.9 (major), 142.8 (minor), 142.6 (minor), 142.0 (major), 141.0 (major), 140.8 (minor), 128.9, 128.8, 128.7, 127.90, 127.86, 127.7, 127.6, 127.54, 127.49, 127.44, 127.38, 127.31, 127.27, 81.4 (minor), 81.0 (major), 68.1 (minor), 67.7 (major), 55.1 (major), 54.9 (minor), 52.2 (major), 51.3 (minor), 51.1 (minor), 49.6 (major), 37.1 (major), 36.1 (minor), 18.1, 14.6 (minor), 14.5 (major). **HRMS** (ESI) *m/z* calculated for $C_{27}H_{31}NO_3Na$ $[M+Na]^+$: 418.2377, found 418.2390.

24

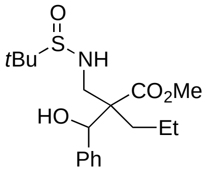

Chemical Formula: $C_{18}H_{29}NO_4S$
Molecular Weight: 355,49

Methyl 2-((*tert*-butanesulfinyl)aminomethyl)-2-(hydroxy(phenyl)methyl)pentanoate

Prepared according to **GP3** from α-(aminomethyl)acrylate **8a** (44 mg, 0.2 mmol) and benzaldehyde (0.1 mL, 1.0 mmol) in CH_2Cl_2. Purification by silica-gel column chromatography [cyclohexane/AcOEt, 99:1 to 60:40] yielded the title compound as a mixture of diastereomers (55 mg, 77%, 29:28:28:15 dr) as a white solid. Given the complexity of the mixture it was not possible to obtain unambiguous **^{1}H NMR** characterization data for each isomer. Specific relevant signals could nevertheless by used to calculate the diastereomeric ratio of products. **^{13}C NMR** (101 MHz, $CDCl_3$) ^{13}C NMR (101 MHz, $CDCl_3$) δ 176.2, 175.3, 174.6, 174.3, 140.7, 140.2, 140.0, 139.0, 128.34, 128.30, 128.24, 128.19, 128.15, 128.13, 128.0, 127.4, 127.2, 78.9, 78.5, 77.27, 76.2, 56.1, 56.02, 56.00, 55.9, 55.84, 55.80, 55.5, 52.3, 52.1, 51.8, 51.7, 48.8, 47.9, 46.3, 45.1, 35.1, 35.0, 34.8, 33.8, 32.0, 31.5, 30.3, 29.8, 29.7, 29.4, 22.82, 22.78, 22.75, 18.1, 17.92, 17.87, 17.8, 14.7, 14.60, 14.55, 14.2. **HRMS** (ESI) *m/z* calculated for $C_{18}H_{29}NO_4SNa$ $[M+Na]^+$: 378.1710, found 378.1722.

V. Diagnostic Experiments

a. I-atom transfer experiment (preparation and characterization of 25a)

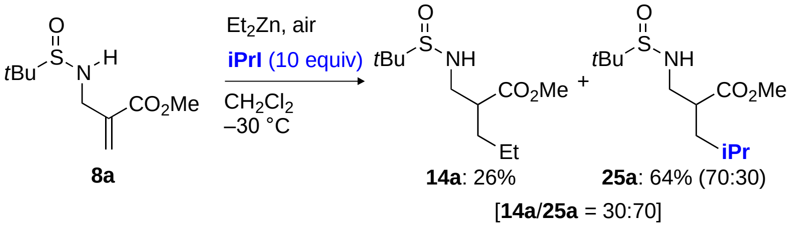

In a Schlenk tube under argon, α-(aminomethyl)acrylate **8a** (0.2 mmol) was dissolved in CH_2Cl_2 (3 mL) and the solution was cooled to −33 °C. 2-Iodopropane (1 mL, 1.0 mmol) and then Et_2Zn (1 M in hexanes, 1.0 mL, 1.0 mmol) were added dropwise and the solution was stirred for 1 h. Air (20 mL) was introduced directly into the solution via a syringe fitted with a $CaCl_2$ pad at a 0.5 mL/min^{-1} rate (syringe pump). After the end of the air addition, the mixture was stirred for an additional 80 min at −33 °C and then quenched with aq. NH_4Cl (5 mL) at 0 °C. The aqueous layer was extracted with CH_2Cl_2 (× 2). The combined organics were washed (brine), dried ($MgSO_4$) and concentrated under reduced pressure to provide the crude product containing a mixture of **14a** and **25a** (**14a**/**25a** = 30:70). Purification by column chromatography on silica gel [cyclohexane/AcOEt, 90:10 to 40:60] afforded **14a** (13 mg, 26% yield) and **25a** (34 mg, 64%, 70:30 dr).

25a

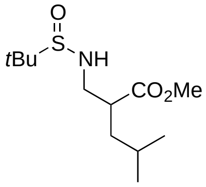

Chemical Formula: $C_{12}H_{25}NO_3S$
Molecular Weight: 263,40

Methyl 2-((*tert*-butanesulfinyl)aminomethyl)-4-methylpentanoate

(70:30 mixture of diastereomers): **^{1}H NMR** (400 MHz, $CDCl_3$) δ 3.68 (s, $3H_{minor}$), 3.67 (s, $3H_{major}$), 3.59–3.53 (m, $1H_{major}$ (N*H*)), 3.53–3.48 (m, $1H_{minor}$ (N*H*)), 3.40–3.29 (m, 1H), 3.27–3.17 (m, 1H), 2.74–2.66 (m, 1H), 1.62–1.48 (m, 2H), 1.38–1.26 (m, 1H), 1.184 (s, $9H_{major}$), 1.179 (s, $9H_{minor}$), 0.92–0.86 (m, 6H). **^{13}C NMR** (101 MHz, $CDCl_3$) (major isomer) δ 175.6, 56.0, 51.9, 47.6, 45.0, 38.8, 29.8, 26.0, 22.7, 22.6, 22.4; (minor isomer) δ 175.5, 55.8, 51.8, 47.2, 45.1, 39.1, 26.1, 22.8, 22.7, 22.4. **HRMS** (ESI): *m/z* calculated for $C_{12}H_{25}NO_3SNa$ $[M+Na]^+$: 286.1447, found 286.1452.

The major isomer has (R_S,S*) relative configuration, as established by analogy with **14b**.*

b. D-labeling experiments

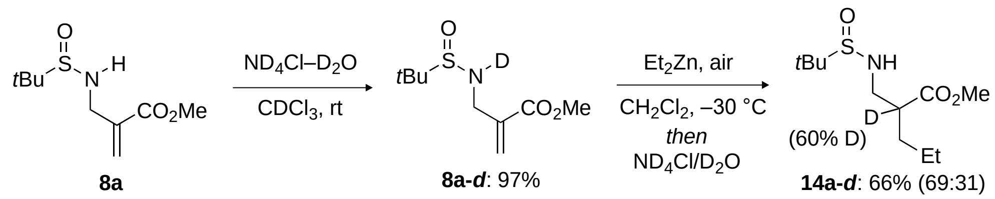

In a round-bottomed flask under Ar, α-(aminomethyl)acrylate **8a** (0.2 mmol) was dissolved in $CDCl_3$ (2 mL) and a D_2O (2 mL) solution of ND_4Cl (140 mg, 2.5 mmol) was added. The biphasic mixture was stirred vigorously for 2 h and then the organic layer was separated, dried ($MgSO_4$) and concentrated to provide **8a-*d*** (43 mg, 97%). α-(Aminomethyl)acrylate **8a-*d*** (0.2 mmol) was then engaged following general procedure **GP2** using ND_4Cl–D_2O for the quench. Work-up and purification of the crude product by flash chromatography on silica gel provided **14a-*d*** as a mixture of diastereomers (33 mg, 66%, 69:31 dr, 60% D-incorporation).

c. Air-promoted tandem 1,4-addition–aldol condensation between Et_2Zn, *N*-benzyl α-(aminomethyl)acrylate 10, and benzaldehyde

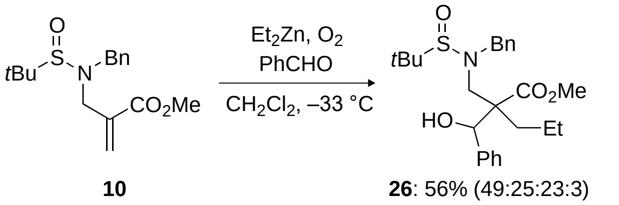

α-(Aminomethyl)acrylate **10** (0.46 mmol) was engaged following general procedure **GP3** using O_2 instead of air. Purification by chromatography on silica gel [cyclohexane/AcOEt, 90:10 to 40:60] afforded adduct **26** as a mixture of diastereomers (116 mg, 56%, 49:25:23:3 dr).

26

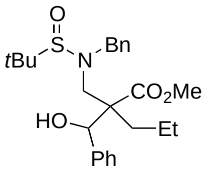

Chemical Formula: $C_{25}H_{35}NO_4S$
Molecular Weight: 445,61

Methyl 2-[*N*-benzyl-*N*-*tert*-butanesulfinyl(aminomethyl)]-2-(hydroxy(phenyl)methyl)pentanoate

(49:25:23:3 mixture of diastereomers): Given the complexity of the mixture it was not possible to obtain unambiguous **^{1}H NMR** characterization data for each isomer. Specific relevant signals could nevertheless by used to calculate the diastereomeric ratio of products. **^{13}C NMR** (101 MHz, $CDCl_3$) δ 176.3, 175.3, 175.1, 174.2, 140.6, 140.31, 140.26, 140.2, 137.7, 137.2, 136.94, 136.89, 129.2, 129.1, 128.9, 128.8, 128.7, 128.6, 128.5, 128.4, 128.3, 128.2, 128.0, 127.84, 127.79, 127.73, 127.65, 127.58, 127.55, 127.5, 127.36, 127.33, 127.28, 127.14, 127.12, 76.0, 75.2, 74.7, 74.0, 60.8, 60.0, 59.7, 59.16, 59.13, 56.7, 56.0, 55.8, 55.7, 53.8, 53.5, 52.58, 51.57, 51.43, 51.36, 51.3, 50.8, 50.5, 50.5, 50.3, 50.2, 34.3, 32.9, 32.6, 32.2, 24.5, 24.4, 24.3, 17.7, 17.5, 17.3, 17.0, 14.8, 14.6, 14.5, 14.2.

VI. ^{1}H NMR and ^{13}C NMR spectra of new compounds

Methyl [*N*-diphenylmethyl(aminomethyl)acrylate] (5) – ^{1}H NMR (400 MHz, $CDCl_3$)

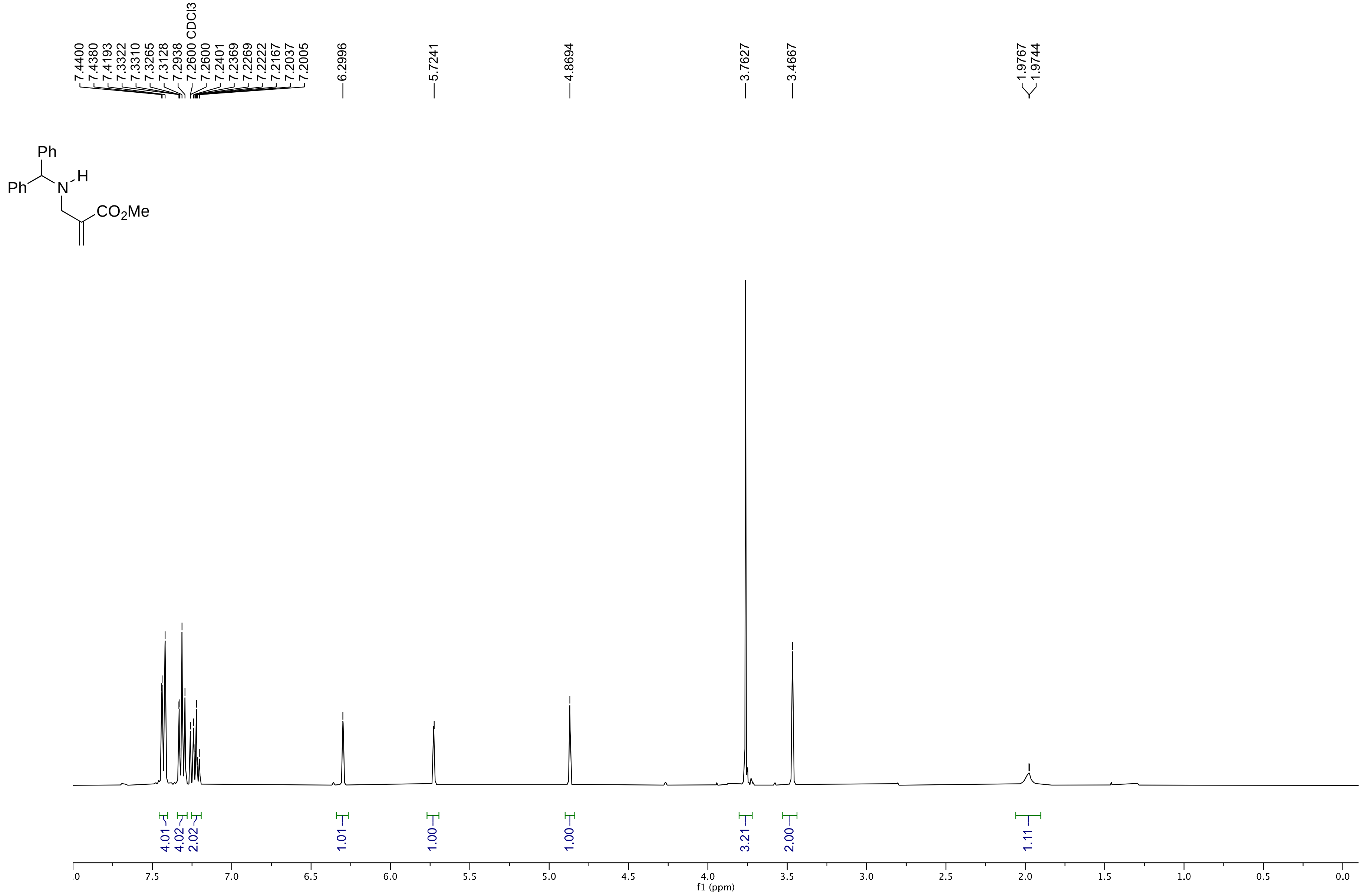

Methyl [*N*-diphenylmethyl(aminomethyl)acrylate] (5) – ^{13}C NMR (100 MHz, $CDCl_3$)

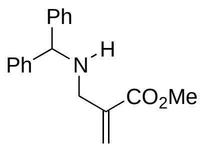

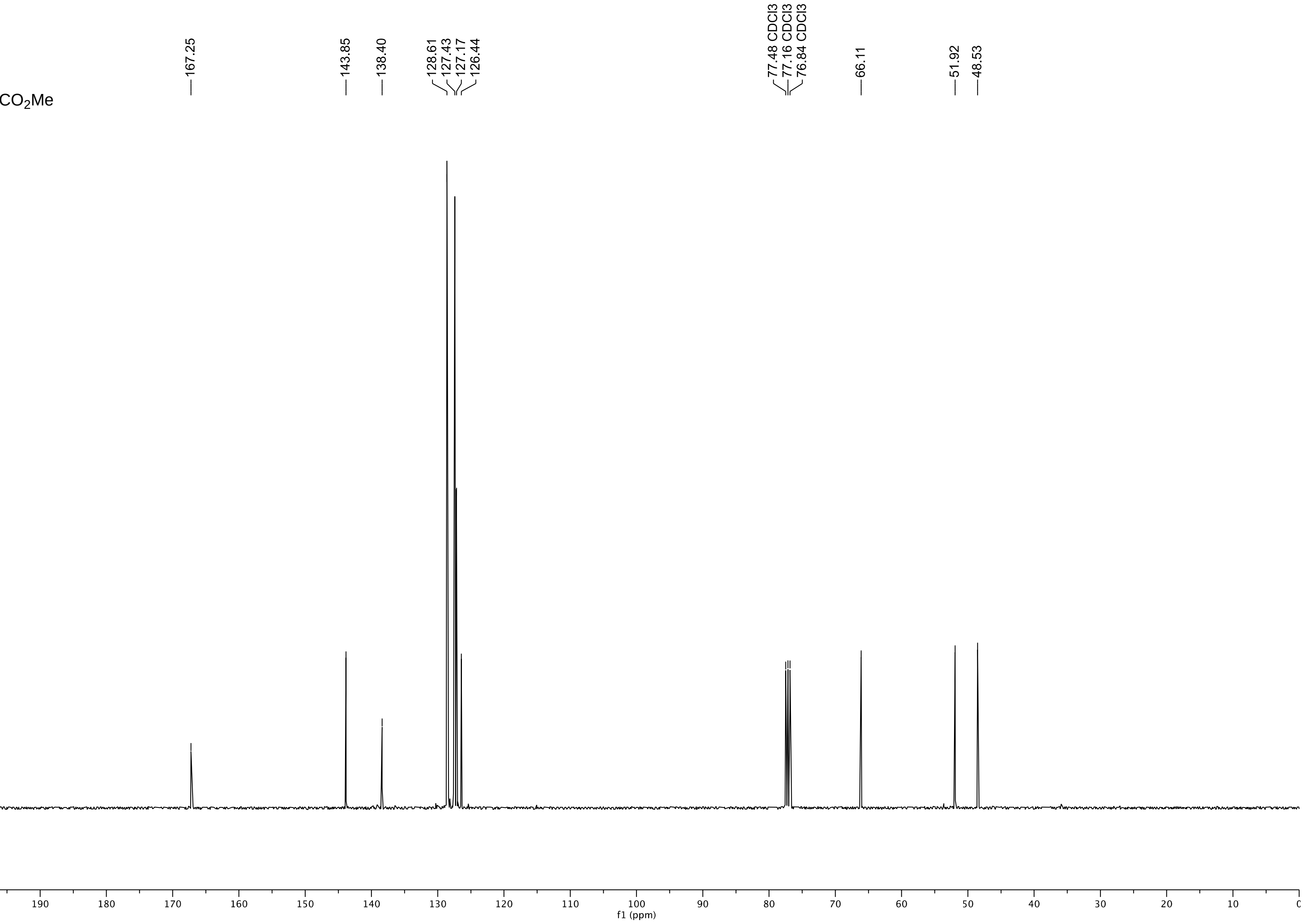

Methyl [*N*-tosyl(aminomethyl)acrylate] (7) – ^{1}H NMR (400 MHz, $CDCl_3$)

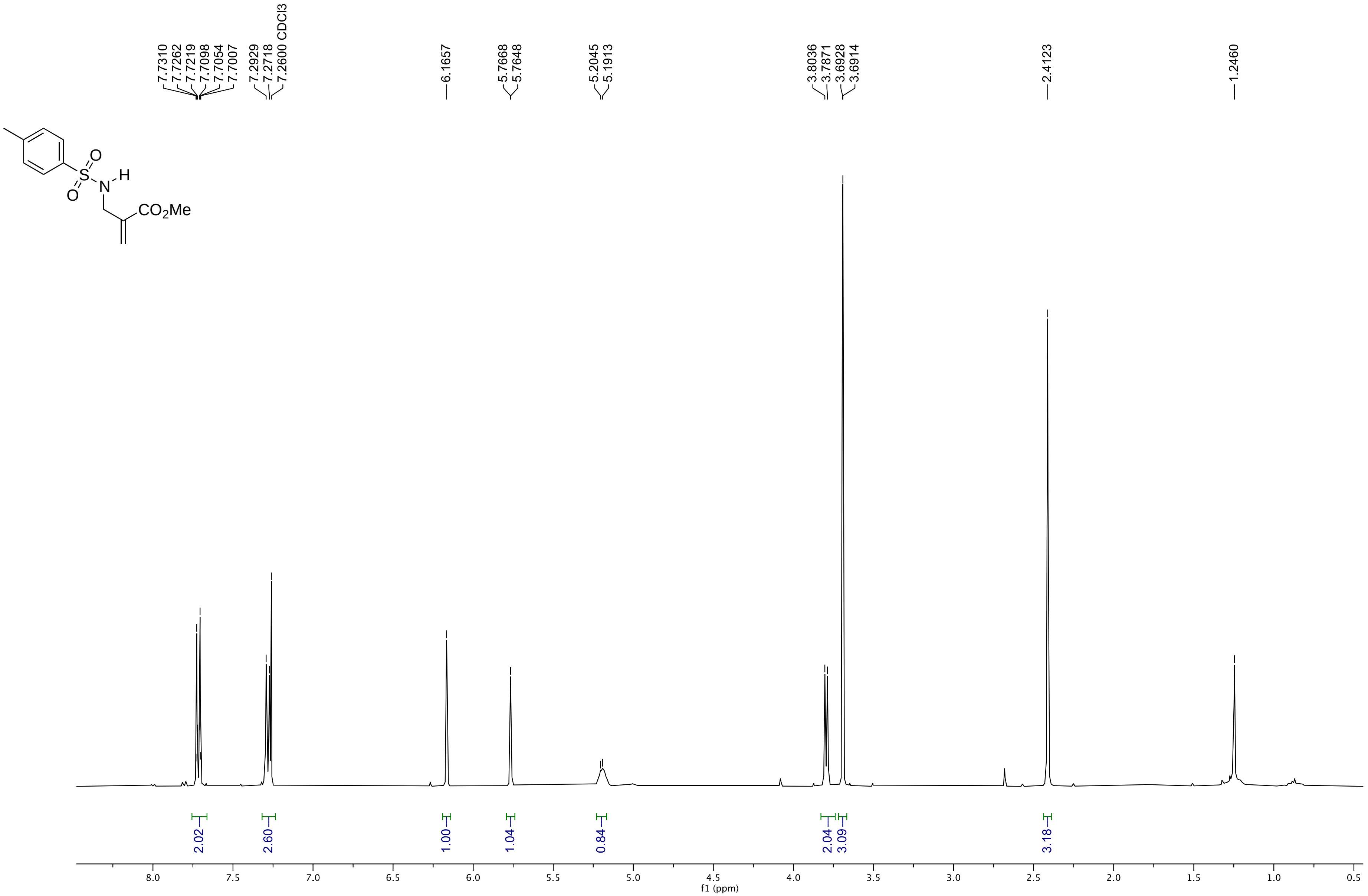

Methyl [*N*-tosyl(aminomethyl)acrylate] (7) – ^{13}C NMR (100 MHz, $CDCl_3$)

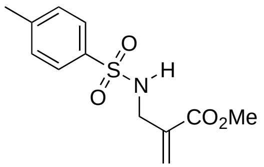

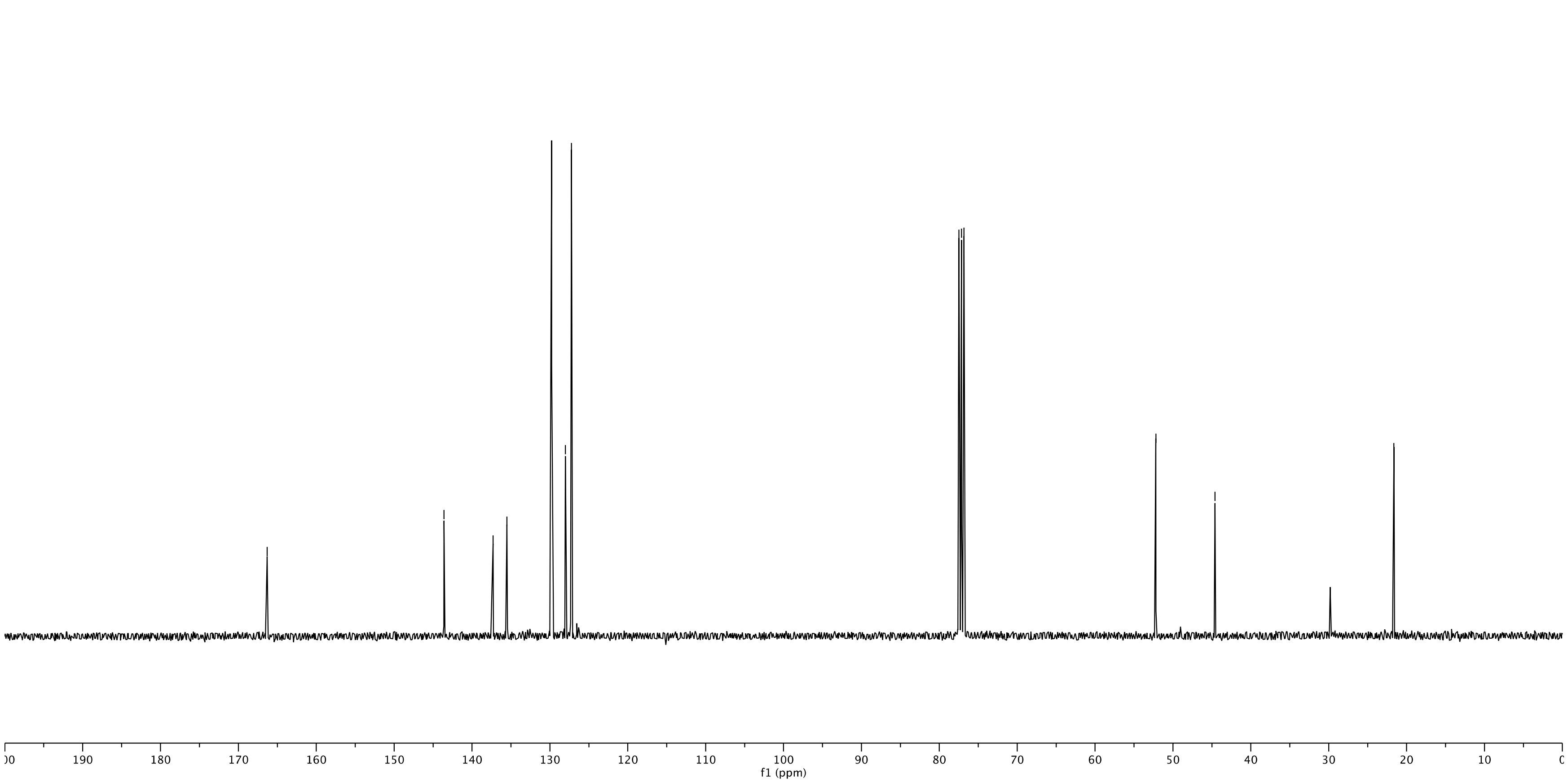

(±)-Methyl [*N*-*tert*-butanesulfinyl(aminomethyl)acrylate] (8a) – ^{1}H NMR (400 MHz, $CDCl_3$)

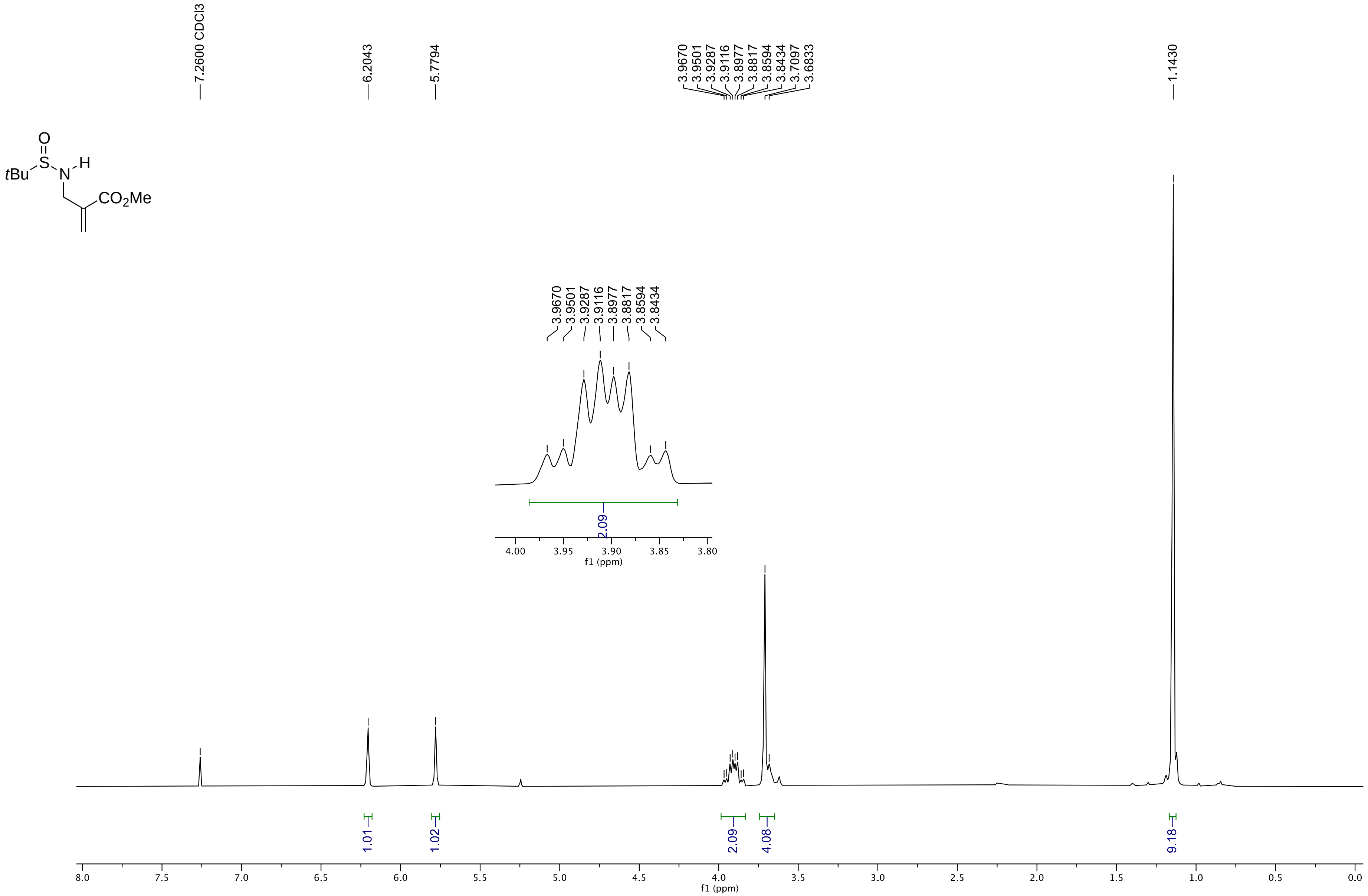

(±)-Methyl [*N*-*tert*-butanesulfinyl(aminomethyl)acrylate] (8a) – ^{13}C NMR (100 MHz, $CDCl_3$)

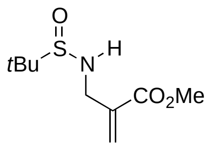

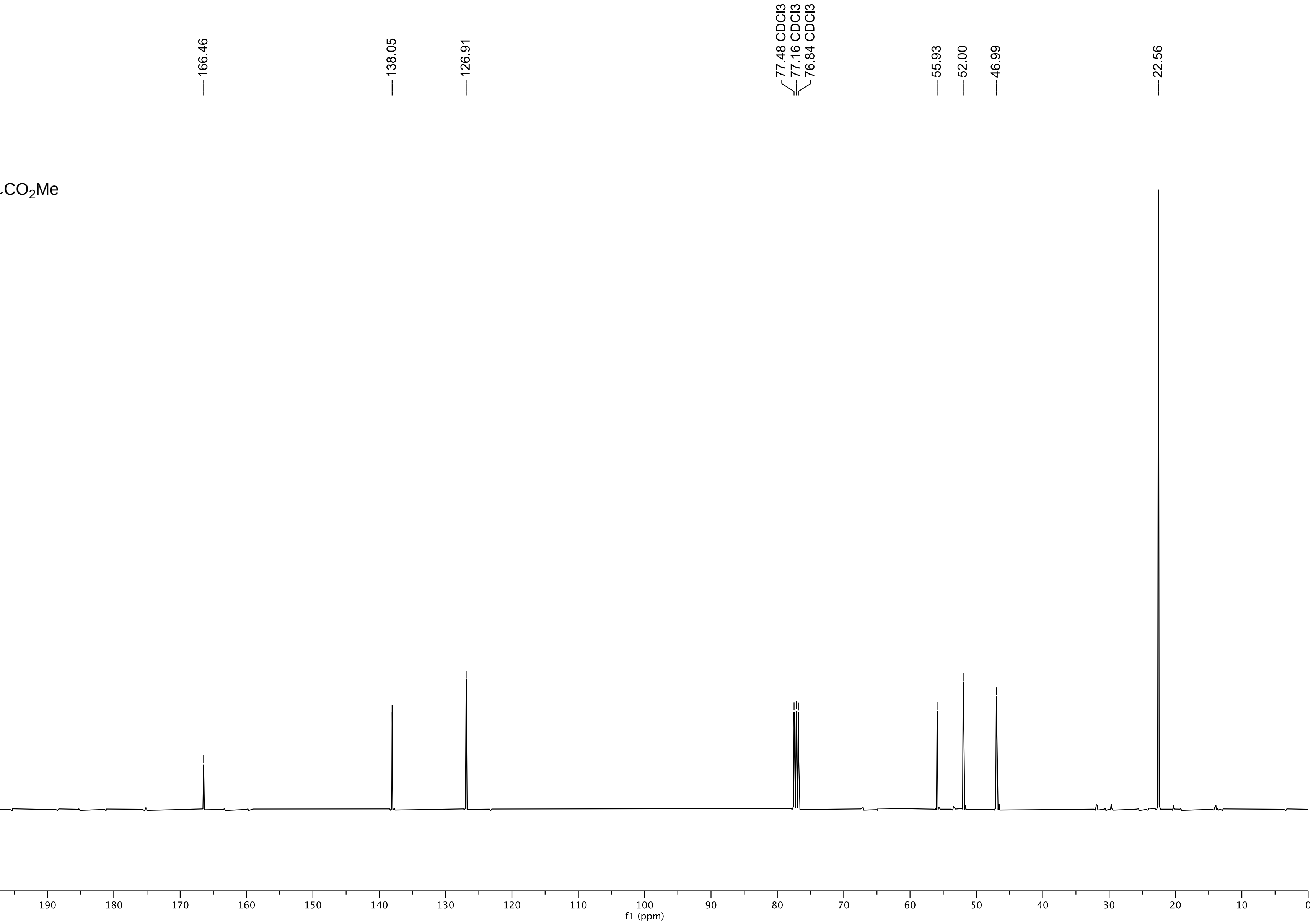

Deuterated (±)-methyl [*N-tert*-butanesulfinyl(aminomethyl)acrylate] (8a-*d*) – ^{1}H NMR (400 MHz, $CDCl_3$)

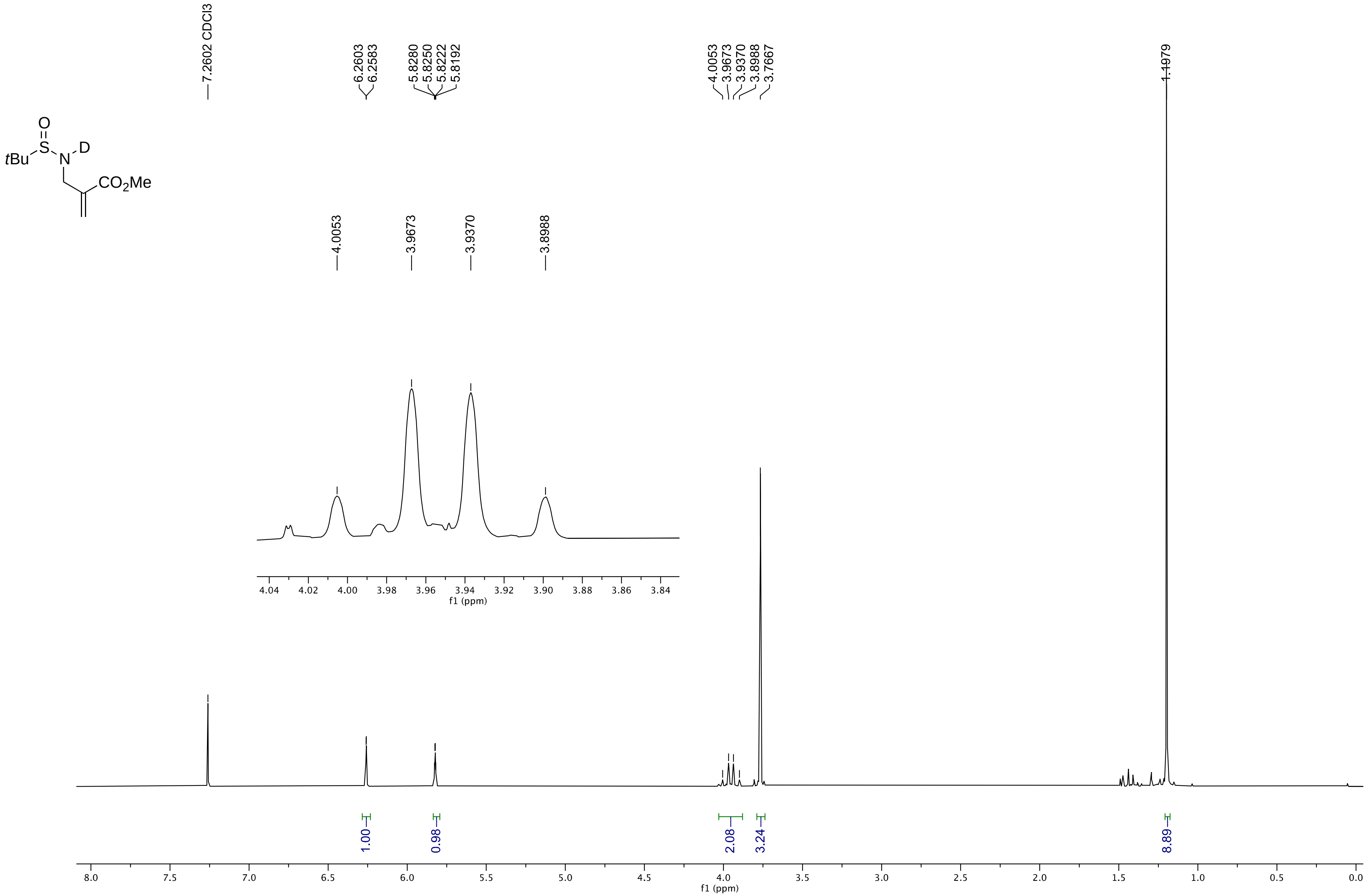

(±)-*tert*-Butyl [*N*-*tert*-butanesulfinyl(aminomethyl)acrylate] (8b) – ^{1}H NMR (400 MHz, $CDCl_3$)

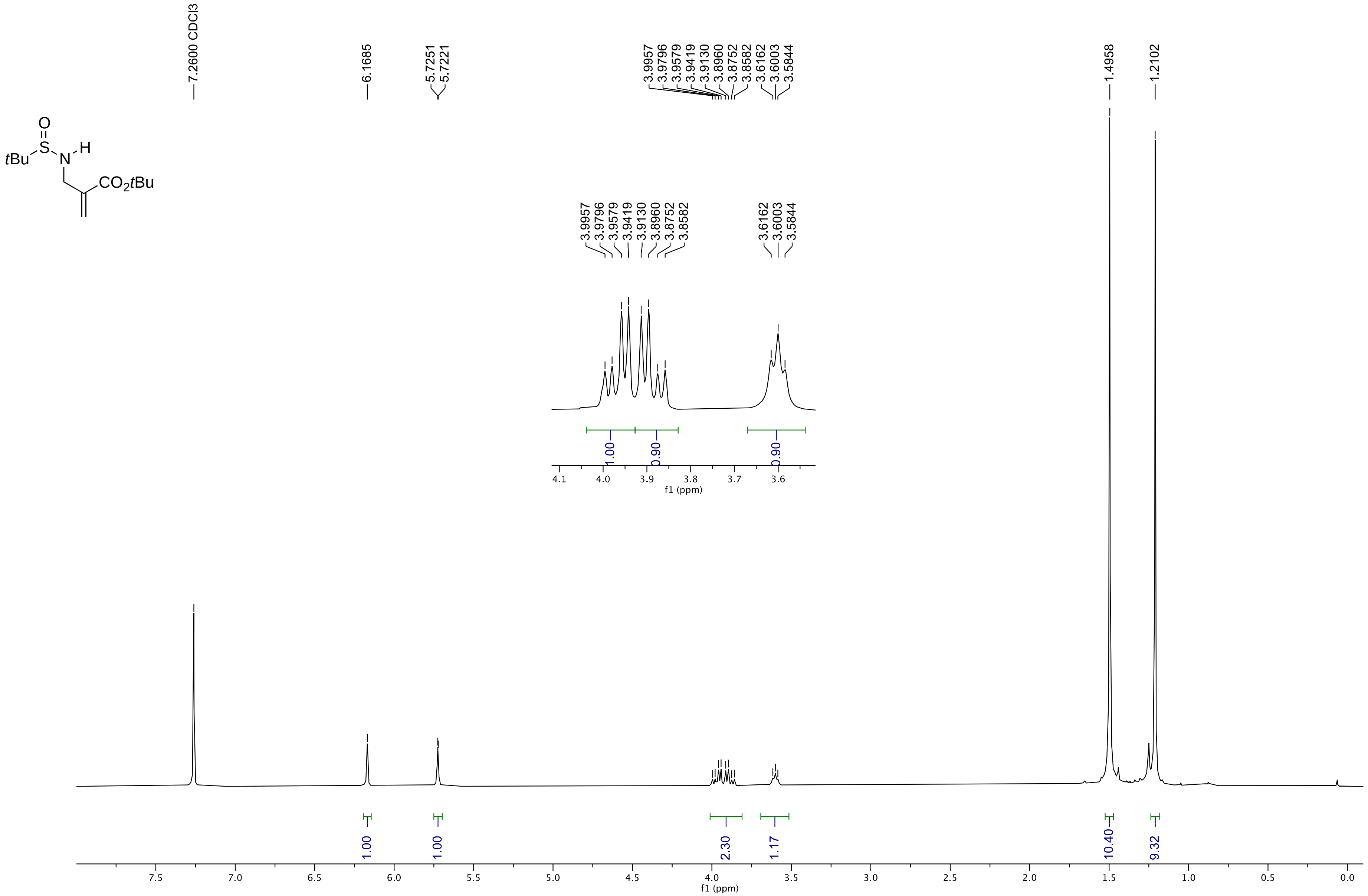

(±)-*tert*-Butyl [*N*-*tert*-butanesulfinyl(aminomethyl)acrylate] (8b) – ^{13}C NMR (100 MHz, $CDCl_3$)

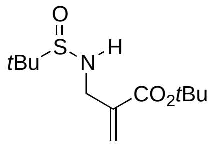

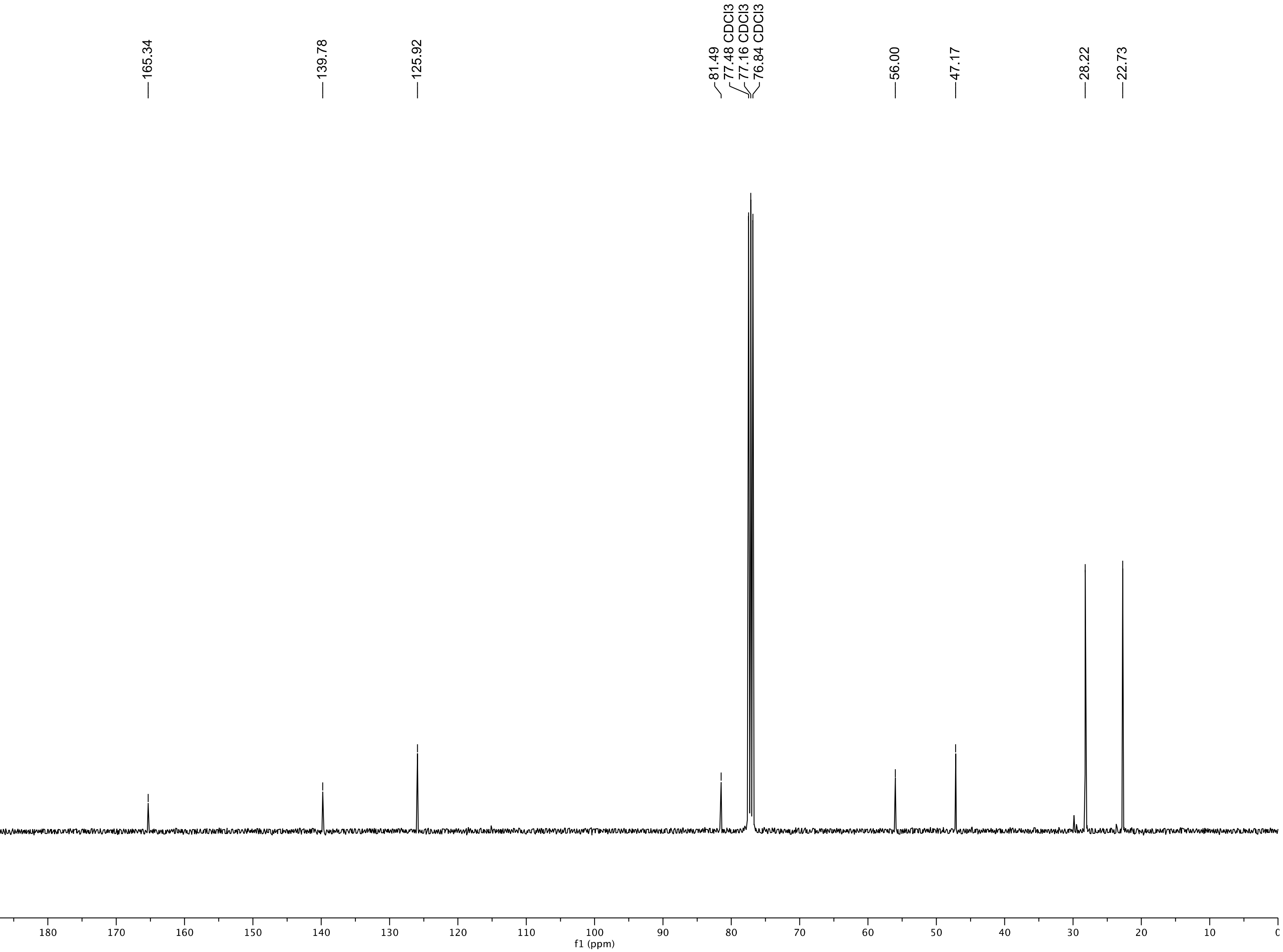

(±)-Benzyl [*N-tert*-butanesulfinyl(aminomethyl)acrylate] (8c) – ^{1}H NMR (400 MHz, $CDCl_3$)

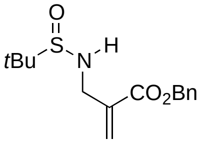

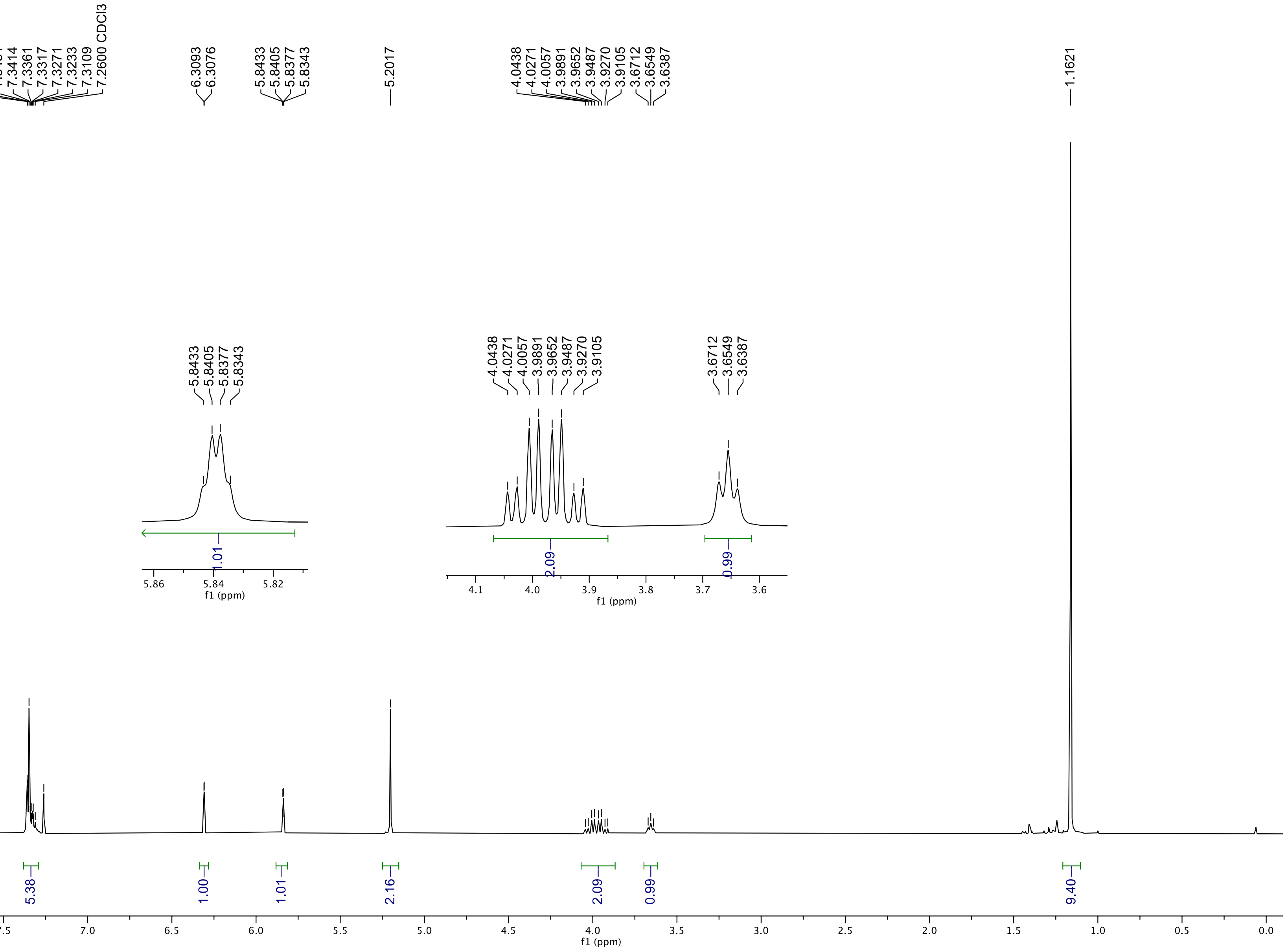

(±)-Benzyl [*N*-*tert*-butanesulfinyl(aminomethyl)acrylate] (8c) – ^{13}C NMR (100 MHz, $CDCl_3$)

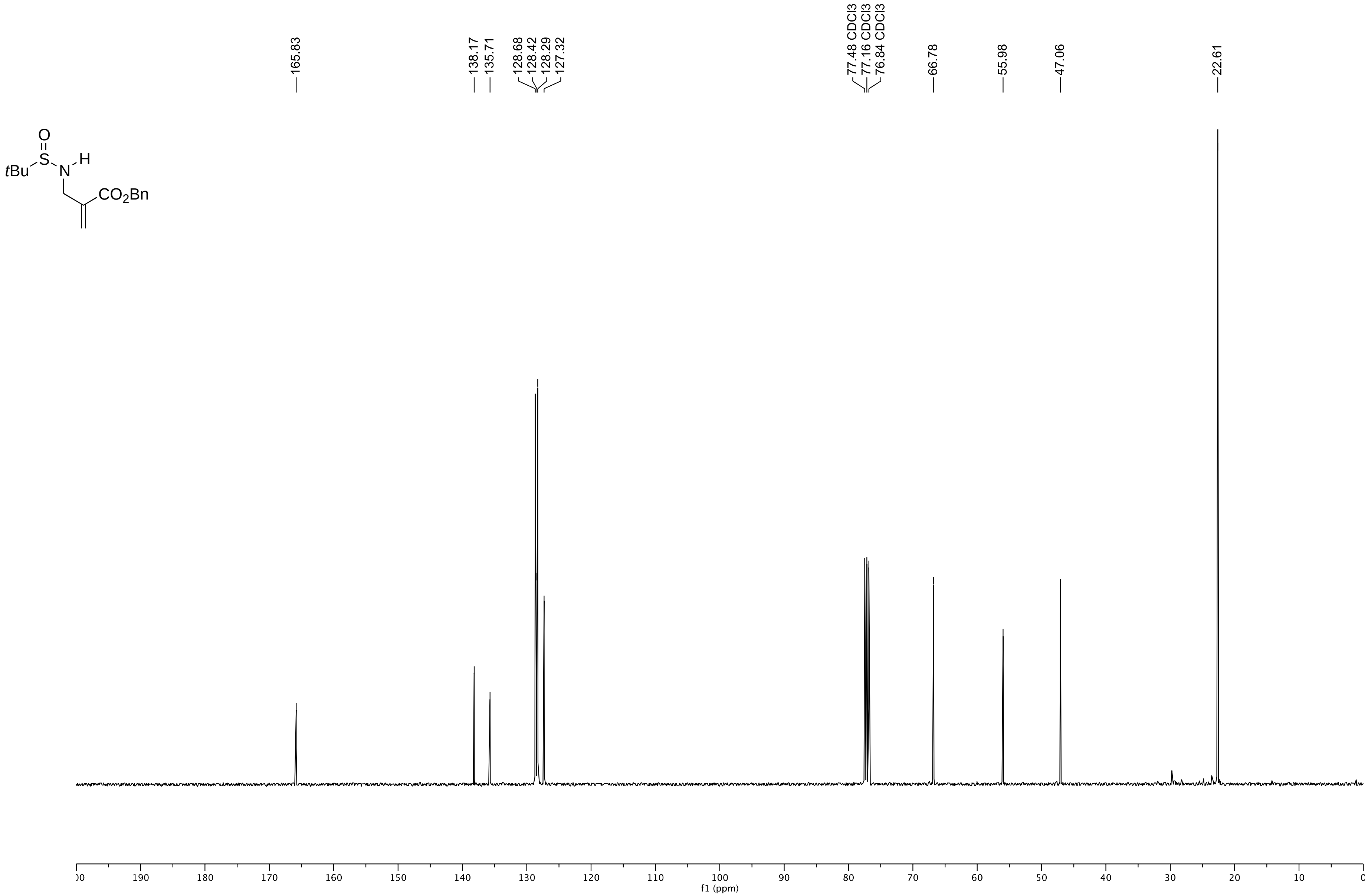

(±)-Methyl [*N*-benzyl-*N*-*tert*-butanesulfinyl(aminomethyl)acrylate] (10) – ^{1}H NMR (400 MHz, $CDCl_3$)

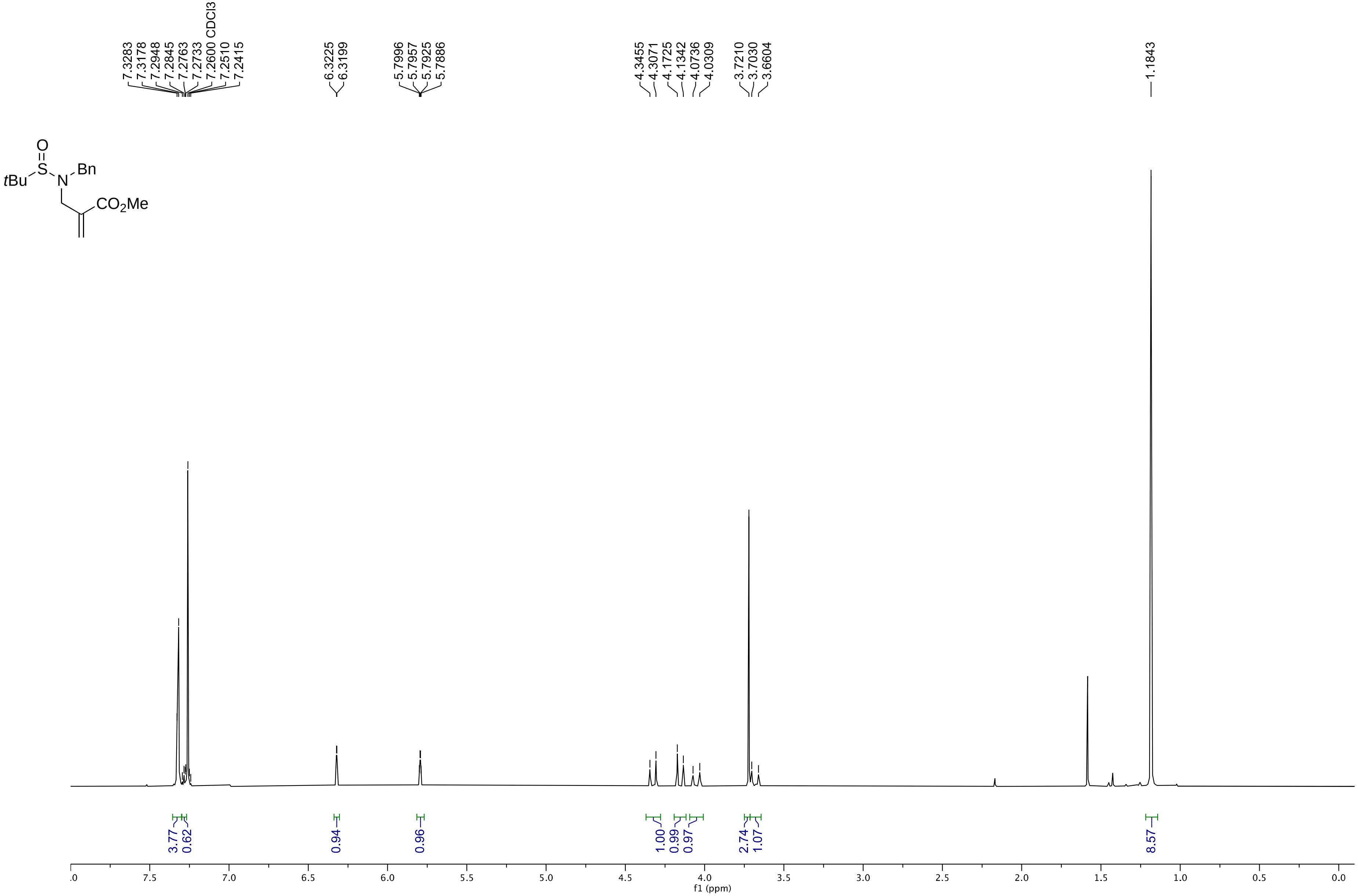

(±)-Methyl [*N*-benzyl-N-*tert*-butanesulfinyl(aminomethyl)acrylate] (10) – ^{13}C NMR (100 MHz, $CDCl_3$)

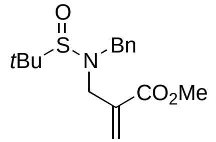

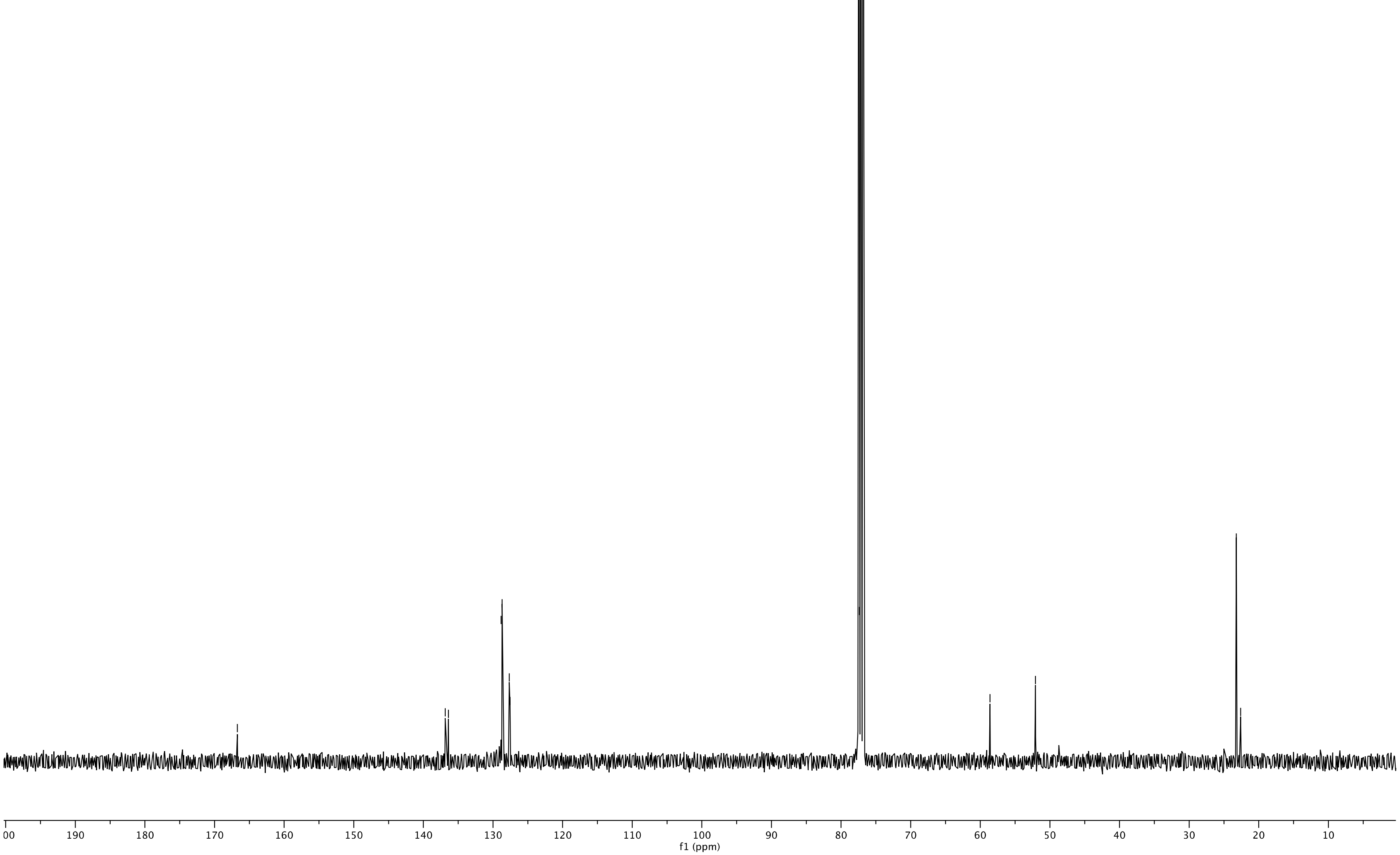

Methyl 2-((diphenylmethyl)aminomethyl)pentanoate (11) – ^{1}H NMR (400 MHz, $CDCl_3$)

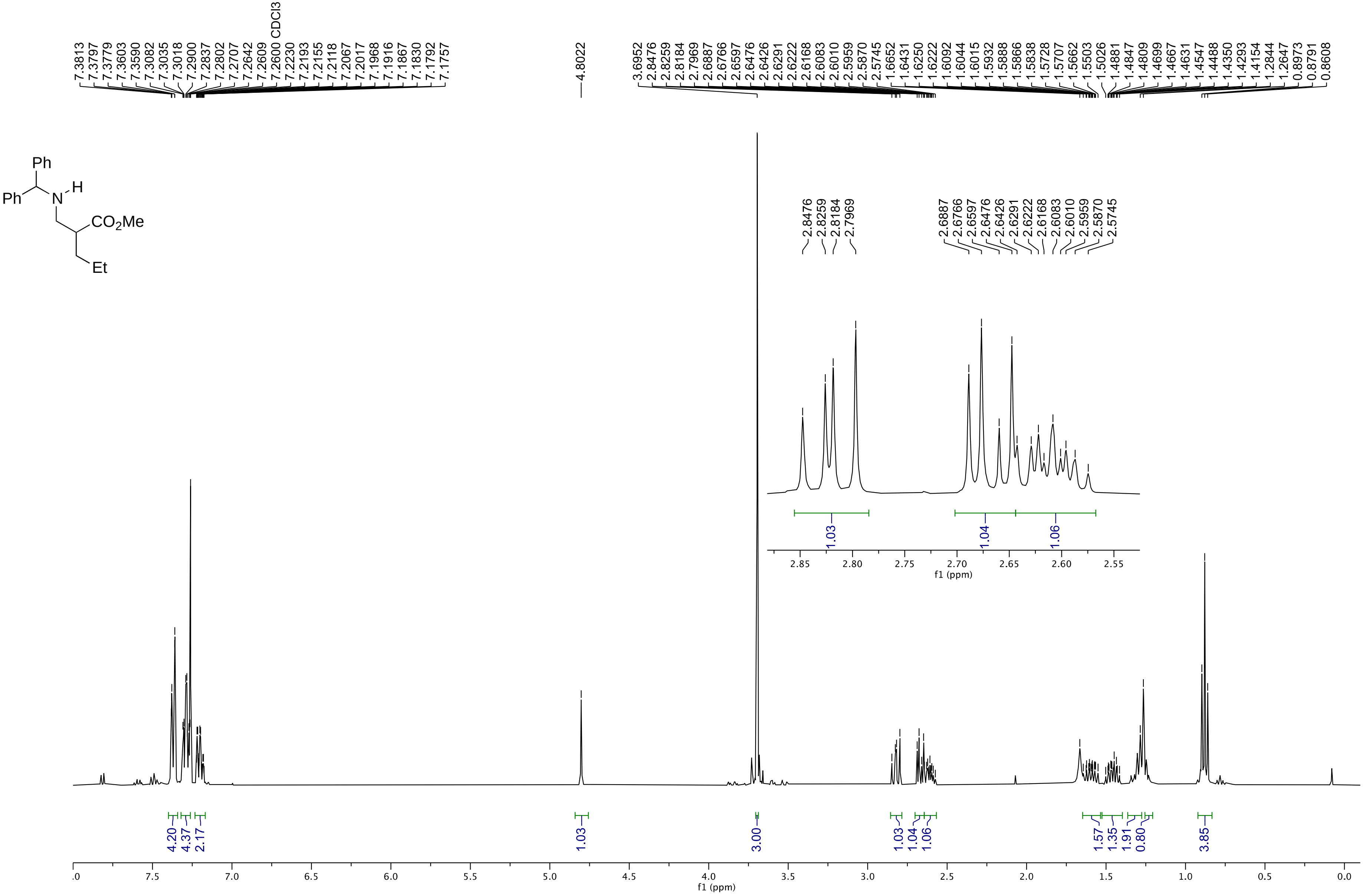

Methyl 2-((diphenylmethyl)aminomethyl)pentanoate (11) – ^{13}C NMR (100 MHz, $CDCl_3$)

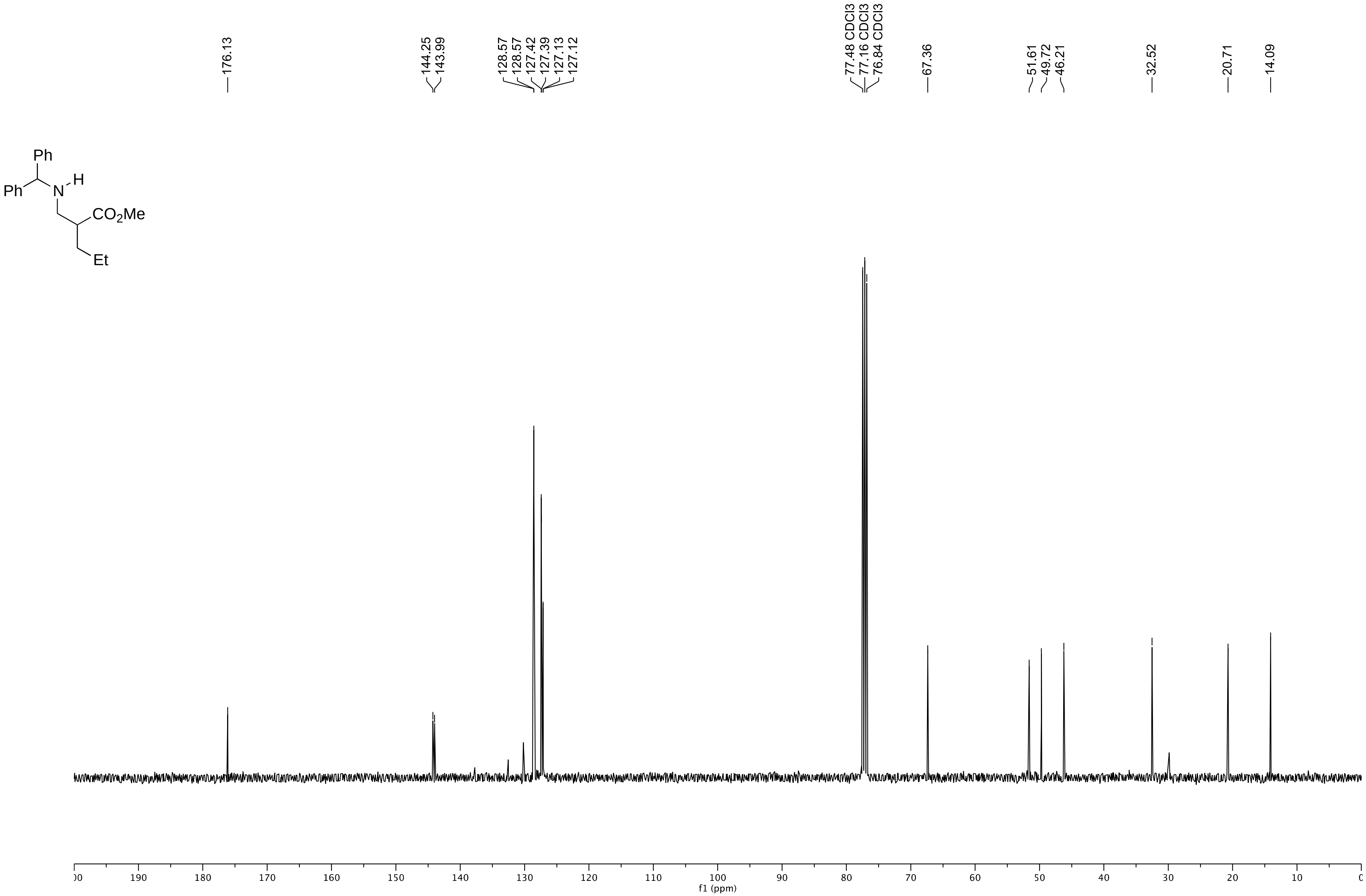

Methyl 2-((phenyl)aminomethyl)pentanoate (12) – ^{1}H NMR (400 MHz, $CDCl_3$)

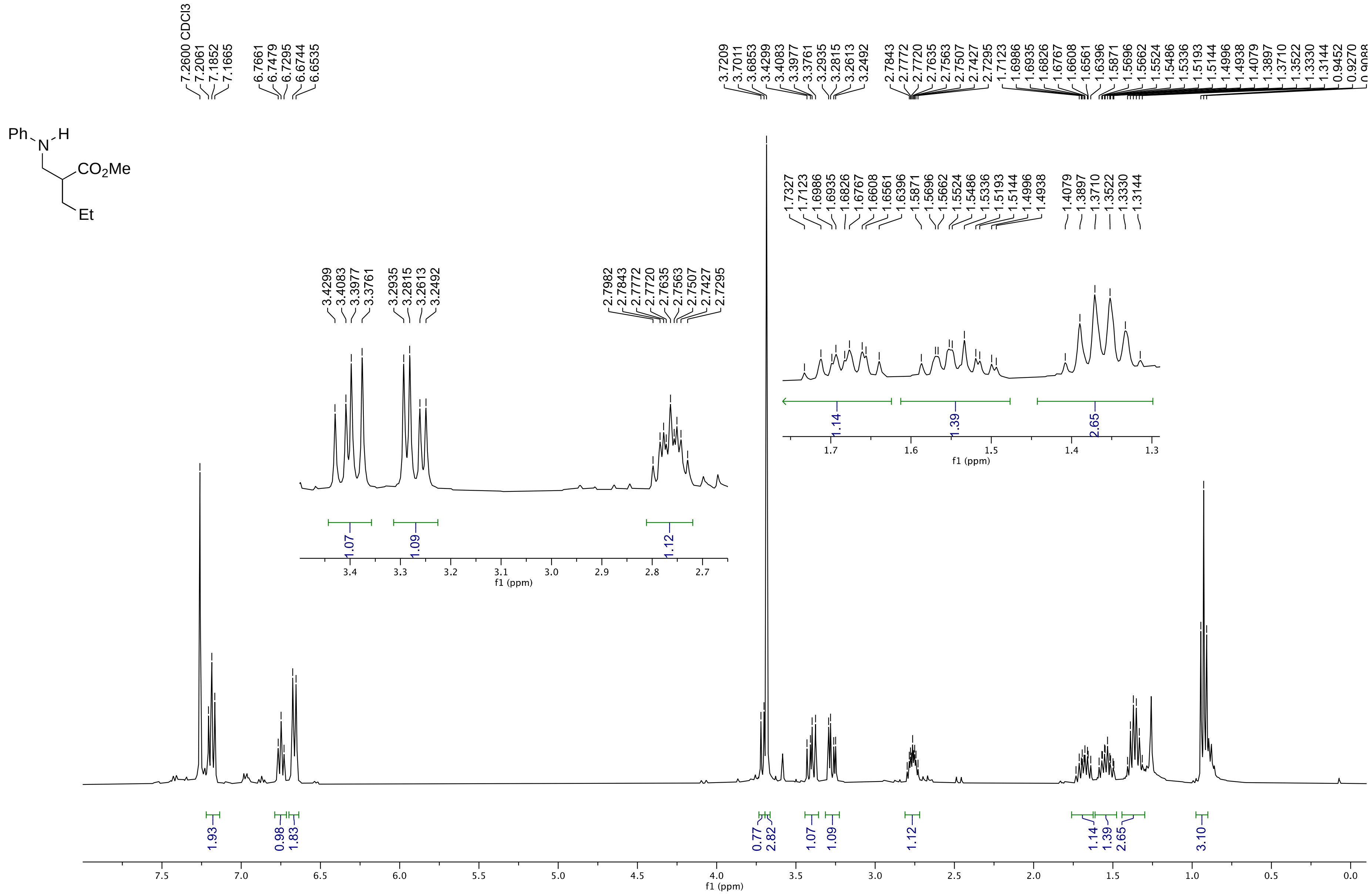

Methyl 2-((phenyl)aminomethyl)pentanoate (12) – ^{13}C NMR (100 MHz, $CDCl_3$)

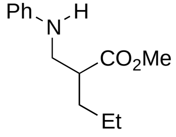

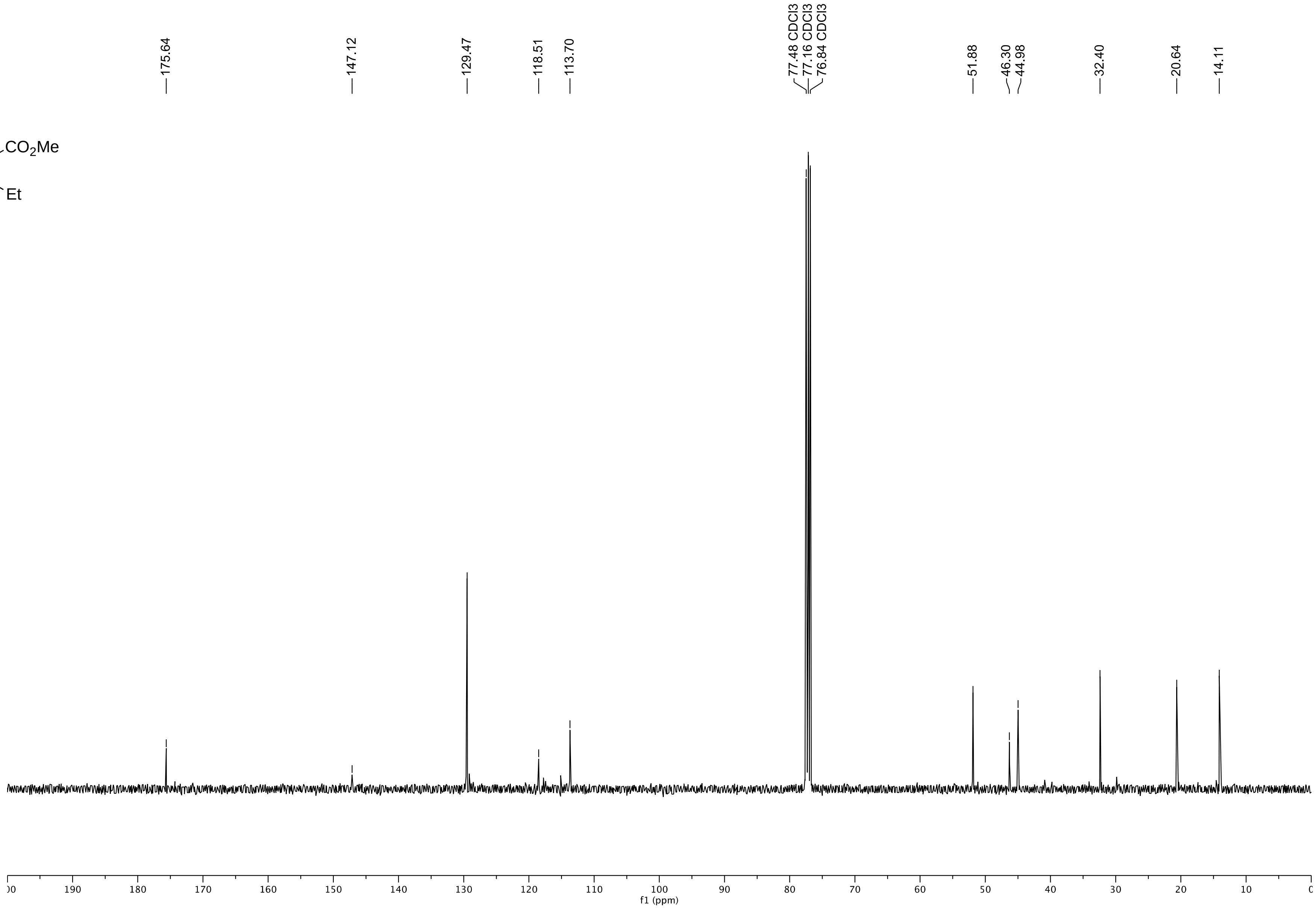

Methyl 2-((tosyl)aminomethyl)pentanoate (13) – ^{1}H NMR (400 MHz, $CDCl_3$)

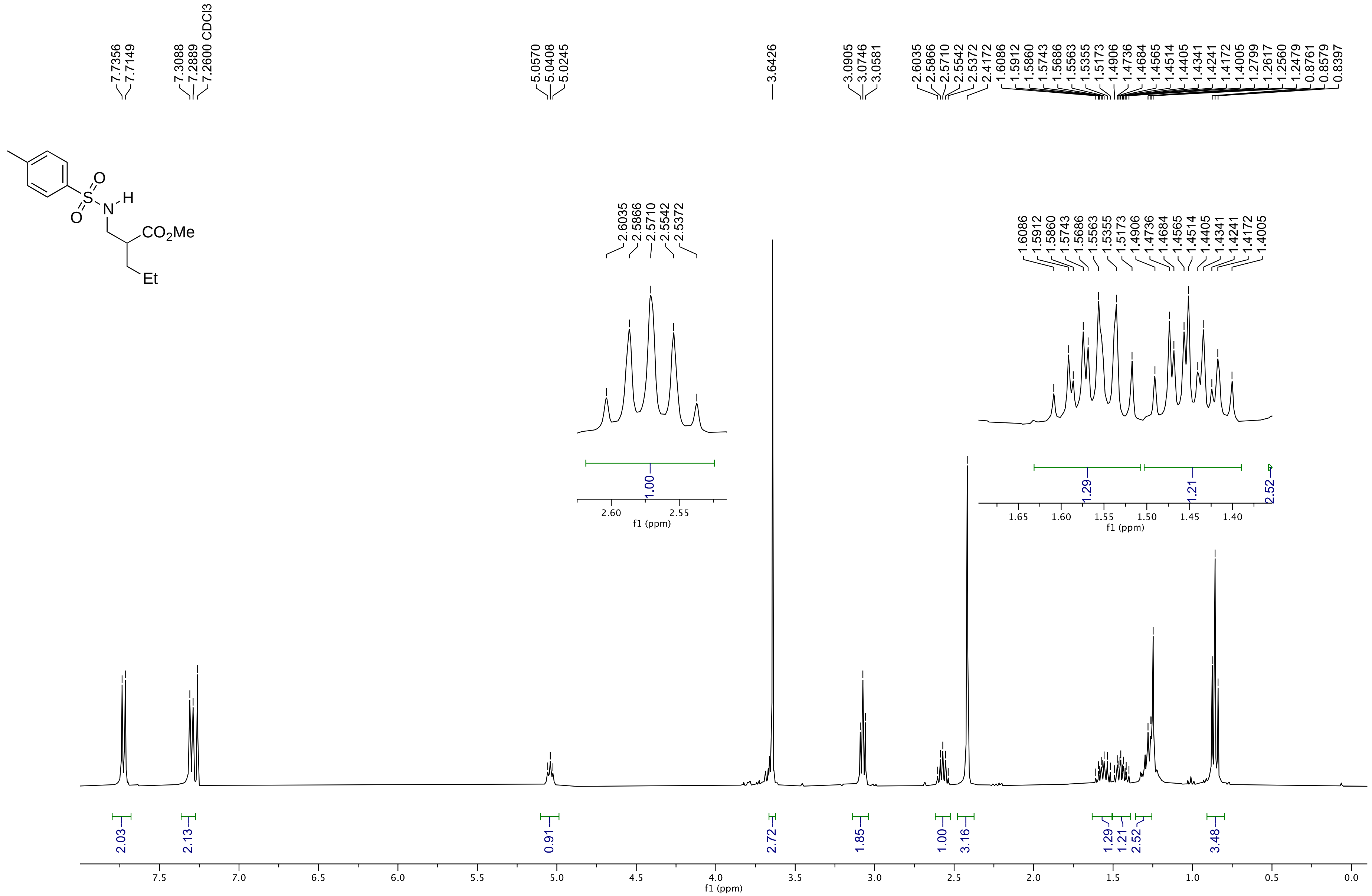

Methyl 2-((tosyl)aminomethyl)pentanoate (13) – ^{13}C NMR (100 MHz, $CDCl_3$)

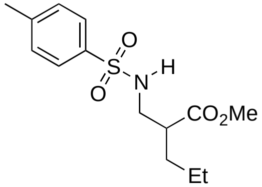

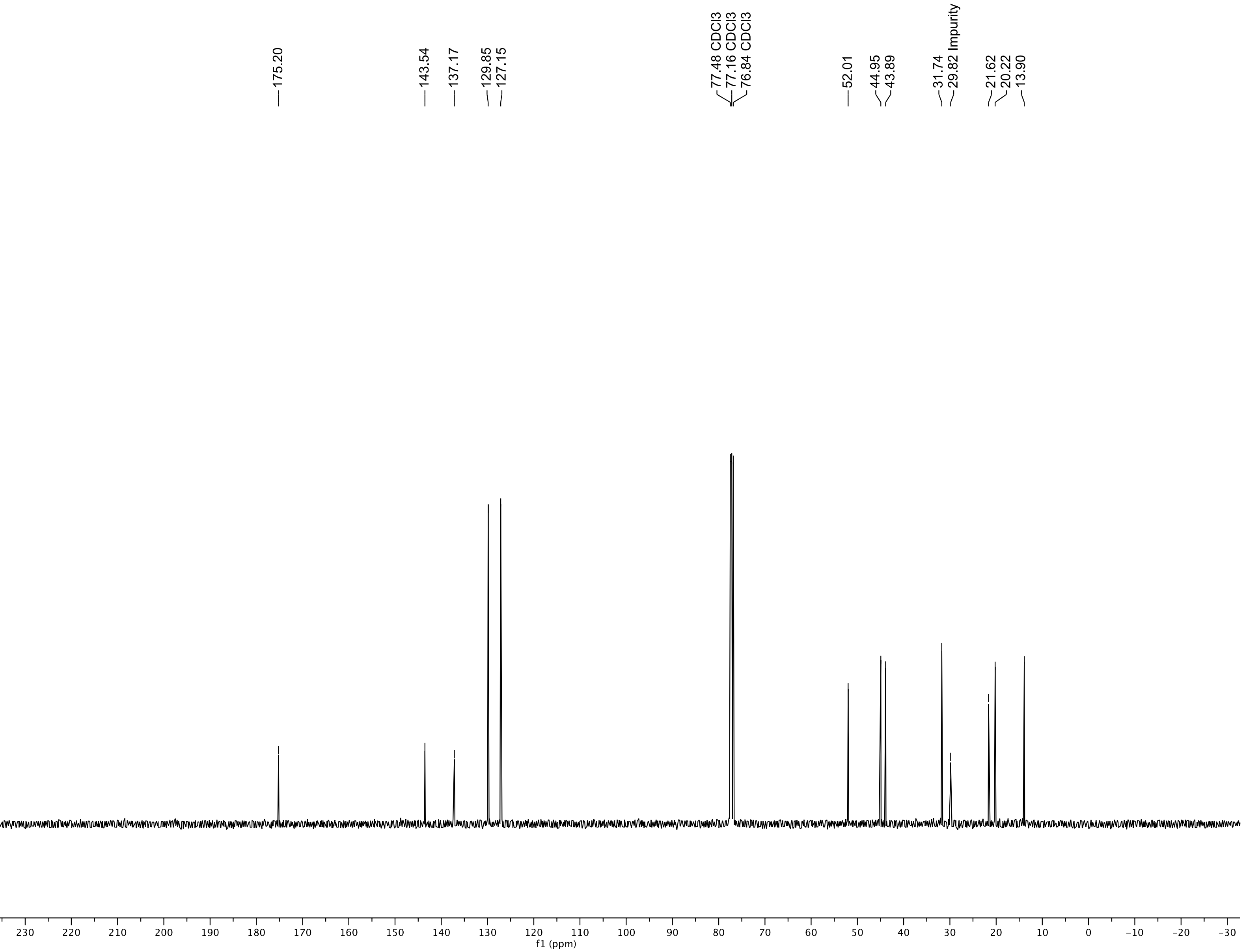

Methyl 2-((*tert*-butanesulfinyl)aminomethyl)pentanoate (14a) – ^{1}H NMR (400 MHz, $CDCl_3$)

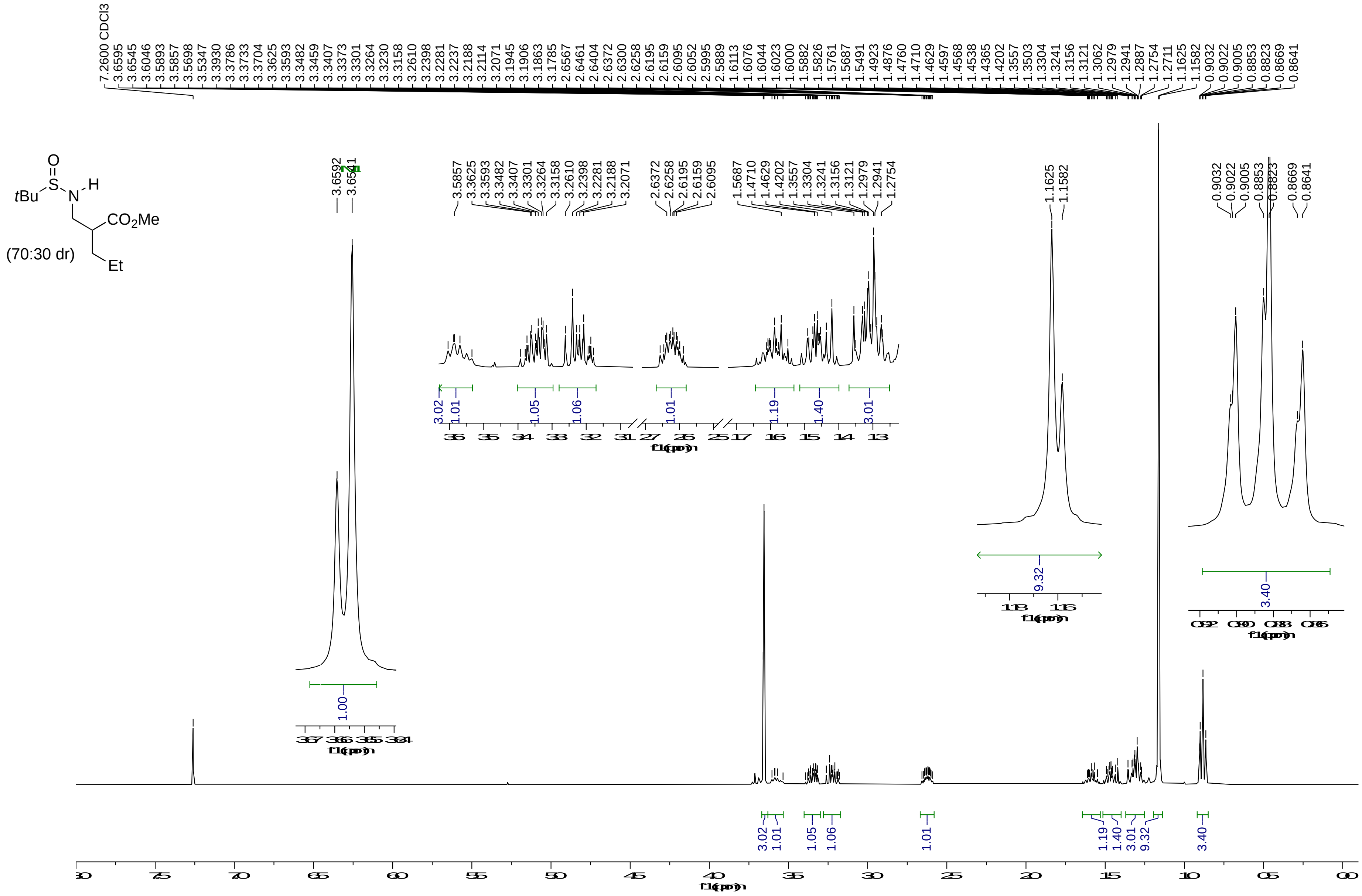

Methyl 2-((*tert*-butanesulfinyl)aminomethyl)pentanoate (14a) – ^{13}C NMR (100 MHz, $CDCl_3$)

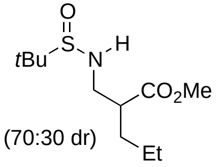

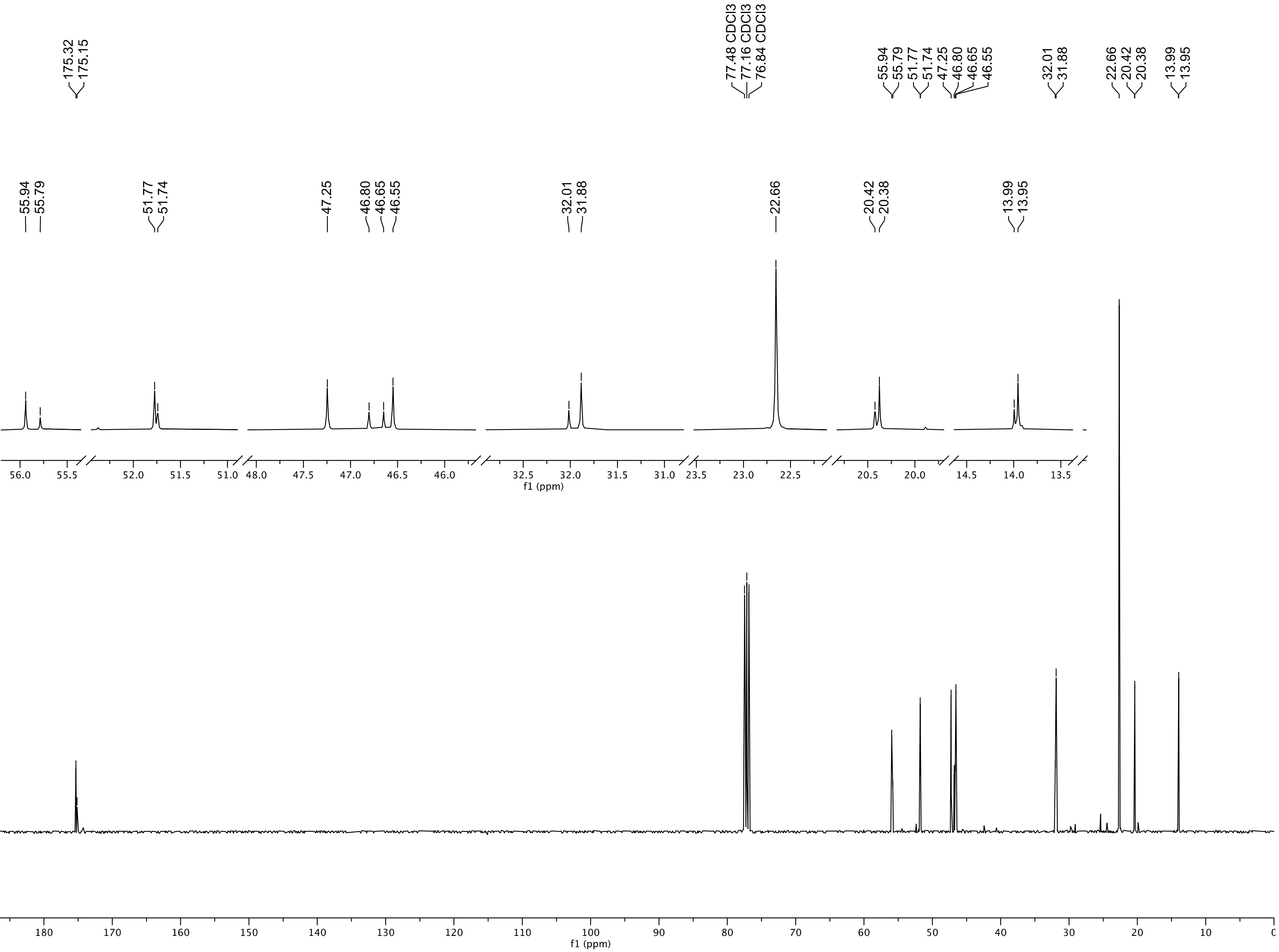

Deuterated methyl 2-((*tert*-butanesulfinyl)aminomethyl)pentanoate (14a-*d*) – ^{1}H NMR (400 MHz, $CDCl_3$)

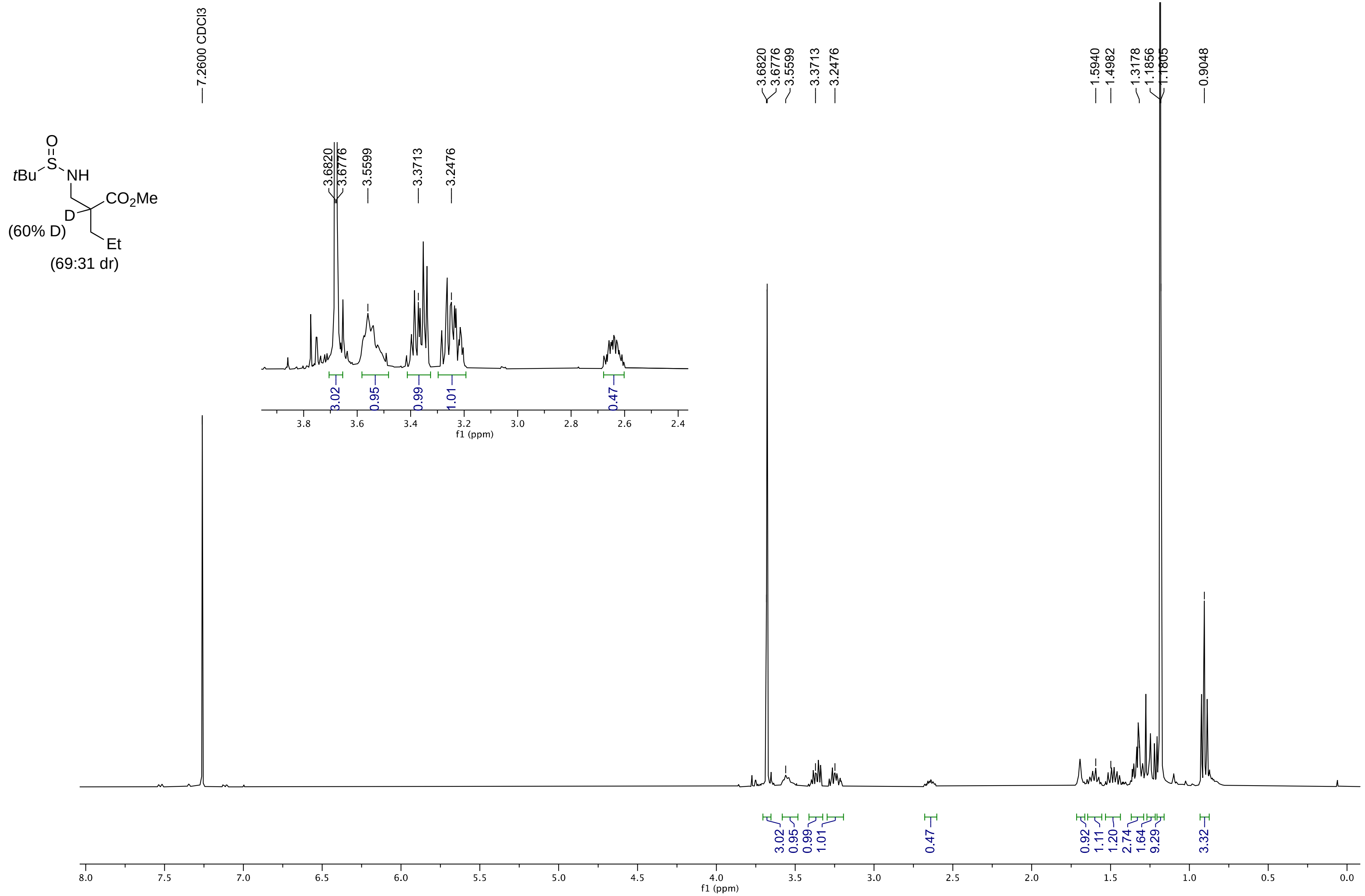

***tert*-Butyl 2-((*tert*-butanesulfinyl)aminomethyl)pentanoate (14b) –** ^{1}H NMR (400 MHz, $CDCl_3$)

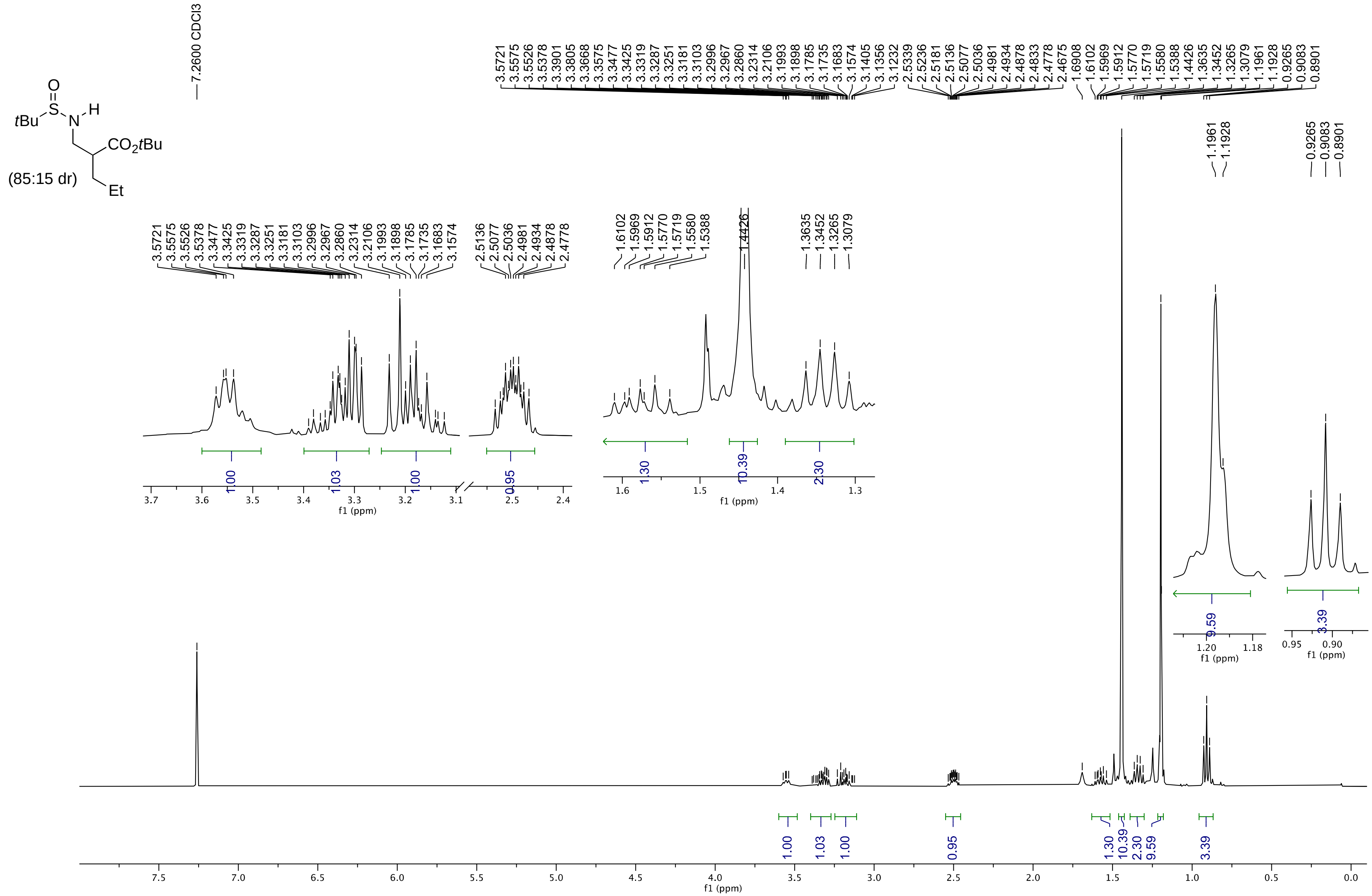

***tert*-Butyl 2-((*tert*-butanesulfinyl)aminomethyl)pentanoate (14b) –** ^{13}C NMR (100 MHz, $CDCl_3$)

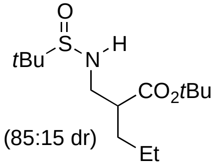

(85:15 dr)

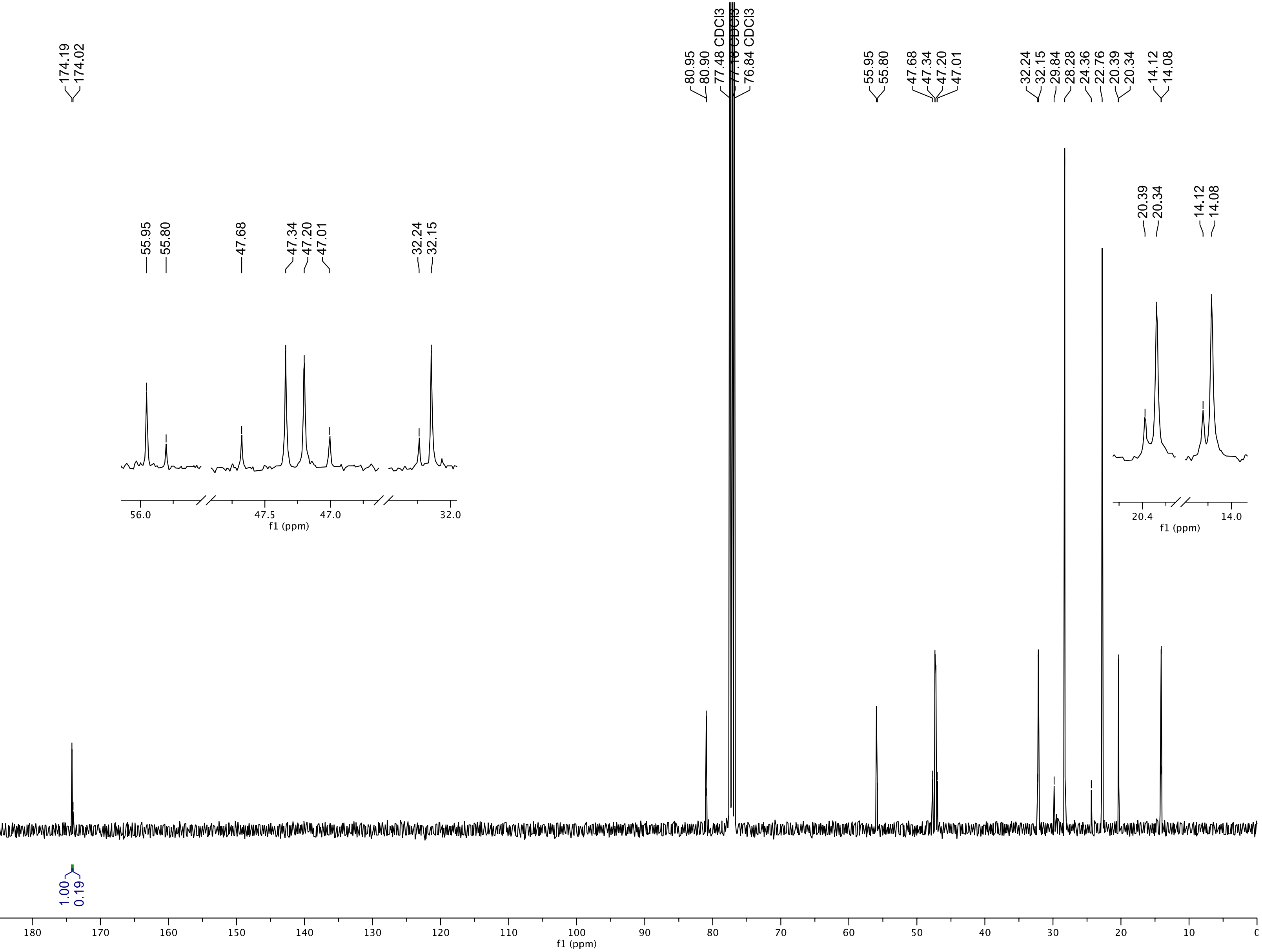

Benzyl 2-((*tert*-butanesulfinyl)aminomethyl)pentanoate (14c) – ^{1}H NMR (400 MHz, $CDCl_3$)

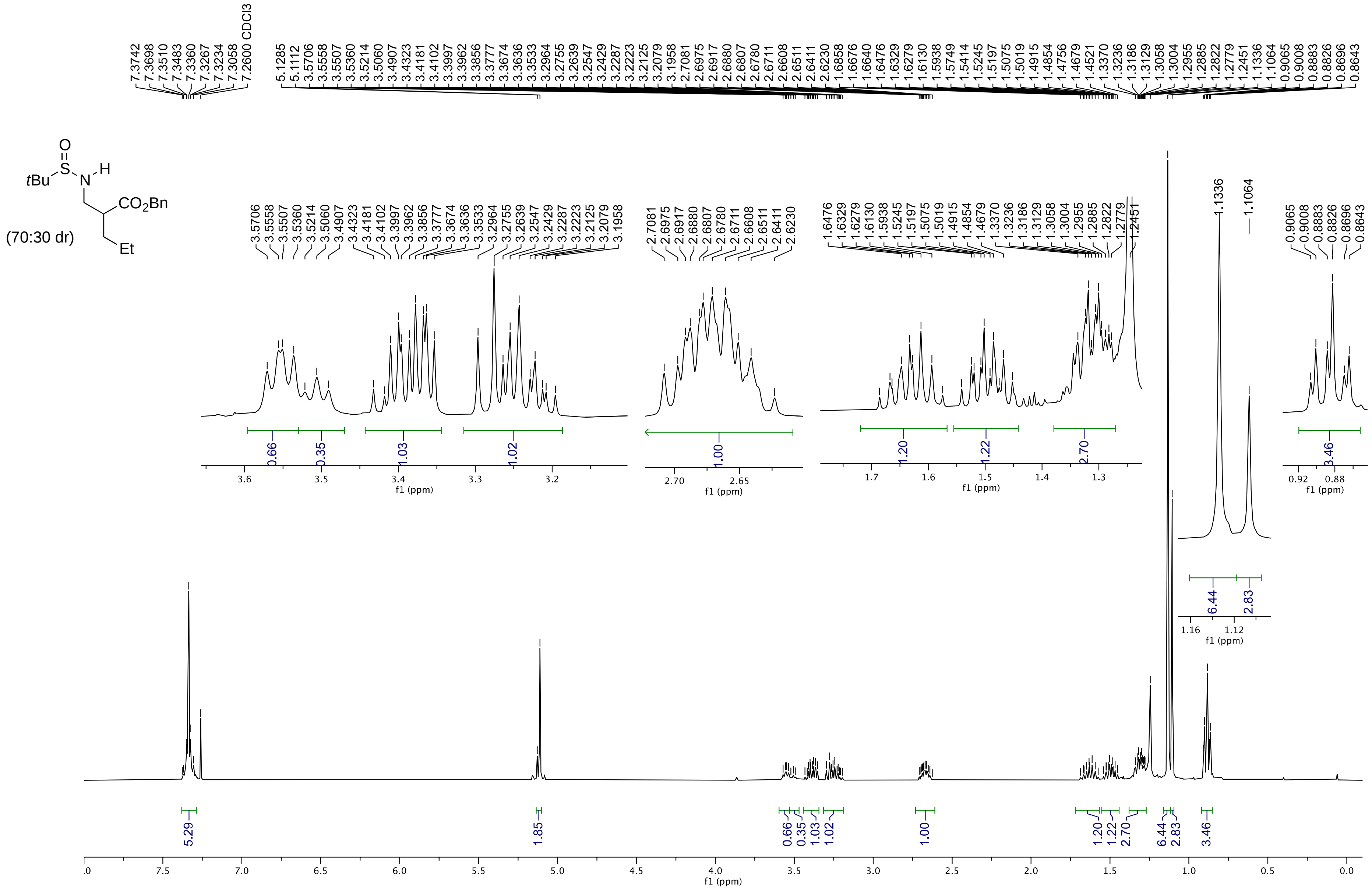

Benzyl 2-((*tert*-butanesulfinyl)aminomethyl)pentanoate (14c) – ^{13}C NMR (100 MHz, $CDCl_3$)

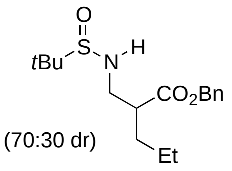

(70:30 dr)

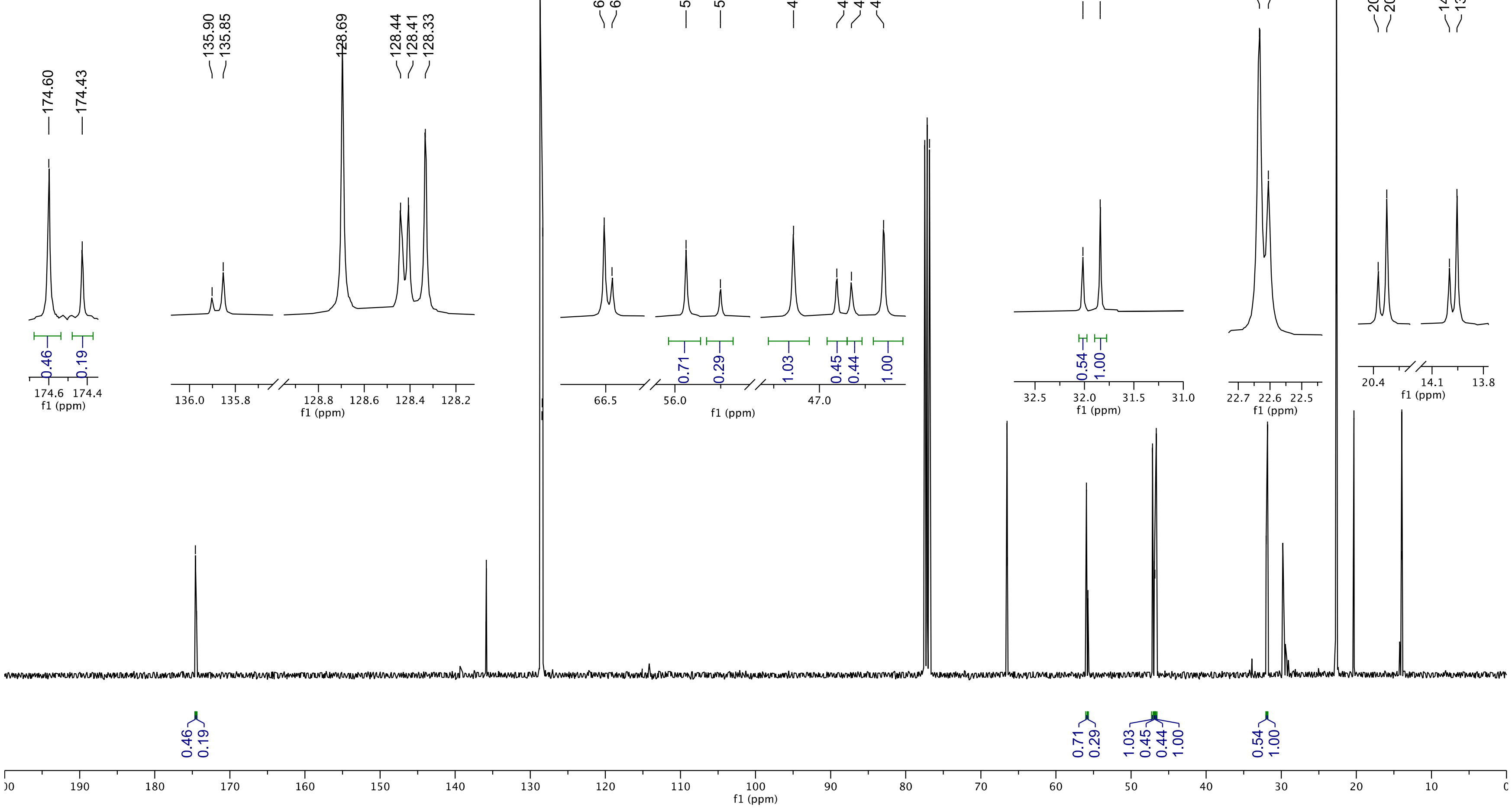

Methyl 2-((*tert*-butanesulfinyl)aminomethyl)heptanoate (15a) – ^{1}H NMR (400 MHz, $CDCl_3$)

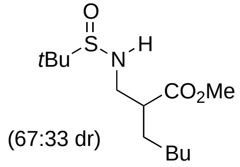

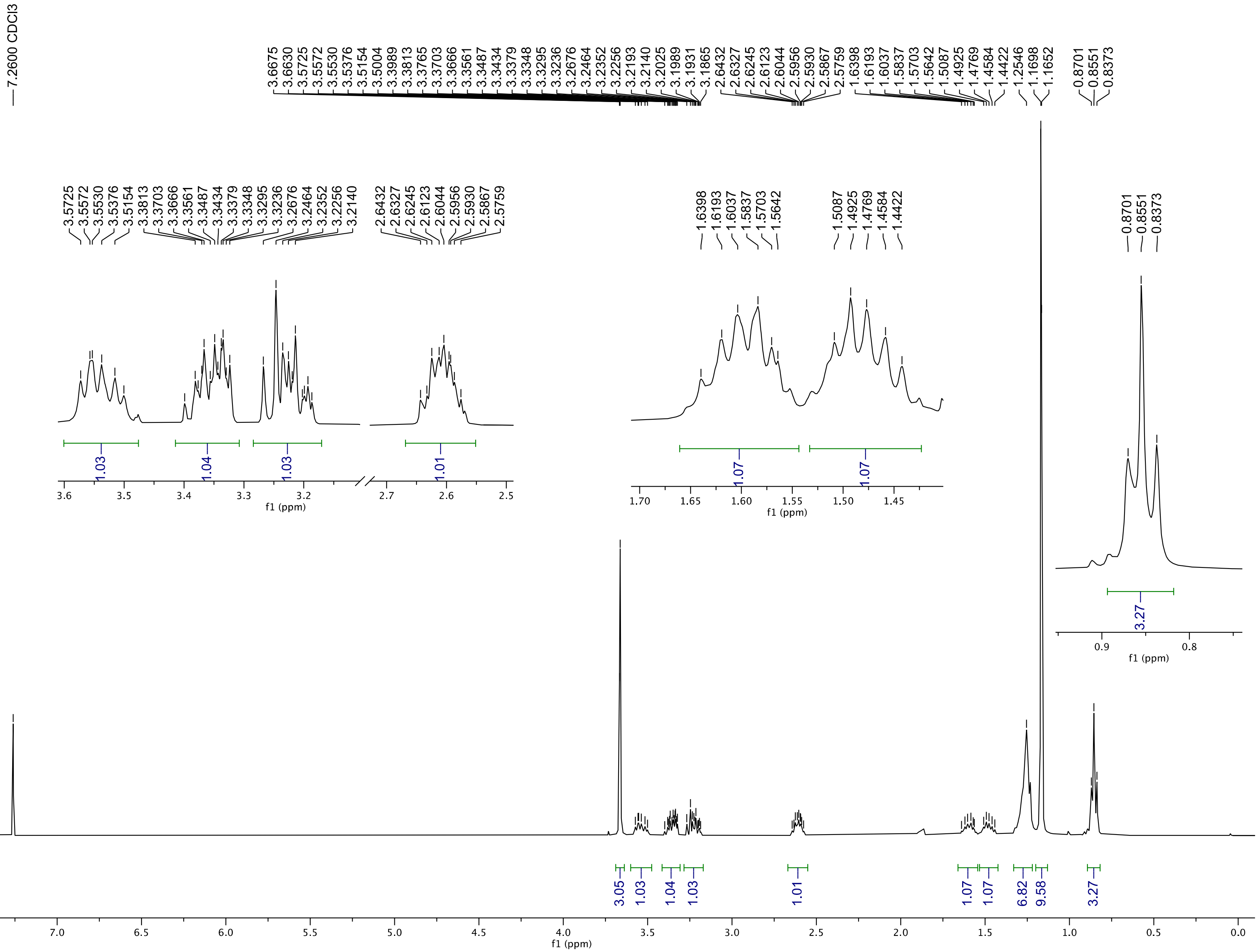

Methyl 2-((*tert*-butanesulfinyl)aminomethyl)heptanoate (15a) – ^{13}C NMR (100 MHz, $CDCl_3$)

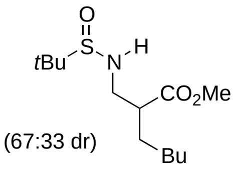

(67:33 dr)

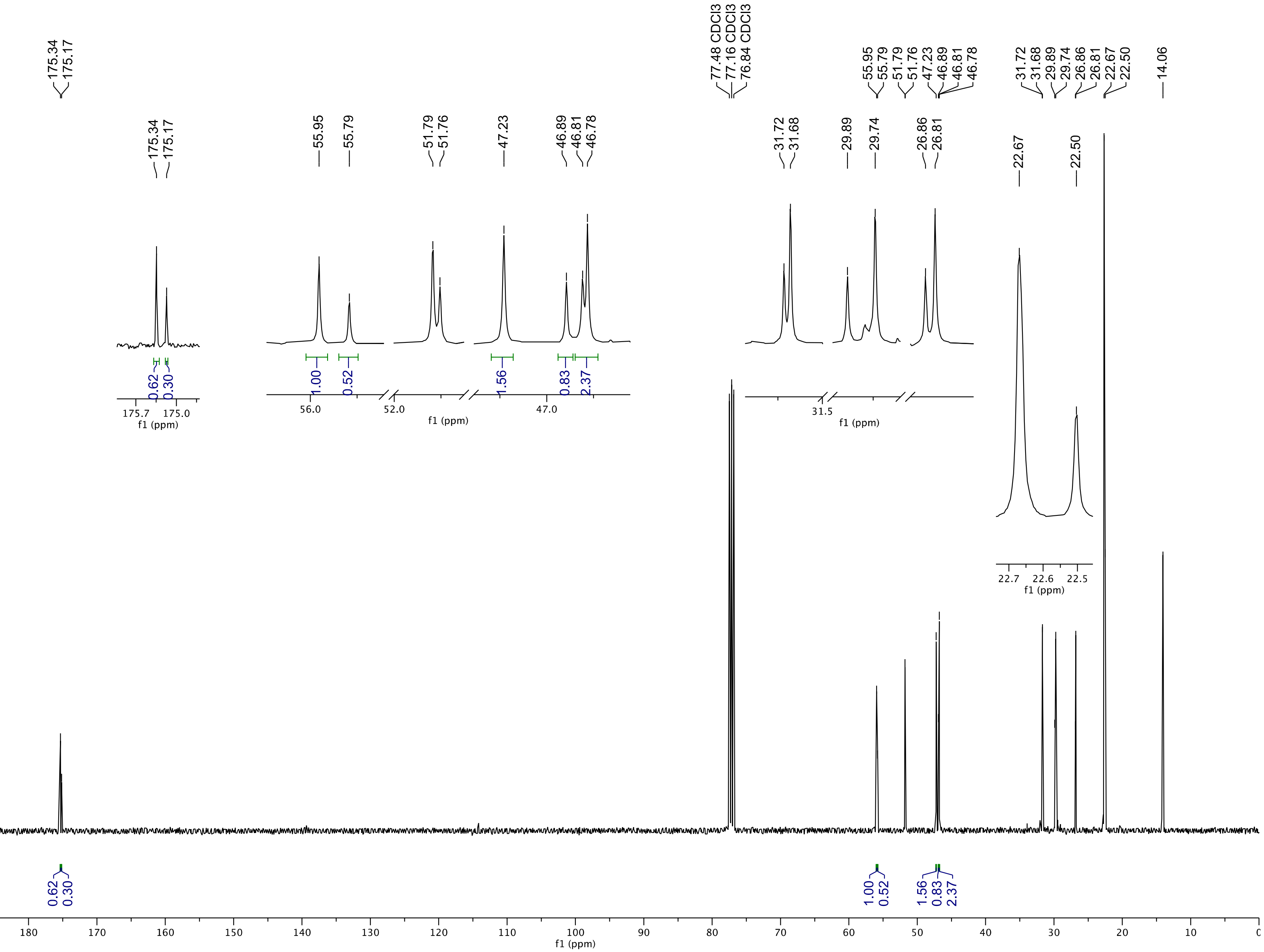

Methyl 2-((diphenylmethyl)aminomethyl)-2-(hydroxycyclohexyl)pentanoate (18) – ^{1}H NMR (400 MHz, $CDCl_3$)

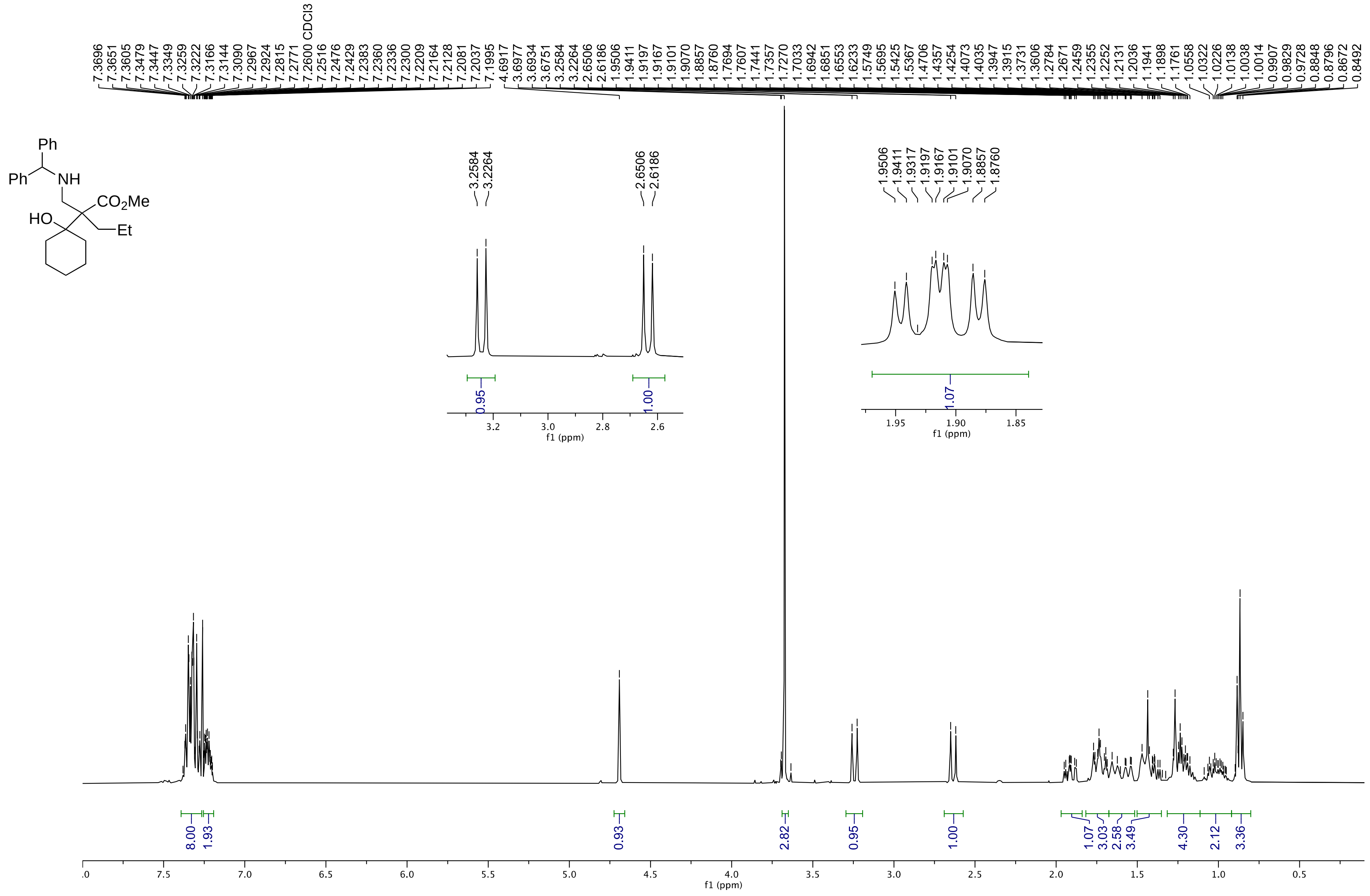

Methyl 2-((diphenylmethyl)aminomethyl)-2-(hydroxycyclohexyl)pentanoate (18) – ^{13}C NMR (100 MHz, $CDCl_3$)

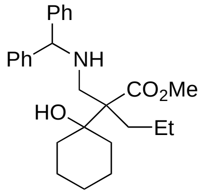

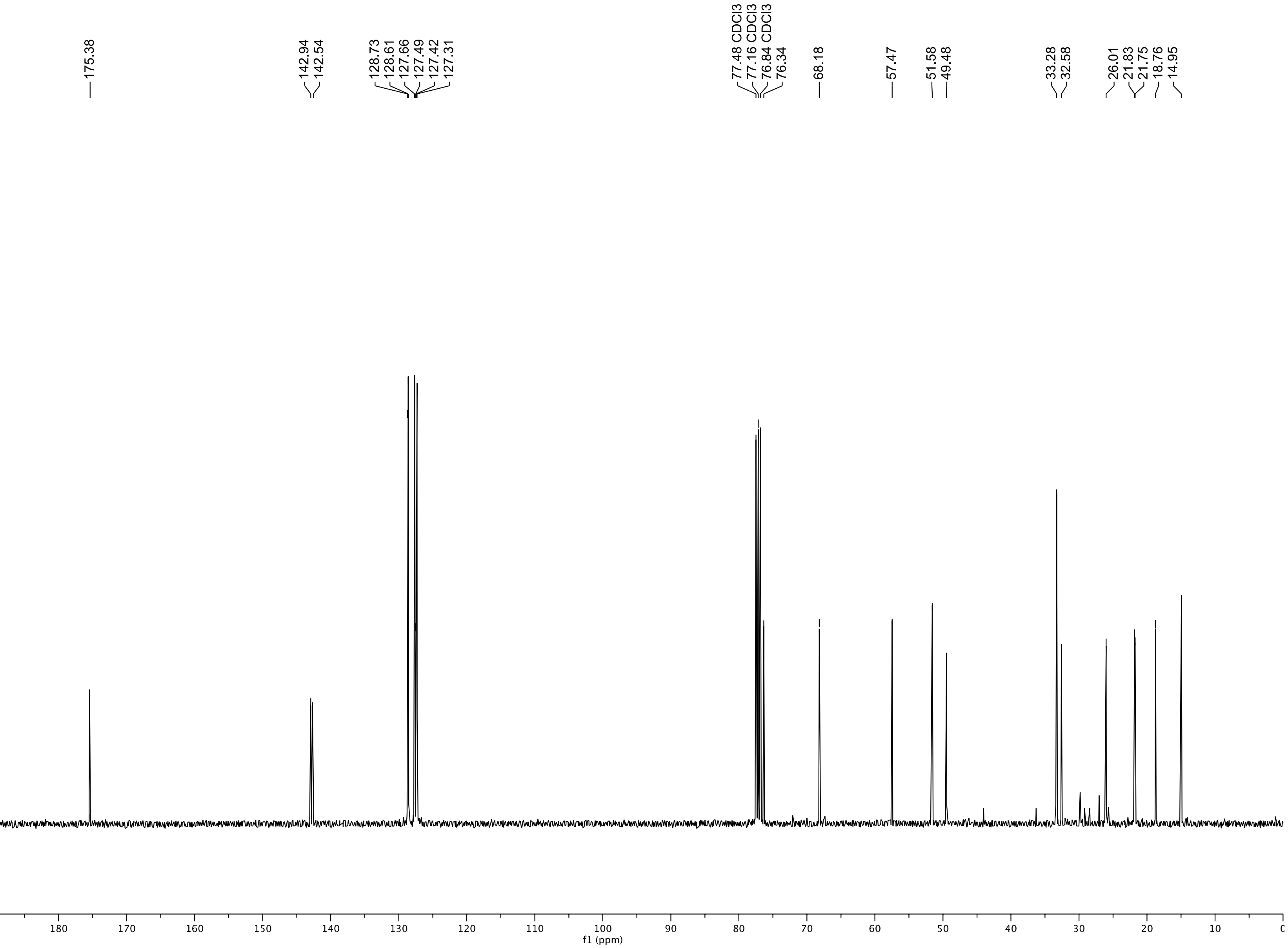

Methyl 2-((phenyl)aminomethyl)-2-(hydroxycyclohexyl)pentanoate (19) – ^{1}H NMR (400 MHz, $CDCl_3$)

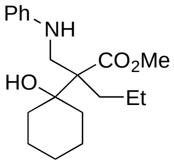

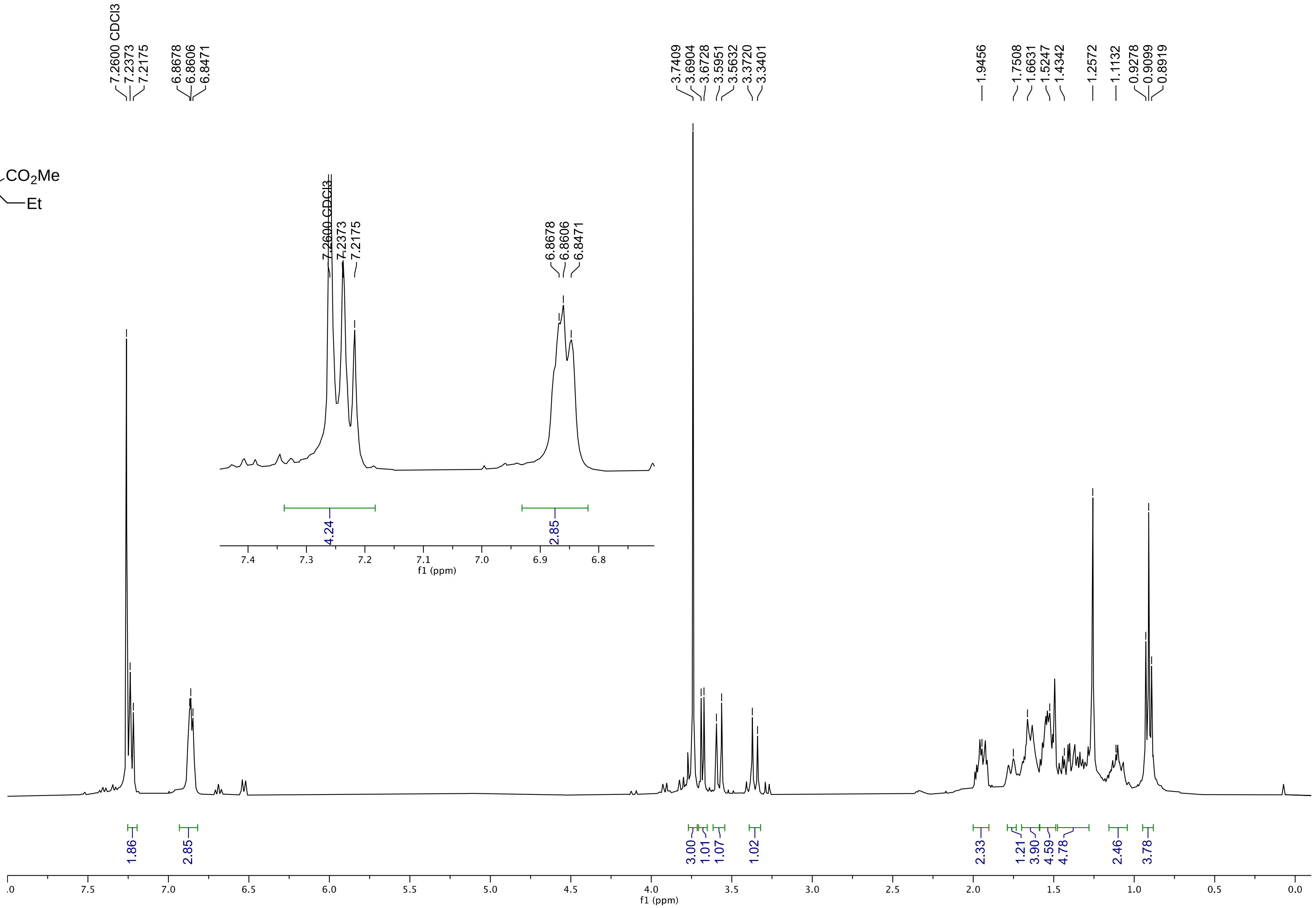

Methyl 2-((phenyl)aminomethyl)-2-(hydroxycyclohexyl)pentanoate (19) – ^{13}C NMR (100 MHz, $CDCl_3$)

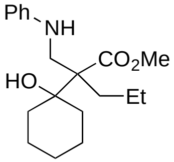

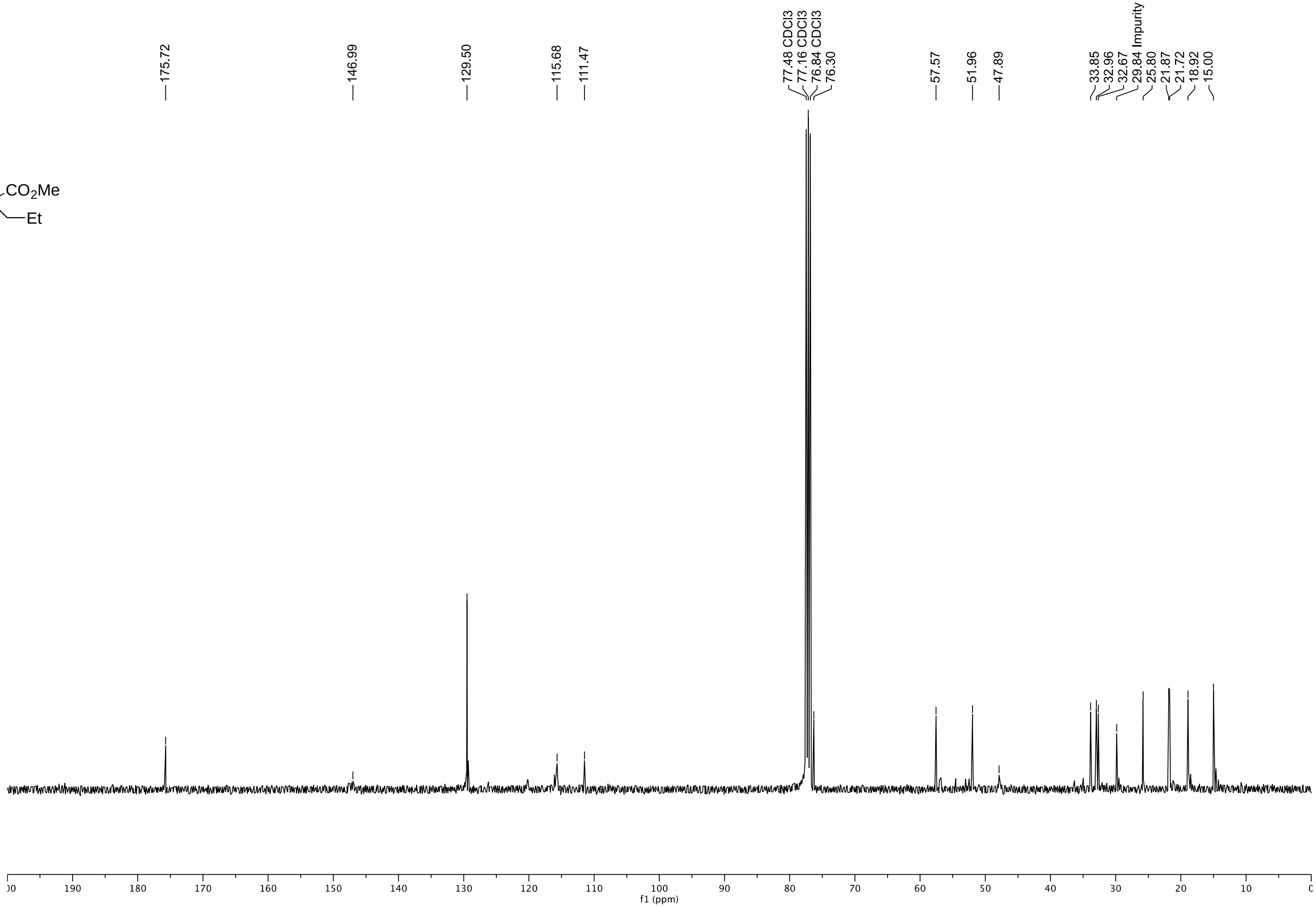

Methyl 2-((tosyl)aminomethyl)-2-(hydroxycyclohexyl)pentanoate (20) – ^{1}H NMR (400 MHz, $CDCl_3$)

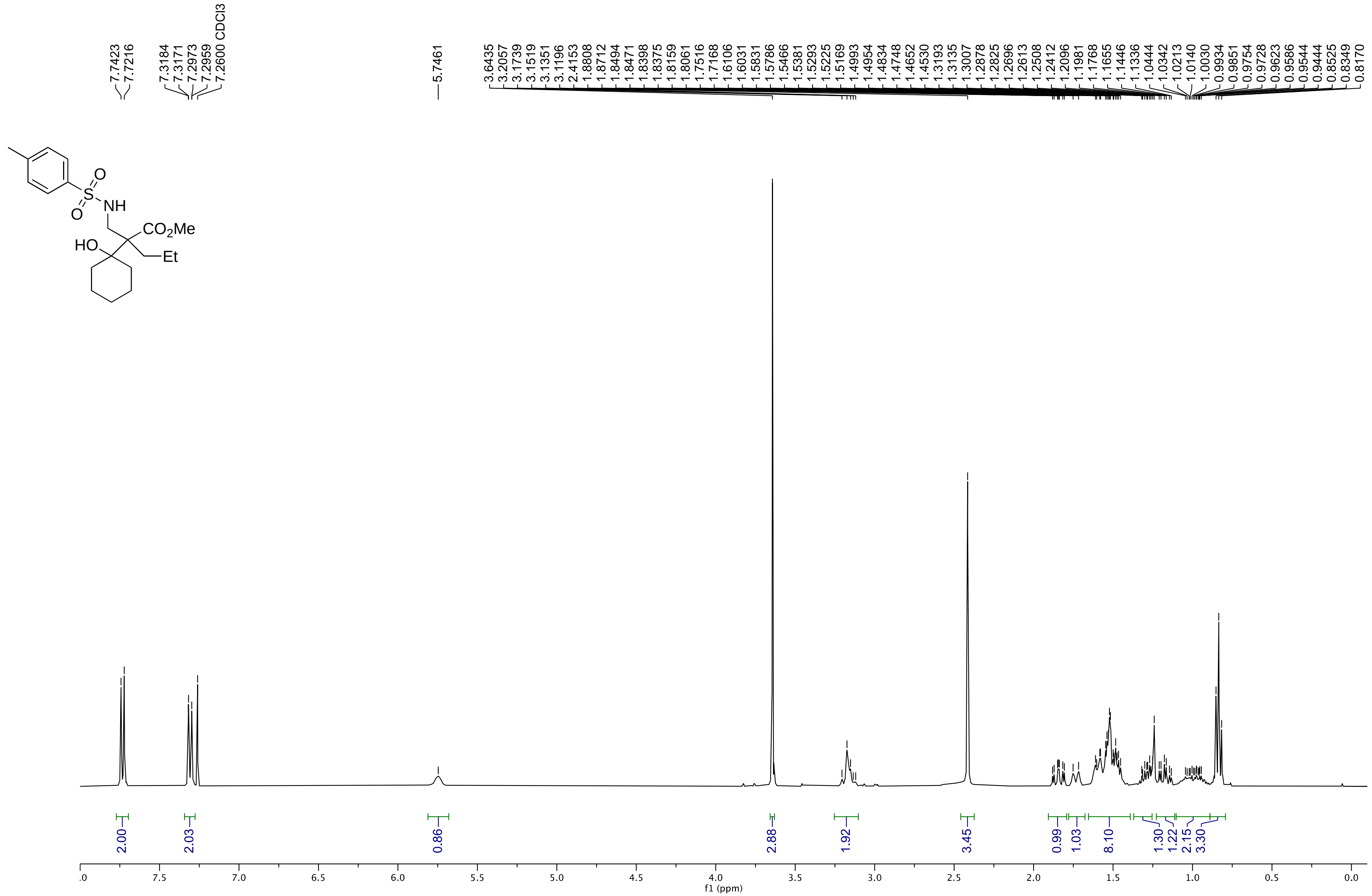

Methyl 2-((tosyl)aminomethyl)-2-(hydroxycyclohexyl)pentanoate (20) – ^{13}C NMR (100 MHz, $CDCl_3$)

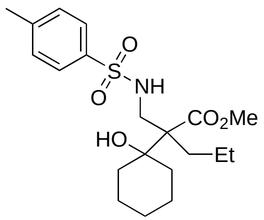

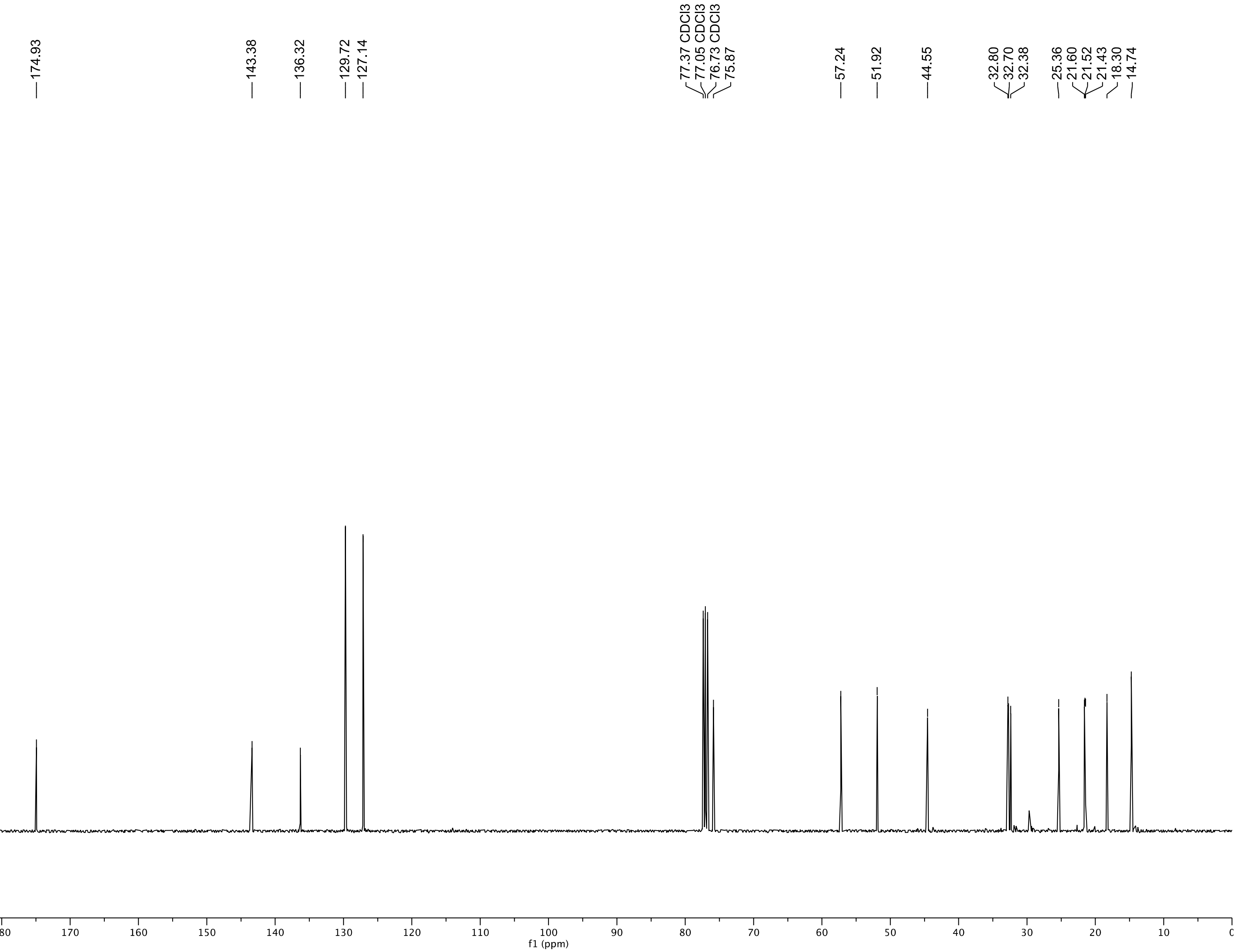

Methyl 2-((*tert*-butanesulfinyl)aminomethyl)-2-(hydroxycyclohexyl)pentanoate (21a) – ^{1}H NMR (400 MHz, $CDCl_3$)

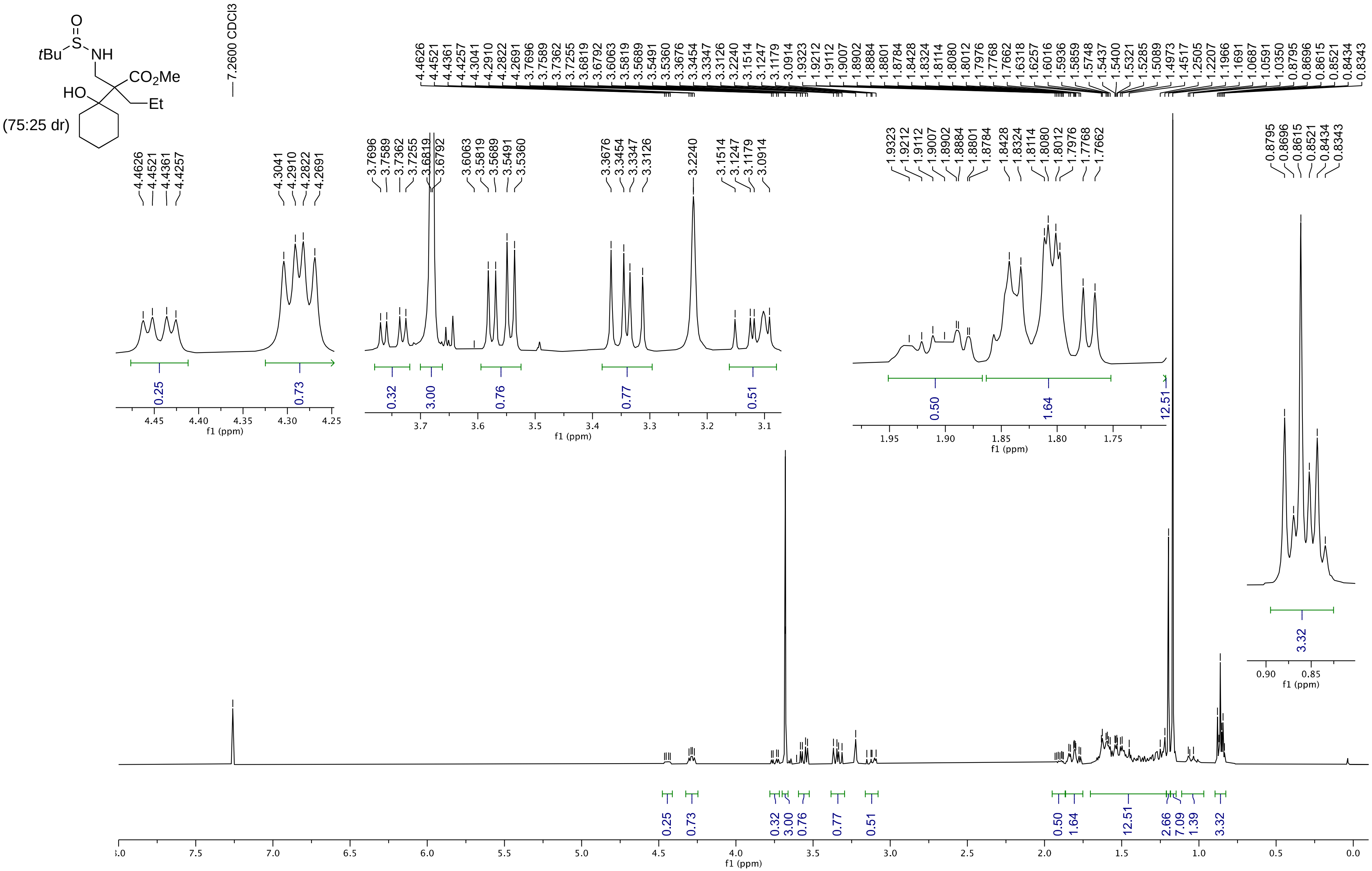

Methyl 2-((*tert*-butanesulfinyl)aminomethyl)-2-(hydroxycyclohexyl)pentanoate (21a) – ^{13}C NMR (100 MHz, $CDCl_3$)

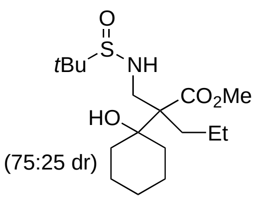

(75:25 dr)

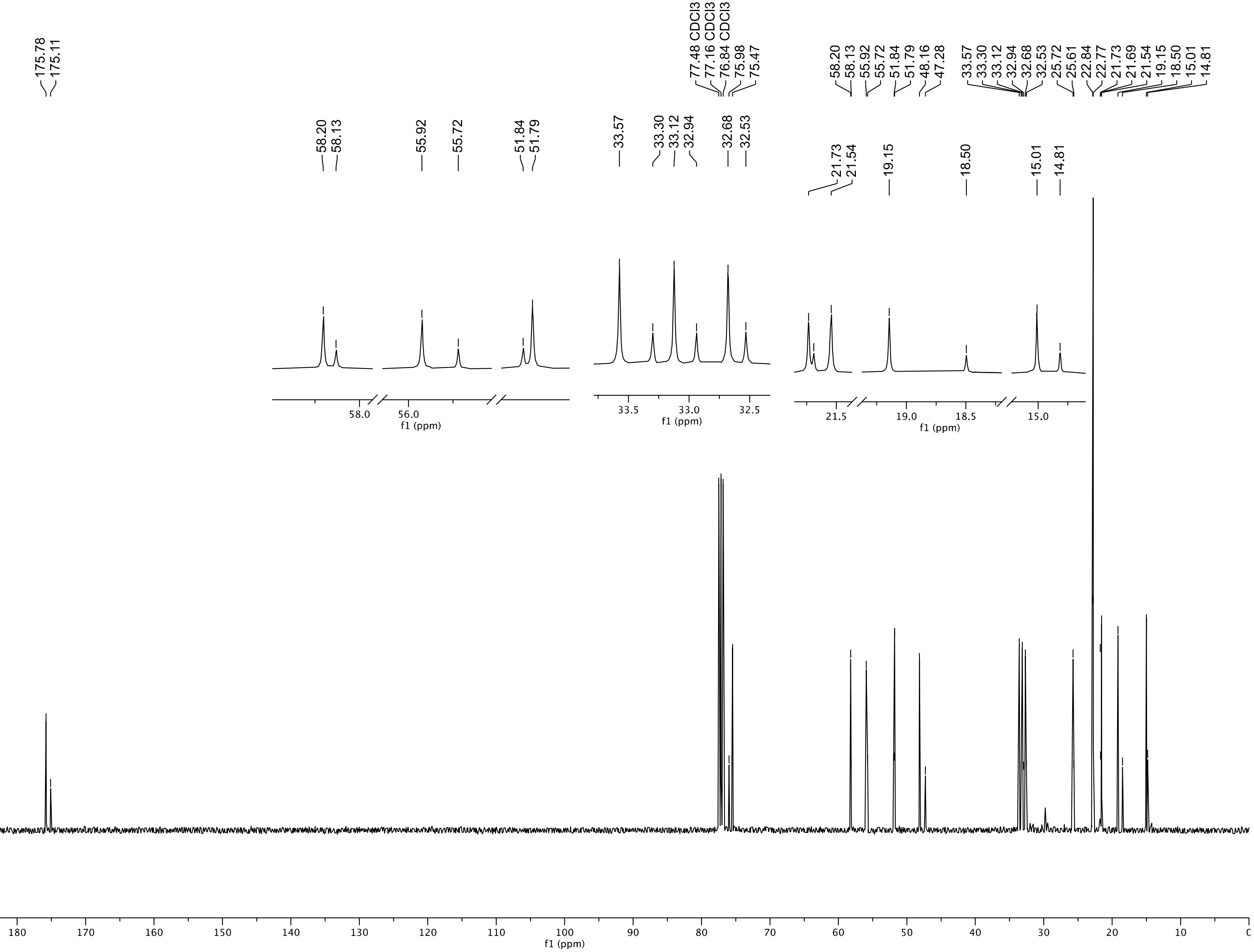

***tert*-Butyl 2-((*tert*-butanesulfinyl)aminomethyl)-2-(hydroxycyclohexyl)pentanoate (21b) –** ^{1}H NMR (400 MHz, $CDCl_3$)

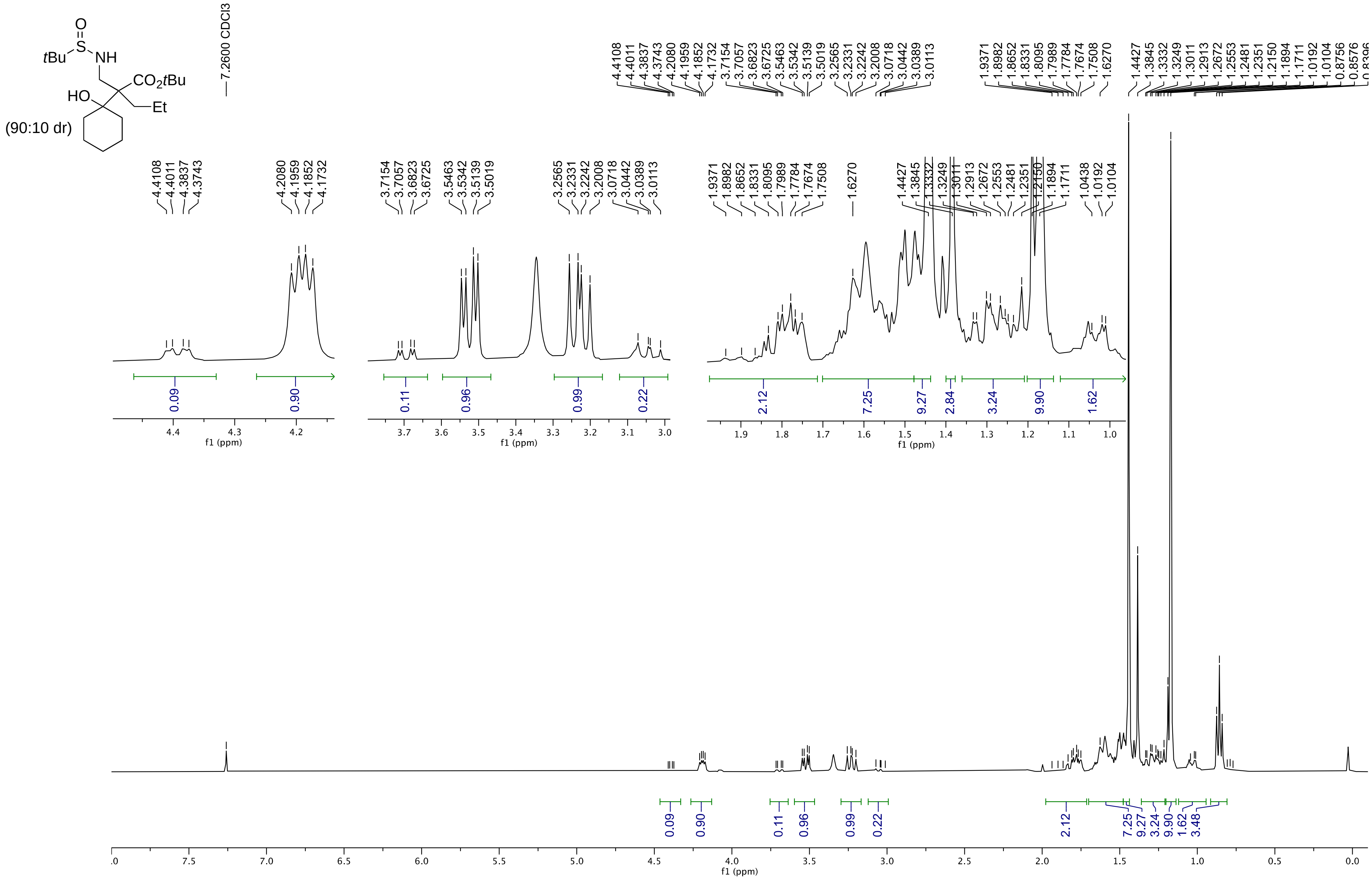

***tert*-Butyl 2-((*tert*-butanesulfinyl)aminomethyl)-2-(hydroxycyclohexyl)pentanoate (21b) –** ^{13}C NMR (100 MHz, $CDCl_3$)

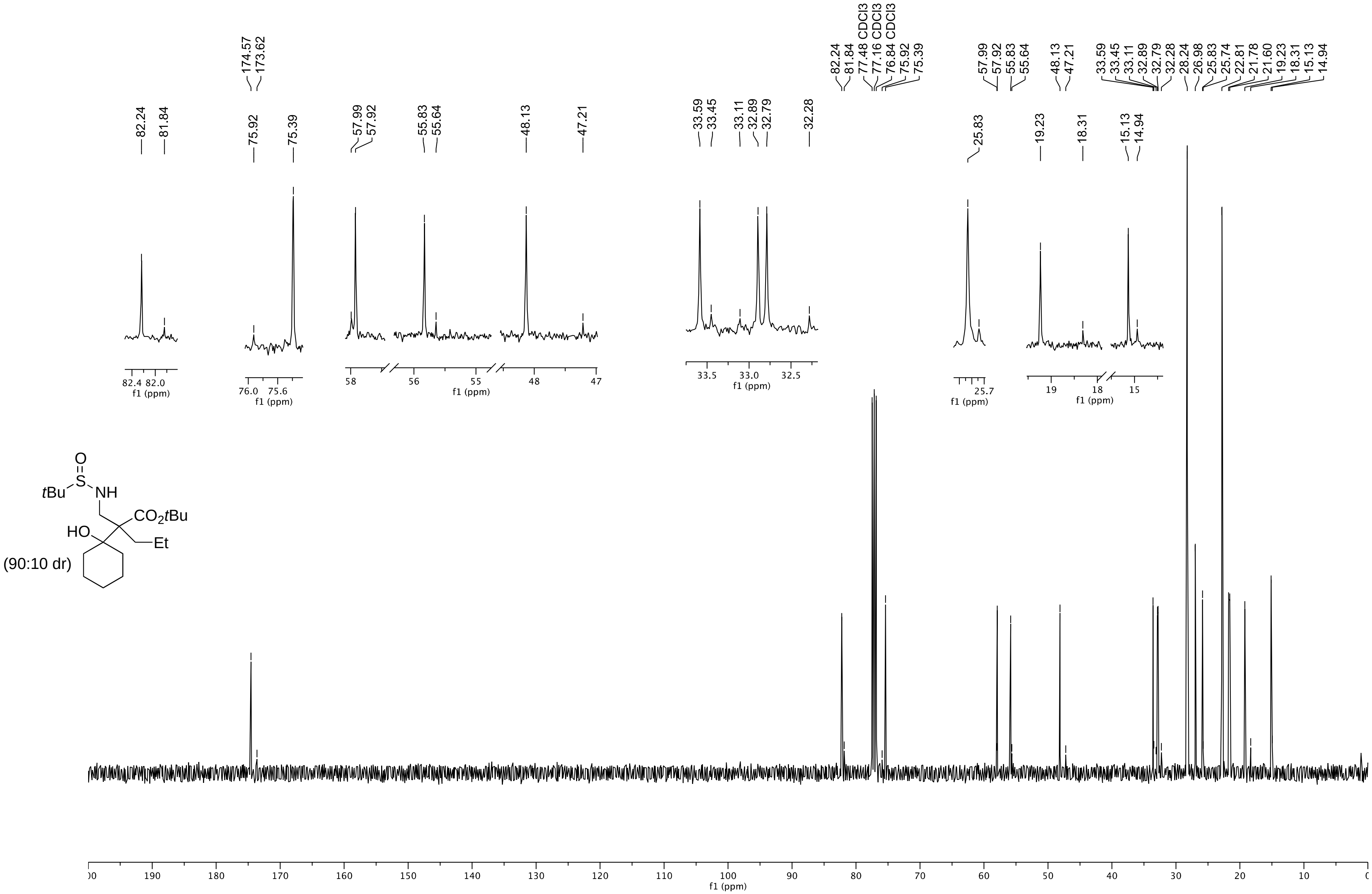

***tert*-Butyl 2-((*tert*-butanesulfinyl)aminomethyl)-2-(1-hydroxy-1-methylethyl)pentanoate (22) –** ^{1}H NMR (400 MHz, $CDCl_3$)

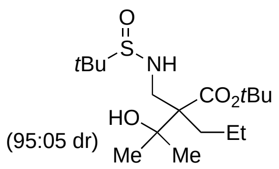

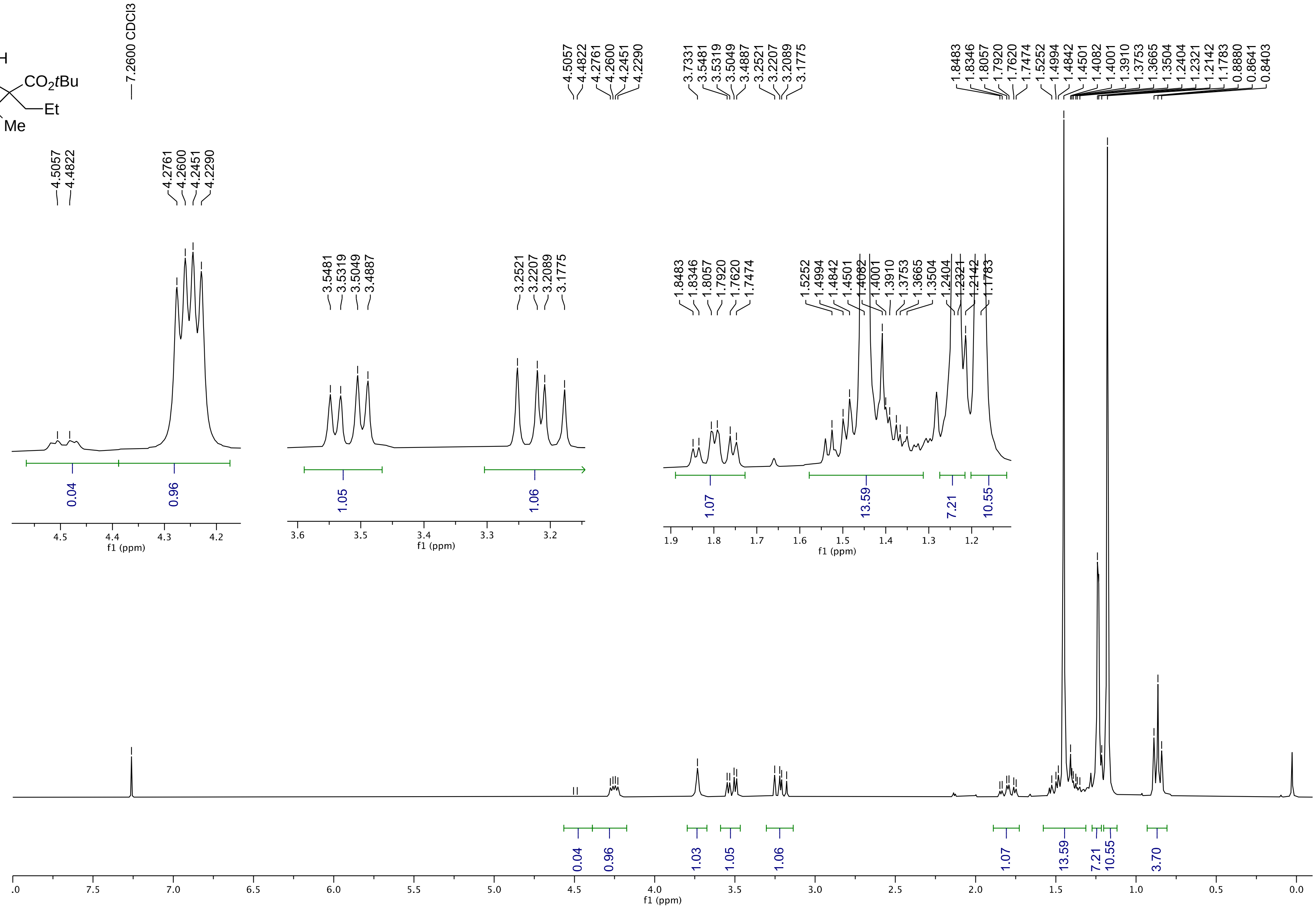

***tert*-Butyl 2-((*tert*-butanesulfinyl)aminomethyl)-2-(1-hydroxy-1-methylethyl)pentanoate (22) –** ^{13}C NMR (100 MHz, $CDCl_3$)

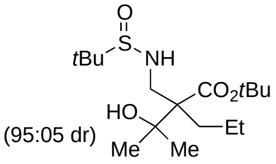

(95:05 dr)

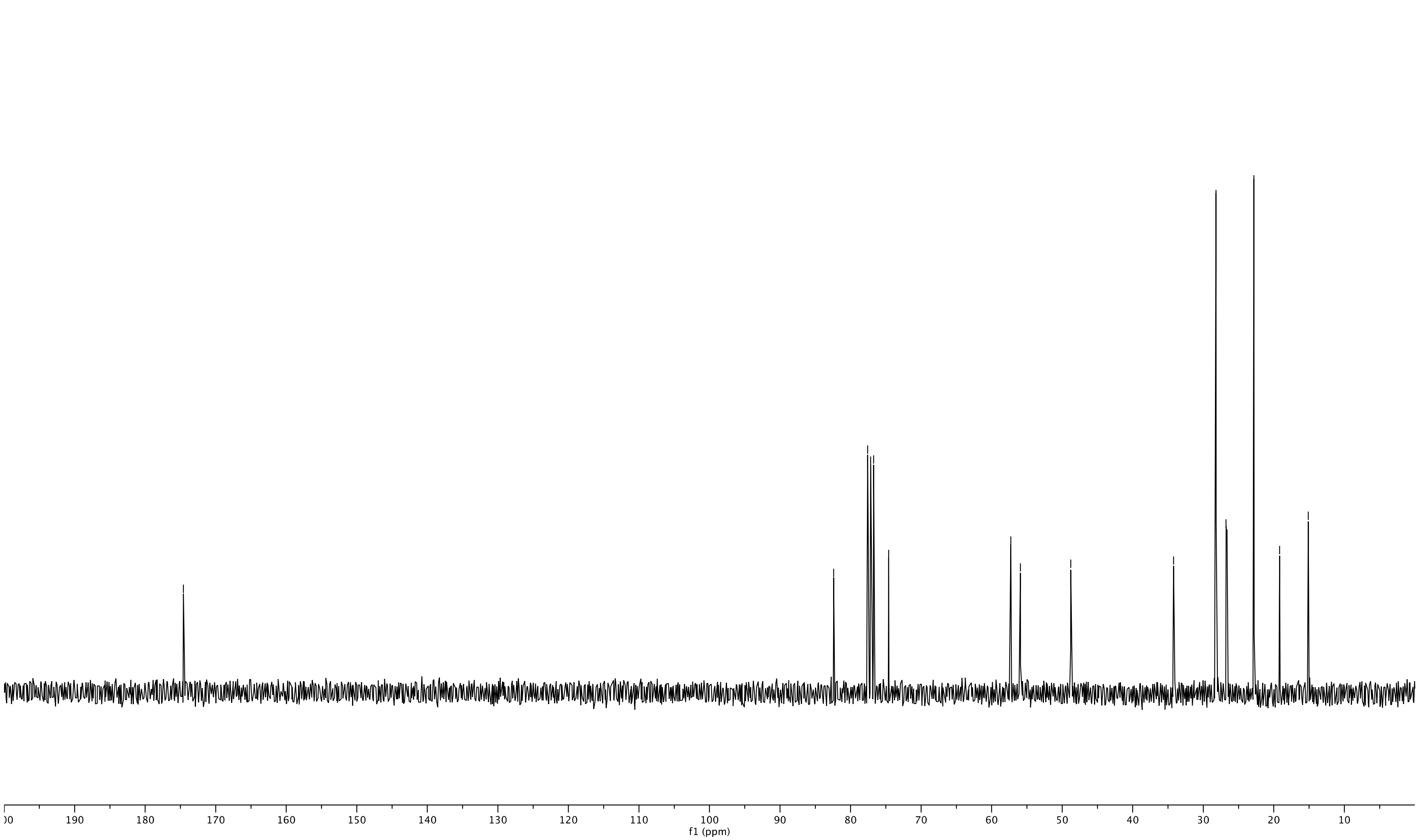

Methyl 2-((diphenylmethyl)aminomethyl)-2-(hydroxy(phenyl)methyl)pentanoate (23) – ^{1}H NMR (400 MHz, $CDCl_3$)

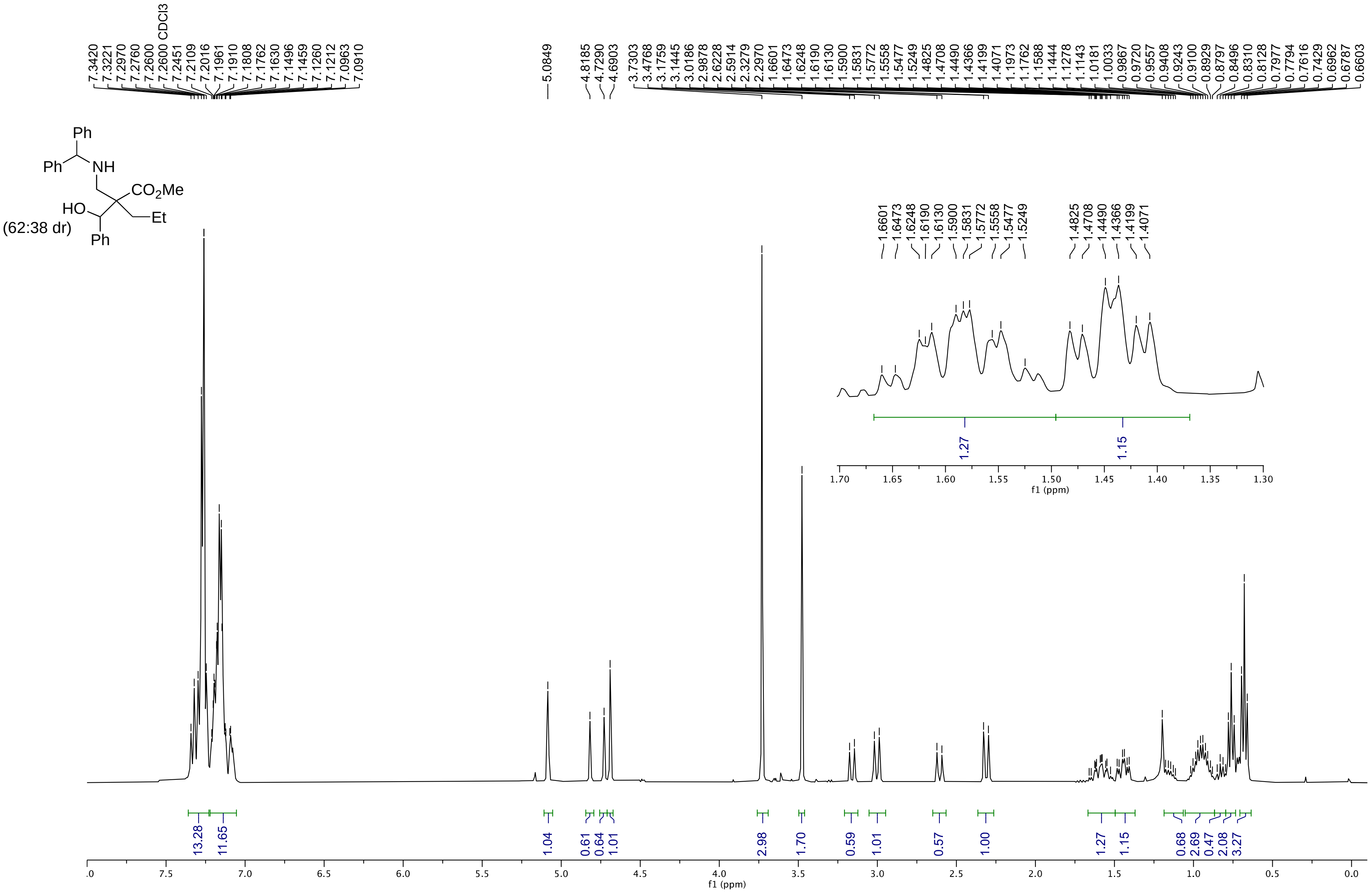

Methyl 2-((diphenylmethyl)aminomethyl)-2-(hydroxy(phenyl)methyl)pentanoate (23) – ^{13}C NMR (100 MHz, $CDCl_3$)

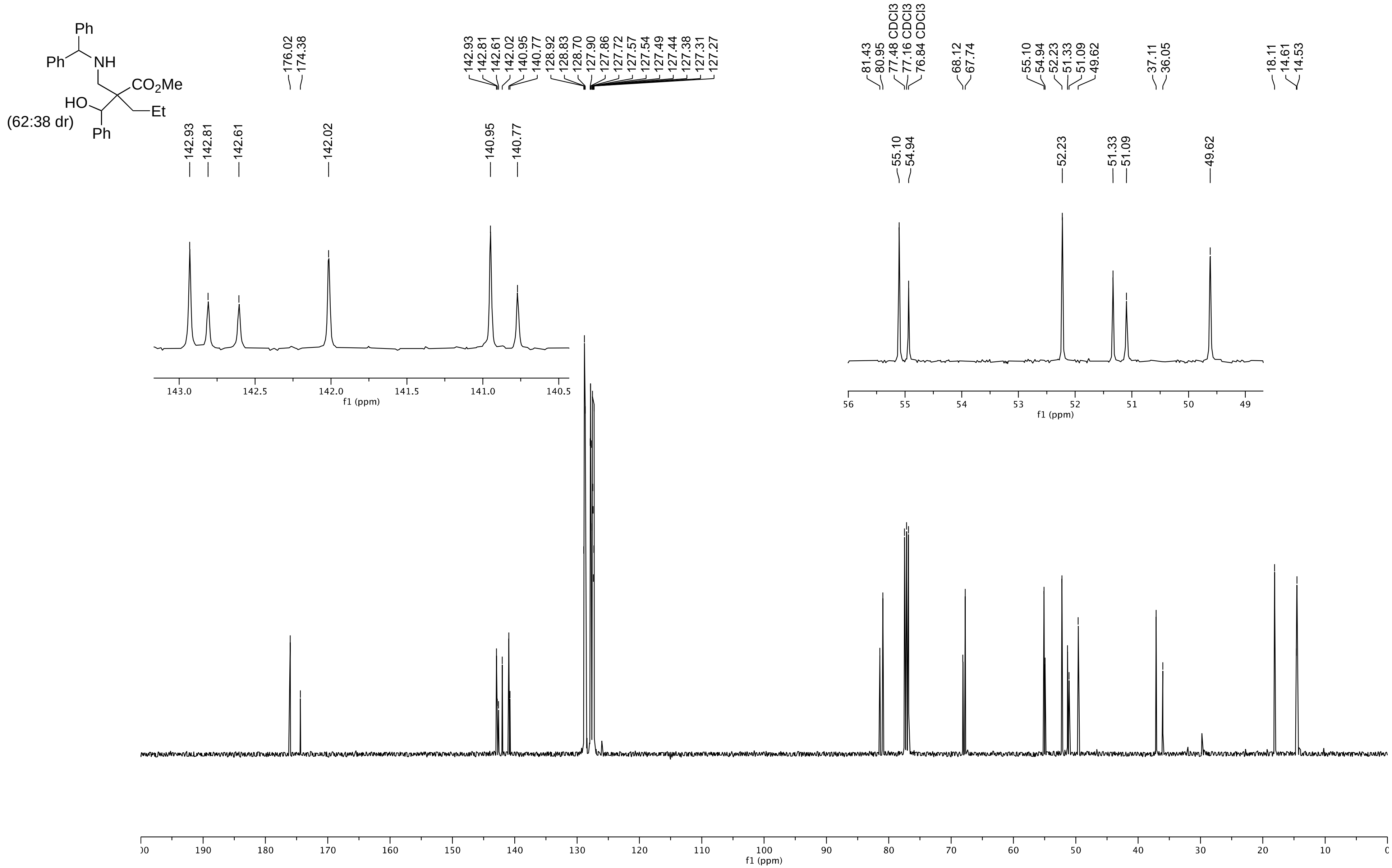

Methyl 2-((*tert*-butanesulfinyl)aminomethyl)-2-(hydroxy(phenyl)methyl)pentanoate (24) – ^{1}H NMR (400 MHz, $CDCl_3$)

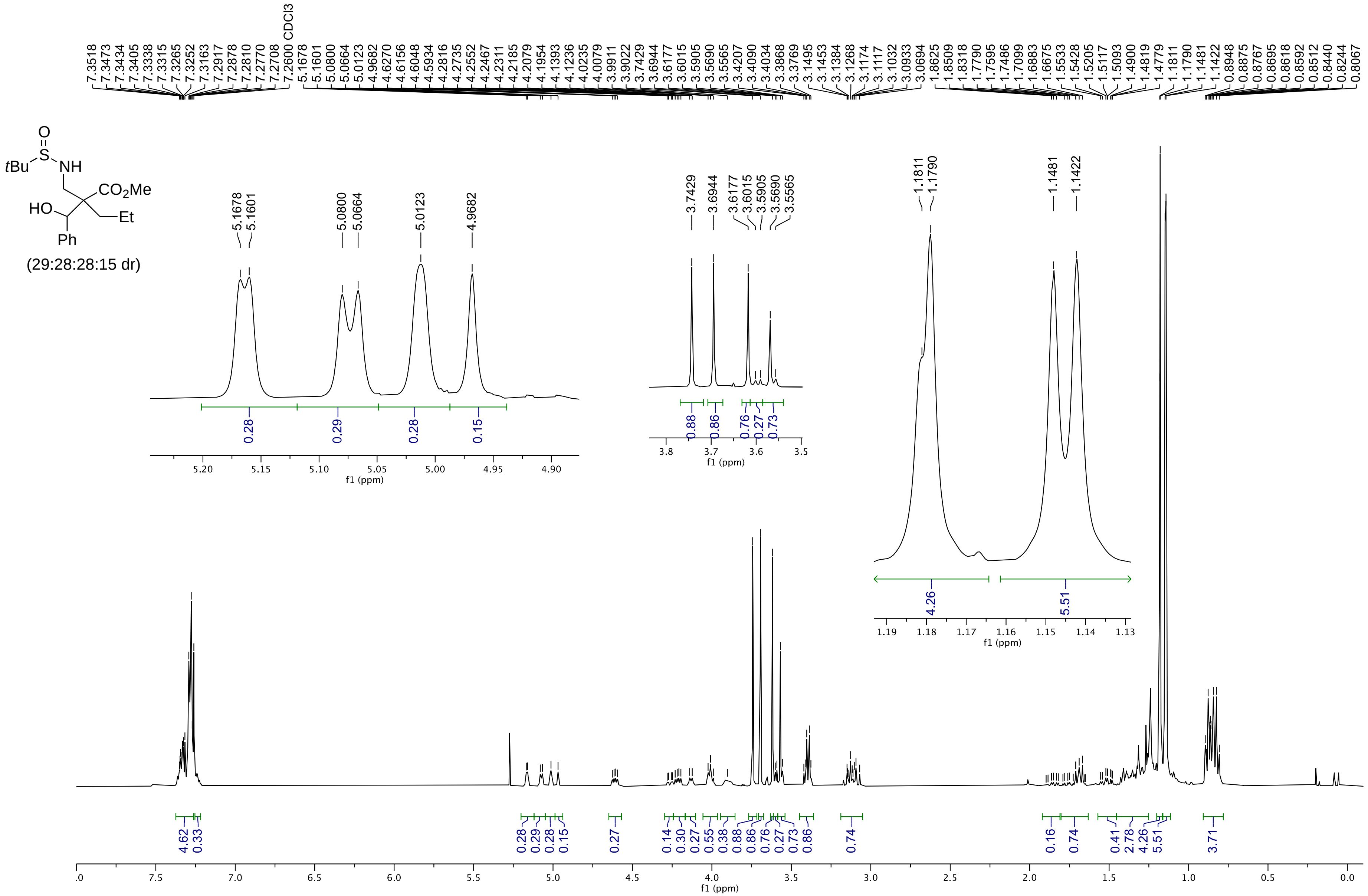

Methyl 2-((*tert*-butanesulfinyl)aminomethyl)-2-(hydroxy(phenyl)methyl)pentanoate (24) – ^{13}C NMR (100 MHz, $CDCl_3$)

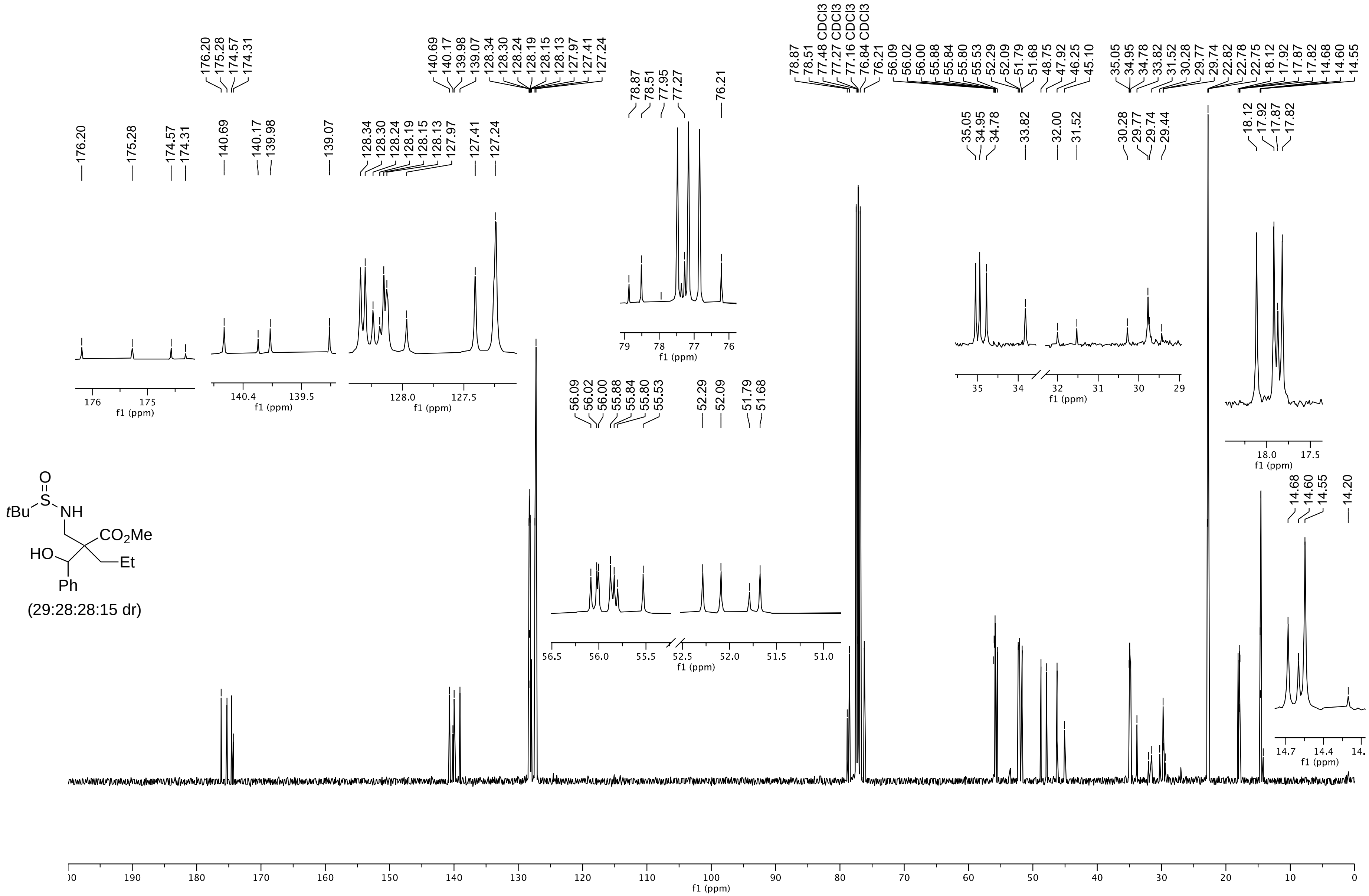

Methyl 2-((*tert*-butanesulfinyl)aminomethyl)-4-methylpentanoate (25a) – ^{1}H NMR (400 MHz, $CDCl_3$)

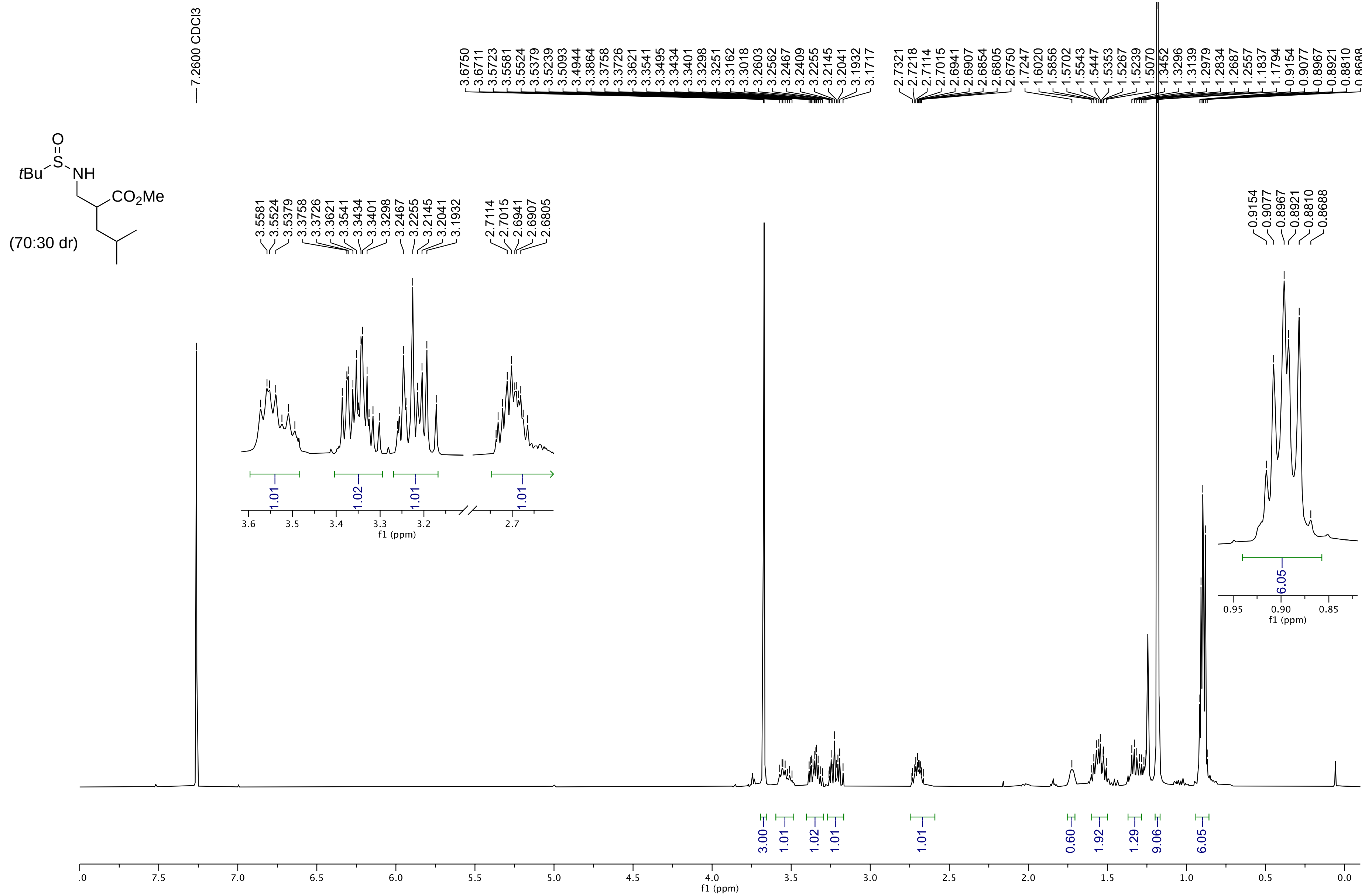

Methyl 2-((*tert*-butanesulfinyl)aminomethyl)-4-methylpentanoate (25a) – ^{13}C NMR (100 MHz, $CDCl_3$)

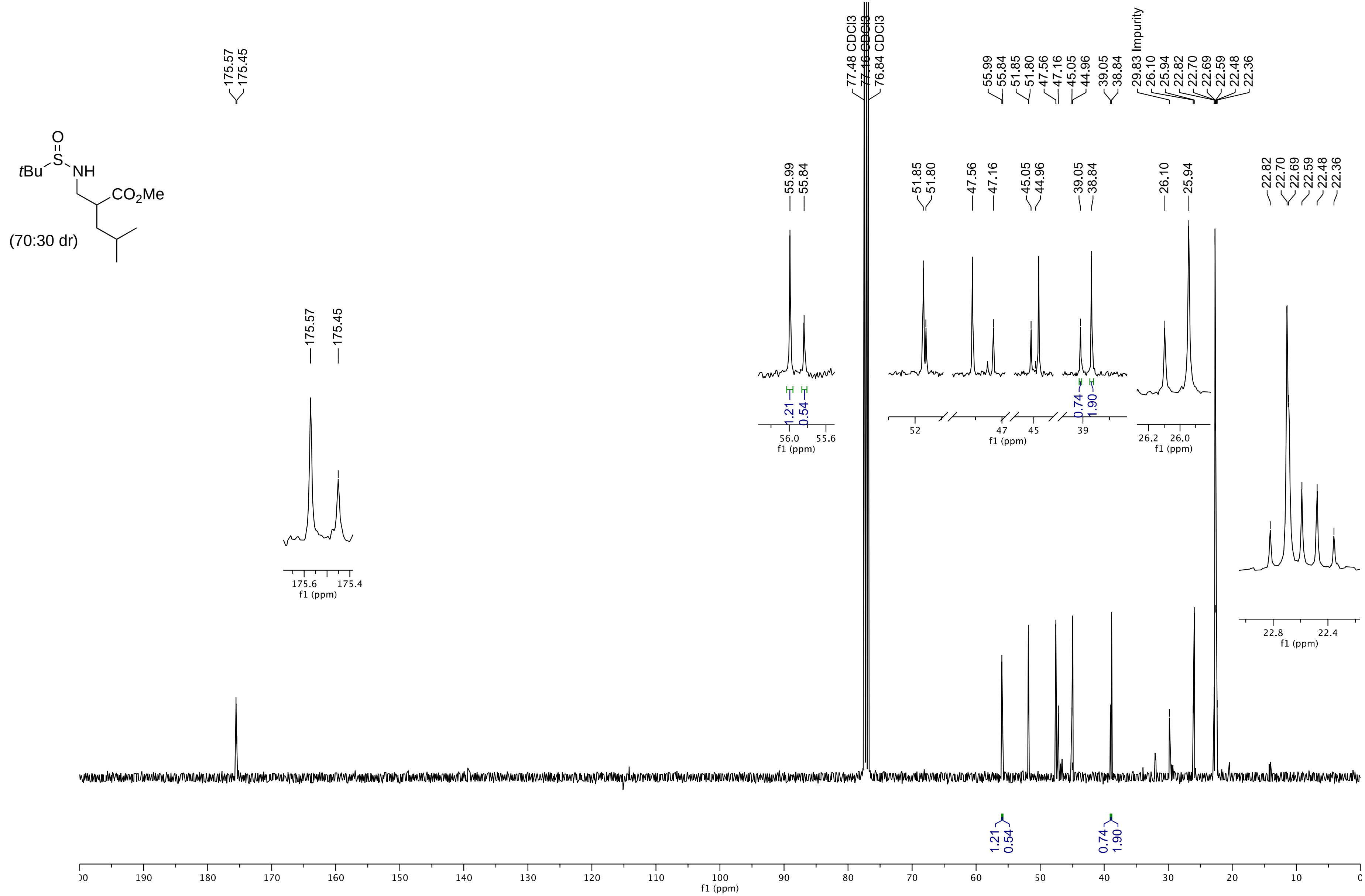

Methyl 2-[*N*-benzyl-*N*-*tert*-butanesulfinyl(aminomethyl)]-2-(hydroxy(phenyl)methyl)pentanoate (26) – ^{1}H NMR (400 MHz, $CDCl_3$)

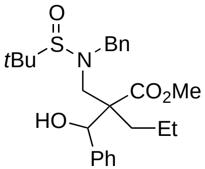

(49:25:23:3 dr)

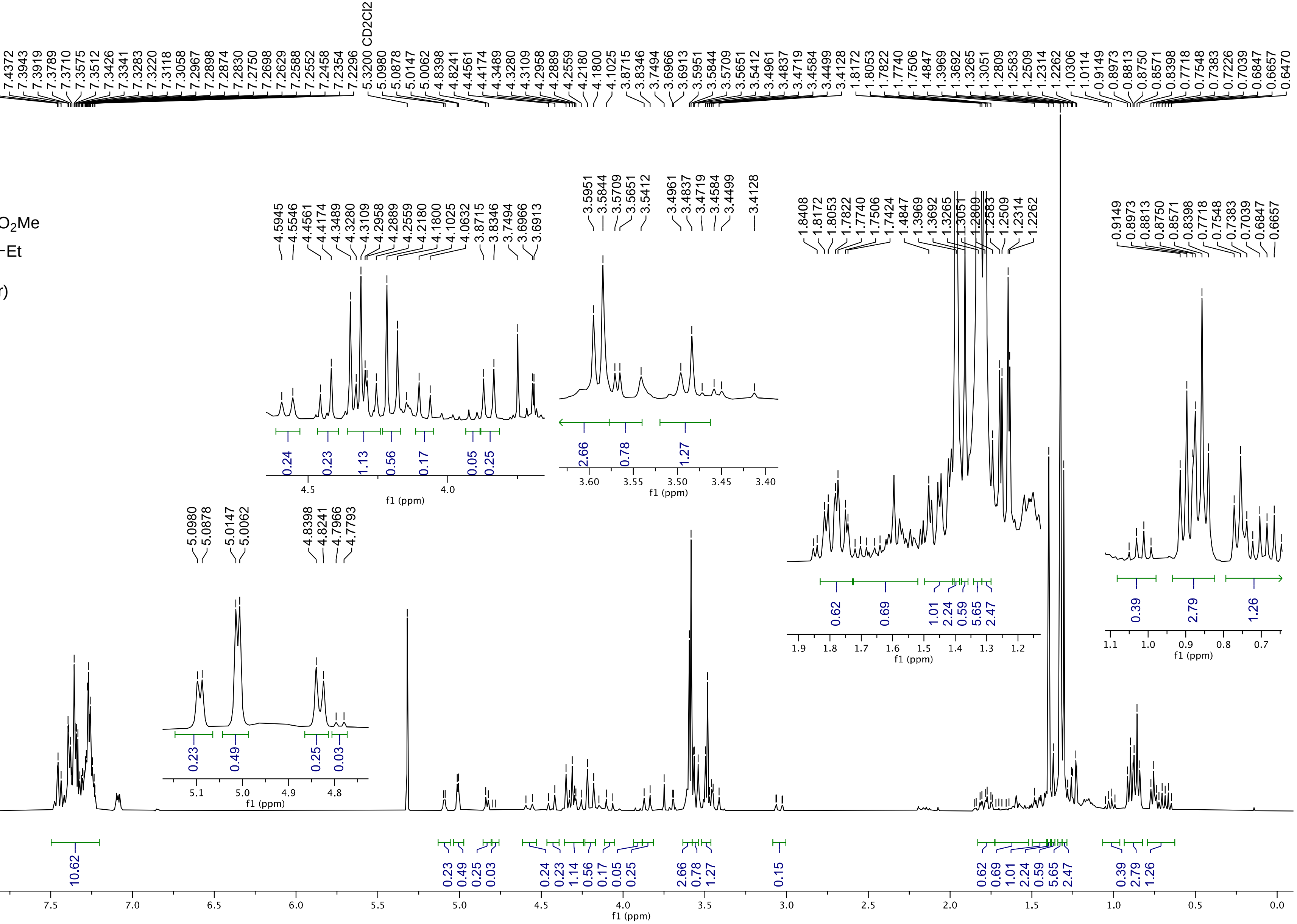

Methyl 2-[*N*-benzyl-*N*-*tert*-butanesulfinyl(aminomethyl)]-2-(hydroxy(phenyl)methyl)pentanoate (26) – ^{13}C NMR (100 MHz, $CDCl_3$)

(49:25:23:3 dr)

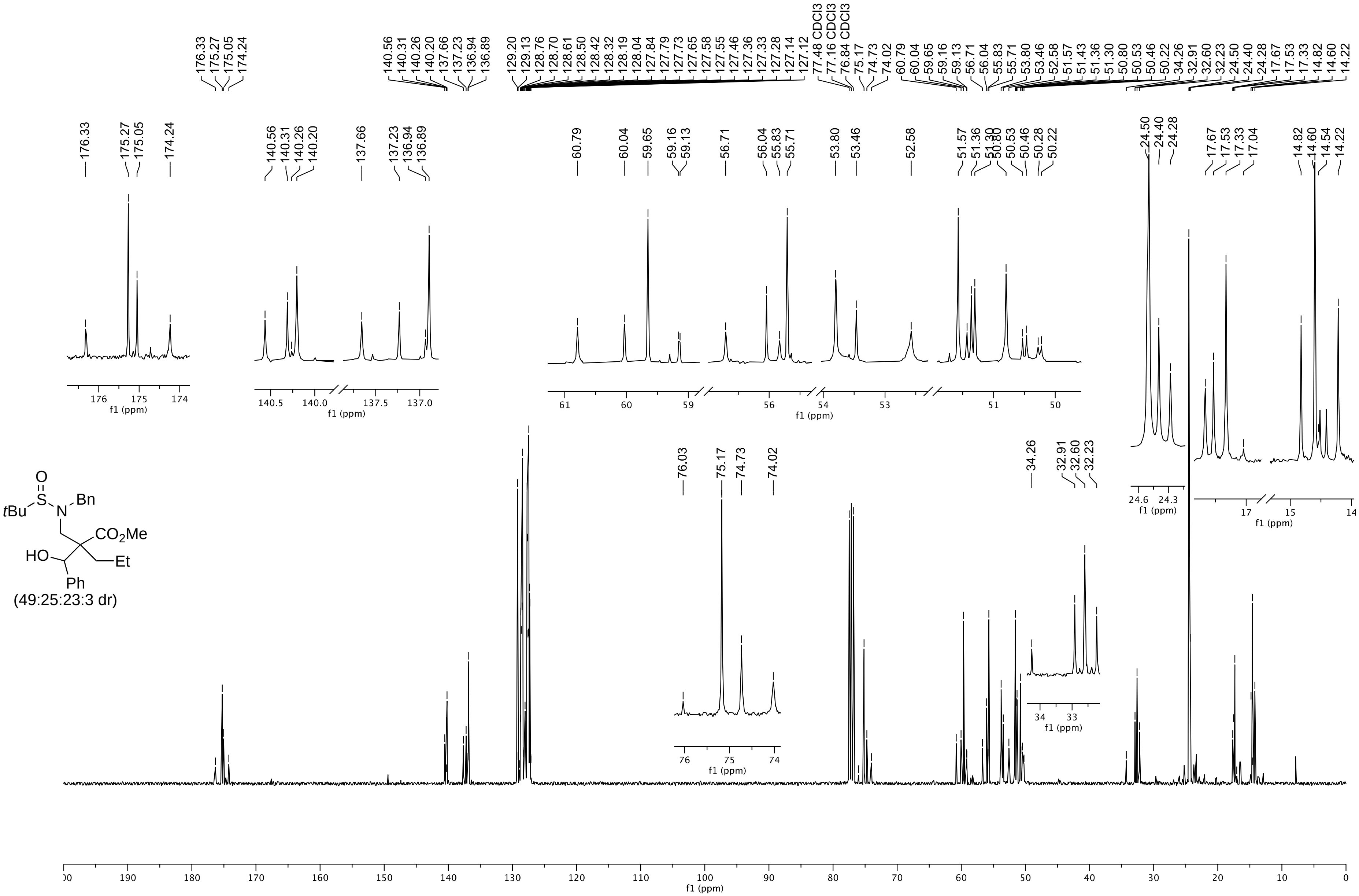

Supplement: File 1 — General information, characterization data, chemical correlation, and copies of NMR spectra. [file Beilstein_J_Org_Chem-19-1443-s001.pdf]
